# A distance-based framework for assessing the ex-situ conservation status of plants

**DOI:** 10.1371/journal.pone.0324820

**Published:** 2025-06-03

**Authors:** Marwa El Graoui, Michel Edmond Ghanem, Moez Amri, Robert J. Hijmans

**Affiliations:** 1 Agrobiosciences department, College of Agricultural and Environmental Sciences, Mohammed VI Polytechnic university, Benguerir, Morocco; 2 Department of Environmental Science and Policy, University of California, Davis, California, United States of America; 3 CIRAD, UMR AGAP Institut, Montpellier, France; 4 UMR AGAP Institut, Univ Montpellier, CIRAD, INRAE, Institut Agro, Montpellier, France; HUN-REN Centre for Ecological Research, HUNGARY

## Abstract

We present a framework to assess the ex-situ conservation status of plant species, by estimating the fraction of extant genetic diversity that has been conserved as seeds or other living material outside their natural habitat. We use a species distribution model to predict the area that is suitable for a species, and use inclusion and exclusion buffers to estimate its range. The range is divided into a number of zones that is proportional to the square root of its size. The geographic and environmental distance between zones is combined into a single distance metric that is used as to set weights on the links of a network connecting neighboring zones. An adjusted network is created by setting the distance between zones with seed samples to zero and halving the distance to neighboring zones for which there is no seed sample. The ex-situ conservation score (*XC*, between 0 and 1) is then computed as the reduction in the sum of the weights of the shortest paths between the nodes in the adjusted network relative to the sum the weights of the shortest paths in the original network. We adjust *XC* for small seed sample sizes and for the number of seed samples with unknown geographic origin. We illustrate our framework for 61 wild *Vigna* species in Africa. Twenty-three species were not conserved ex-situ (*XC* = 0), and 42 species had very low conservation scores (*XC* < 0.2). Range sizes were very different from the suitable area sizes predicted with the species distribution model (r = 0.09). The geographic and environmental distances were weakly correlated (r = 0.29), illustrating the importance of considering both. The Pearson correlation coefficient between *XC* and seed sample size for each species was 0.94, suggesting that seed sample size can be useful for quickly evaluating ex-situ conservation status.

## Introduction

The seeds of many plant species have been collected and deposited in “seed banks” for conservation and research. For crops and their wild relatives, there are specialized collections referred to as “gene banks” that are an important resource for crop research and breeding [1]. The conservation of these seeds, or other living materials, outside their natural habitat, is referred to as “*ex-situ* conservation” — as opposed to “*in-situ* conservation” where the habitat in which species of interest occur is protected. Given the rapid global change in land use and climate, it is important to understand and evaluate the ex-situ conservation status of plant species to inform priority setting for future collection as well as in-situ conservation actions [2].

In essence, we would like to measure the proportion of the overall genetic diversity of species, or other taxonomic groups, that is conserved in seed banks. As we generally do not have sufficient genetic data to compute that, proxies have been used. For example, the method proposed by Ramírez-Villegas et al. [3] and refined by Khoury et al. [4] uses three metrics with values ranging between zero and one. The first metric (geographic representativeness) estimates the fraction of the geographic range covered by the seed samples. The second metric (environmental representativeness) estimates the fraction of the environmental variability of the entire range that is represented by the seed samples. The third metric (sampling representativeness) is the ratio of the number of seed samples to all (seed, herbarium, and other observations) samples. The three metrics are averaged to compute the “final conservation score”. Hereinafter, we refer to this score and method as the *FCS*.

In this paper we present an alternative framework to assess the *ex-situ* conservation status of taxa. We predict the suitable area for a species and, to reduce error of commission and omission, we use inclusion and exclusion buffers around known occurrence locations to derive a range. We also consider that the genetic distance between plant populations tends to increase linearly with distance [5], and thus proportionally to the square root of range size. We use this relation to determine the number of sampling zones into which we divide a species’ range. Strong environmental gradients may lead to more genetic differentiation than expected by geographic distance alone. We express environmental distance in terms of geographic distance and sum these two distance measures into a single metric that serves as link-weights on a network that connects the sampling zones. We create an adjusted network to account for the zones for which we have a seed sample, and we compute the ex-situ conservation score *XC* as the reduction in the sum of the weights of the shortest paths between all nodes in the adjusted network, divided by the sum of the weights of the shortest paths between all nodes in the original network. We also consider a minimum sample size, and the seed-bank samples for which we do not have geographic coordinates of the collection locations.

Here we first describe our new framework in detail, and we then illustrate its use in a case study on the African wild species in the *Vigna* genus. We include a sensitivity analyses for two important parameters, and we compare our results with those obtained with the *FCS* method.

## Analytical framework

### Determine a species’ range

**Fig 1:**
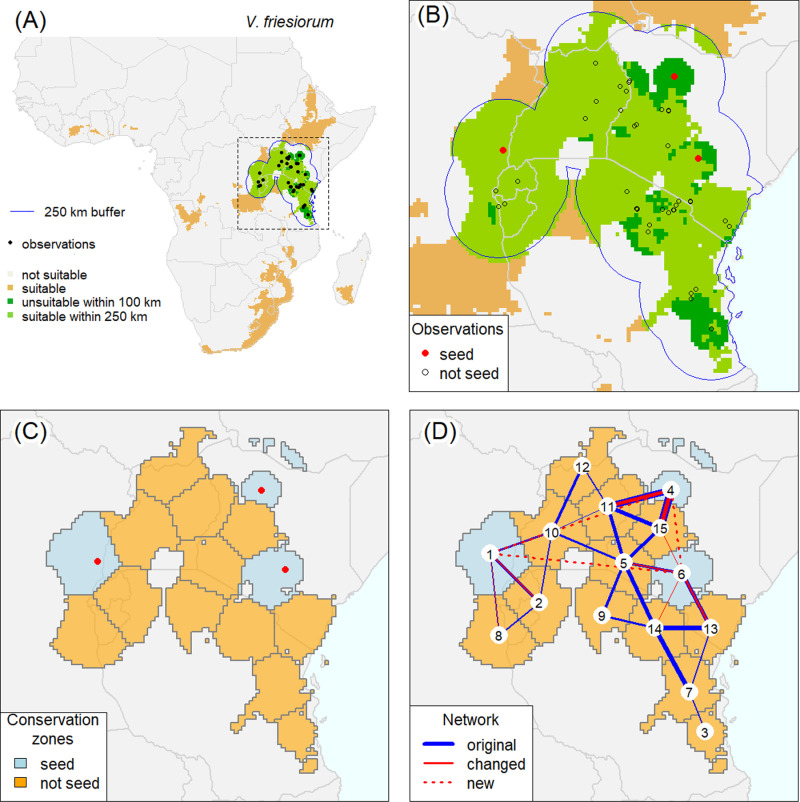
An illustration of major steps in the *XC* ex-situ conservation assessment framework, based on the occurrence records of *Vigna friesiorum*, but using hypothetical seed samples. A: species distribution model prediction for the entire region of interest showing areas that are suitable; suitable and inside a 250 km exclusion zone (areas further than that distance from an occurrence record are not considered part of the range); and unsuitable but within a 100 km inclusion zone (all areas within that distance from an occurrence record are included in the range). B: The same as A but only showing the region where the species occurs, and also showing seed bank and other occurrence records; C: 15 conservation zones computed with *k*-means clustering and the collection location and zones of the seed bank samples; D: Zone numbers and network links (edges) between neighboring zones that are used to compute the *XC* conservation score. The link width is proportional to the weights used to compute the distance between nodes. The blue lines show the original weights, and the red lines the adjusted weights after considering the three zones with seed samples; including the three new links with zero weight between these zones. See S1 Diagrams for similar figures for the species used in our case study. (Source. Country boundary data from gadm.org).

There are multiple techniques to estimate the range of a species from localities of known occurrence [6], that include buffering (or “circular area”, CA) [7] and species distribution models (SDM) [8]. SDMs compute the similarity of the environment at any location to the environment at locations with a species occurrence record. An optimal threshold can be computed to classify locations as “similar” and “dissimilar”. Areas with an environment similar to that of the localities with occurrence records are generally referred to as “suitable” for the species. This predicted suitable area may be biased by the spatial distribution of the occurrence records, potentially excluding areas where the species actually occurs. Moreover, the model may identify areas with a suitable environment that are far away from where the species occurs [9]. To address the problem of under- and overprediction we derived a species range by only including suitable areas that are within an exclusion buffer around all occurrence localities where the taxon was observed, thus reducing overprediction. We also use a smaller “inclusion buffer” around all occurrence localities to include areas where the species has been observed but have a somewhat atypical environment such that they are not considered suitable by the SDM. It is important to check such locations for errors and if deemed unreliable, they could be removed. This procedure is illustrated in Fig 1A, B.

### Zones

We propose to use the commonly found linear relationship between geographic and genetic distance [5,10] to divide the putative range of the species into “conservation zones”. When considering a circle, the farthest distance between two points on a circle of size *A* is its diameter, $2\sqrt{\frac{A}{\text{π}}}$ and, therefore, genetic diversity is expected to be proportional to $\sqrt{\frac{R}{\text{π}}}$, where *R* is the range size. The number of seed samples required for a perfect score thus increases with range size, but the sampling density required is higher for a species with a small range than for a species with a large range. The number of conservation zones that ideally should all be sampled can be computed with Equation 1, where $Z_{R}$ is the number of zones into which range with size *R* (km^2^) should be divided, and a scaling parameter *ω*.


$$Z_{R}=\max\left(1,\;\omega \;\sqrt{\frac{R}{\pi }}\;\right)$$ (1)

The value of *ω* is somewhat arbitrary, in this paper we use 0.025 (Fig 1C) and we evaluate additional values in a sensitivity analysis. *ω* = 0.025 implies that an area of 70.9 × 70.9 = 5,027 km^2^ would be divided into exactly one zone, and an area of 709 × 709 = 502,655 km^2^ would divided into exactly ten zones. If *ω* doubles, the sides of these squares halve (35 km for one zone if *ω* = 0.05) and they double if *ω* halves (142 km for one zone if *ω* = 0.0125). The zones are a tessellation of the range, that is, they are non-overlapping and there are no gaps between them within the range. Such zones can be computed with *k*-means clustering.

To get the highest conservation score there should be at least *η* seed samples in each zone. In this paper we use the simplest case of *η* = 1. The required number of locations, populations, or organisms sampled per zone could be increased to get a stronger coverage requirement. It would also be possible to increase *ω* and thus the number of zones. But subdividing the putative range into many relatively small adjacent areas would increase the chance of creating zones where the species does not actually occur.

### Conservation score

We then compute an *ex-situ* conservation score, *XC*, that expresses the reduction in geo-environmental distance when considering the zones with a seed sample, relative to the distance between all zones. Seed samples from two neighboring zones are likely more similar than those from two zones that are far apart. We account for this by creating a network that connects the centroids of all adjacent zones. (Fig 1D). If the range is not contiguous, such that there are geographic sub-ranges, these are connected to their nearest sub-range such that all zones are connected.

The geographic distance between zones is expressed as the shortest distance between the zones’ centroids. To account for the effect of environmental distance as well [11,12] we first fit a model that predicts the geographic distance from environmental distance. Such a model can be derived from the environmental and geographic distance data for *all* species in the group of interest (e.g., a genus) or for a set of sampled locations from the study area. The model can then be used to predict the expected (minimal) geographic distance between zones given their environmental distance. This measure can be added to (or averaged with) the geographic distance to create a single geo-environmental distance measure. The geo-environmental distance can be capped to a maximum to avoid that disjunct sub-ranges get too much influence on the conservation score.

The geo-environmental distance is used to give a weight to the network links (edges). This allows us to compute the shortest geo-environmental distance between a zone (node of the network) to any other zone. We refer to the sum of these distances as $D_{A}$. We then consider the zones for which seed samples are conserved by setting the distance between these zones to zero and halving the distance to neighboring zones for which there is no seed sample. This means that, where necessary, we add new links (with weight zero) to directly connect zones that were previously indirectly connected. If *η *> 1, the change in edge weight is inversely proportional to the number of seed samples in a zone if it is below *η*. We recompute the sum of the distances and refer to that number as $D_{S}$. The conservation score is then computed with equation2.


$$XC=1-\;\frac{{\;D}_{S}}{{\;D}_{A}}$$ (2)

### Adjustments

Adjustments may be needed to avoid giving high conservation scores to taxa with very small ranges and very low sample sizes. If the total number of seed samples n is below a minimum threshold $n_{\min}$, we suggest adjusting *XC* by multiplying it with the ratio of the actual and minimal sample size (equation 3) or, alternatively, a non-linear variation thereof.


$$\begin{array}{c} XC=XC\times min\left(1,\;\left(\frac{n\;}{n_{\min}}\right)\right) \end{array}$$ (3)

It may be important to adjust for missing georeferences. For example, if there are no seed samples that can be georeferenced, this does not mean that the species is not conserved at all. We suggest using the relationship between the number of georeferenced observations and *XC* across taxa to compute expected *XC* (*E*_*xc*_) given that sample size. *XC* can then be adjusted for the number of non-georeferenced samples according to equation 4.


$$XC=\max\left(XC,\;{\;E}_{XC}\right)$$ (4)

## Materials and methods

### Data collection and cleaning

We used the framework described in the previous section to evaluate the ex-situ conservation status of the wild species in the *Vigna* genus (Fabaceae) that occur in Africa. *Vigna* is a pantropical genus that includes over a hundred species, of which ten are cultivated. Sixty-one of the wild species occur in Africa across a wide geographic and environmental range [2,13].

*Vigna* occurrence records were obtained from GBIF [14] and Genesys [15] (S1 Table). Taxonomic names were standardized to accepted names according to the World Checklist of Vascular Plants [16]. Coordinates for the collecting locations were checked and those with swapped longitude and latitude values and with missing minus sign were fixed. We (re-)georeferenced collecting locations that had a locality description but no coordinates or with coordinates that were inconsistent with the country they were reported to be in. The occurrence localities of each species were visually inspected and checked for their plausibility of outliers with the published literature and online resources. Records from Genesys were classified as “seed” and records from GBIF as “not-seed”. GBIF also has seed collection records, but it is not possible to reliably determine which records are from seed conservation collections and which are from herbaria or other observations. Since including seed bank samples as “not-seed” samples as well should not affect our results, we were not concerned by this. *V. unguiculata*’s wild subspecies and varieties were all grouped and recorded as *V. unguiculata*.

### Species’ range

We used the MaxEnt species distribution modeling algorithm [17,18] with *R* packages “predicts” [19] and “terra” [20] to model the similarity of all grid cells to the grid cells in which the species had been observed. We used no more than one observation per grid cell. Climate data [21] at 10 minute spatial resolution were used as predictor variables. Seven out of nineteen bioclimatic variables were chosen: Annual temperature, Mean Temperature of Wettest Quarter, Mean Temperature of Driest Quarter, Annual precipitation, Precipitation Seasonality, Precipitation of Wettest Quarter, Precipitation of Driest Quarter. These were selected based on their importance to the models and their low collinearity [22]. MaxEnt predictions were classified as similar/dissimilar using the “equal sensitivity specificity” threshold.

We defined the species range as all areas that are within an inclusion buffer of 100 km of an observation and all areas classified as “similar” to the occurrence localities by the SDM if they were within an exclusion buffer of 250 km from an observation (Fig 1A, B and S1 Diagrams). We ran a sensitivity analysis to present how different buffer sizes in both inclusion and exclusion buffers influence the *XC* scores. We considered the following inclusion/exclusion buffer combinations: 0/0 km, 0/125 km, 0/250 km, 0/500 km, 50/125 km, 50/250 km, 50/500 km, 100/125 km, 100/250 km, 100/500 km, 200/250 km and 200/500 km.

### Zones

The number of zones ($Z_{A})$ for each species was computed based on its range size and using a value of 1/40 for scaling parameter *ω* in Equation 1. We created a tessellation with *k*-means clustering. We ran a sensitivity analysis to present how the number of zones influences the results by comparing the effect of setting *ω* at 1/20, 1/40, and 1/80.

The environmental distances between zones were computed from the mean annual temperature and the annual precipitation in each zone. We fit LOESS local regression models, for all species combined, between these environmental variables and geographic distances between zones. For each environmental variable we fit two models. The model residuals of the first model were used to remove outliers that had a geographic distance more than 250 km further than expected given the distance in temperature or precipitation. It is of course possible to find a similar temperature and rainfall conditions in places that are very far apart but we were not interested in that. We want to capture the typical distance needed to observe a certain amount of environmental change. The second model was used to transform the two environmental distances to its equivalent (minimum) geographic distance (Fig VI in S1 Figures), and these two distances were averaged. For each species, the geo-environmental distance was computed by summing the geographic distance and the transformed environmental distance.

We then created a network between the centroids of all adjacent zones and between the nearest centroids of otherwise disconnected parts of the species range using *R* package “igraph” [23]. Link weights were set to the geo-environmental distance between zones. These weights were capped at 1500 km to avoid that network distances would be too much influenced by links between disjunct zones.

We used these networks to compute *XC* for each species using functions implemented in *R* package “conexus” [24]. Because *k*-means clustering is a stochastic process, we ran the *k*-means algorithm, and all subsequent steps ten times and averaged the *XC* score. The conservation score was adjusted for low seed sample sizes with equation 3, using a minimum sample size of 10; and for non-georeferenced seed samples with equation 4.

### Comparison with the FCS method

We compared our results with the *FCS* method [25]. We used MaxEnt as described above to compute the entire suitable area of each species, without adjusting with the inclusion/exclusion buffers. We also computed the 50 km circular area (buffer) (CA_50_) range of the seed bank collecting locations of the species. We then determined (1) the “geographical representativeness” (GR) as the proportion of the suitable area that is covered by the CA_50_ range; (2) the “ecological representativeness” (ER) as the proportion of ecoregions [26] that intersect with the CA_50_ and the suitable area; and (3) the sampling representativeness score (SR) as the number of seed bank collection locations divided by the number of non-seed bank occurrence records. We capped this number at 1. We averaged these three scores to obtain the *FCS*.

## Results

### Observations

**Fig 2:**
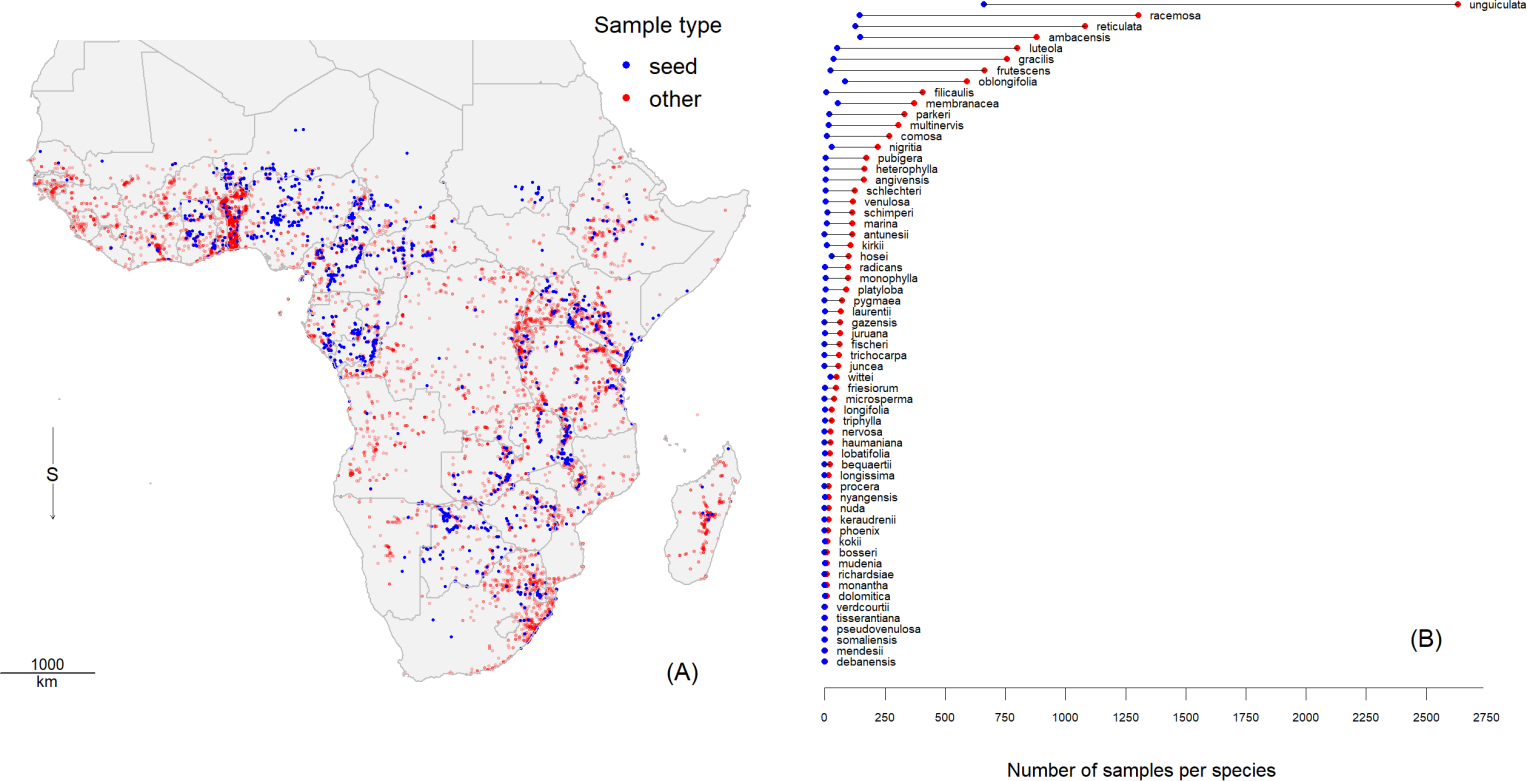
African wild *Vigna* species data available. (A) Locations of known occurrence and (B) the number of seed bank records and other records by species. (Source. Country boundary data from gadm.org).

The total number of accepted records for the 61 wild *Vigna* species with coordinates was 13,076, from which 1542 were seed-bank samples. From the original data, 54 records were removed because their coordinates were not on land. We also identified 309 outliers that showed inconsistencies in their localities and kept them as non-georeferenced points in the dataset. We identified and removed 546 duplicates. Wild *Vigna* species are distributed across Sub-Saharan Africa, except for the driest areas such as the Sahara and large stretches in Namibia (Fig 2A). The number of georeferenced unique localities per species ranged between 1 and 2631, with a median of 64. For *V. unguiculata* (2631), *V. racemosa* (1304) and *V. reticulata* (1083) we had more than 1000 observations each. The average number of seed bank records was 5.3% of the total number of records for each species (Fig 2B) (S2 Table). The number of seed bank records by species ranged between 0 and 661, with an average of 25.3 and a median of 1. For 45 species we had fewer than 10 seed bank records.

**Fig 3:**
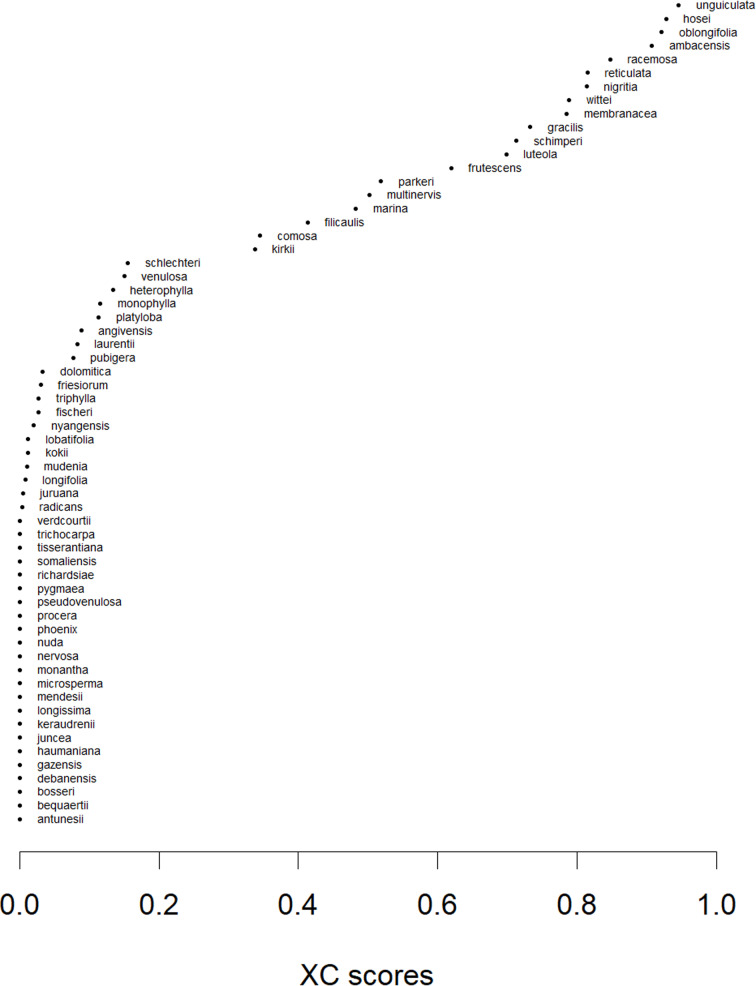
The *ex-situ* conservation status (*XC*) of African wild *Vigna* species. Species are sorted by their *XC* scores.

*XC* scores were > 0.5 for 15 species and > 0.9 for four species: *V.ambacensis*, *V. hosei*, *V. oblongifolia*, and *V. unguiculata*. Nine species had an *XC* between 0.1 and 0.5 and thirty-seven species had an *XC* below 0.1 (Fig 3; S2 Table). The *XC* scores were not very sensitive to the number of regions used (*ω* in formula 1; Fig I in S1 Figures). The average *XC* was 0.26 for *ω *= 1/80, 0.23 for *ω *= 1/40 and 0.21 for *ω *= 1/20. Pearson correlation coefficients between *XC* scores computed with these values for *ω* were > 0.98. The adjustment for non-georeferenced points marginally increased the *XC* of two species: *V. fischeri* (from 0 to 0.027) and *V. triphylla* (from 0.001 to 0.027) (S2 Table).

### Geo-environmental distance

**Fig 4:**
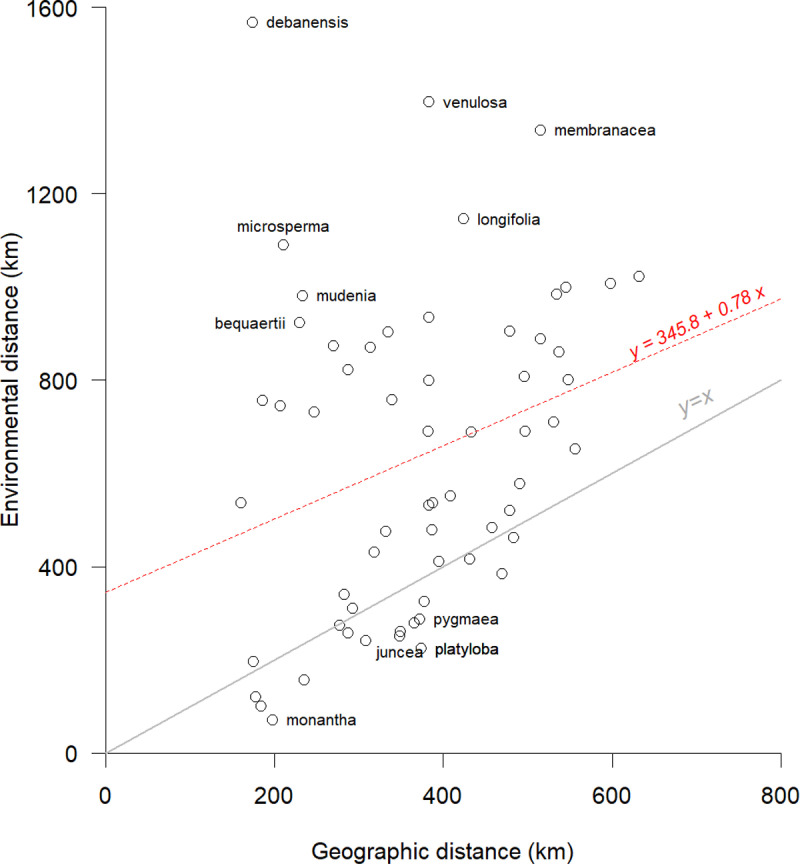
Average environmental and geographic distance for conservation zones of African wild *Vigna* species. Environmental distance is expressed in terms of expected geographic distance. The red dashed line represented the fitted regression model, and the gray line shows the identity line (*y = x*). Selected extreme cases are labeled.

There was only a weak Pearson correlation coefficient (r = 0.29) between the species level average geographic and environmental distances. Species with a much larger environmental distance than geographical distance included *V. debanensis*, *V. venulosa, V. membranacea* and *V. microsperma.* In contrast, *V. platyloba*, *V. monantha*, *V. pygmaea*, *V. juncea*, *V. nuda*, and *V. antunesii* had a somewhat lower environmental distance than expected based on the geographic distances between their zones (Fig 4 & S4 Table).

### Range size adjustment

**Fig 5:**
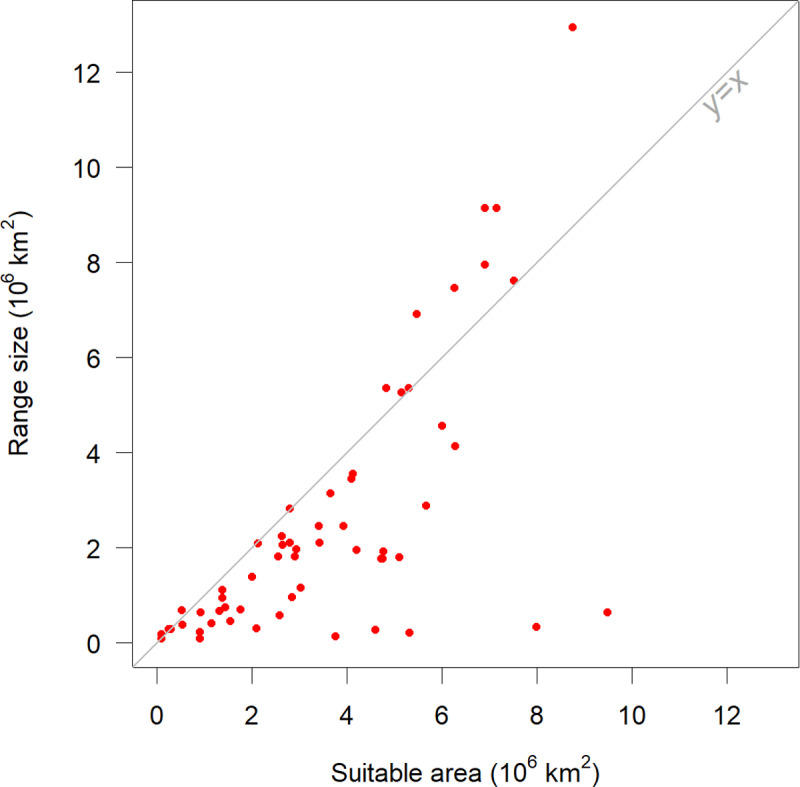
Estimated range size vs suitable area for wild *Vigna* species in Africa. Suitable area was predicted with a species distribution model. Range size was computed by adjusting the suitable area with a 100 km inclusion buffer and a 250 km exclusion buffer. Three species had suitable area of more than 30 × 10^6^ km^2^, covering the entire study region. This was an artifact of the SDM not being able to fit meaningful model for having very few occurrence localities, and these are not included in this figure.

The adjusted range sizes spanned from a 94,333 km² for *V. keraudrenii* to 9,216,420 km^2^ for *V. reticulata* and 12,969,542 km² for *V. unguiculata*; with a mean of 2,398,227 km^2^, and a median of 1,798,584 km^2^. Adjusting the suitable area with the inclusion and exclusion buffers change the range size between −96 and +87%. The average relative change was −33.7% and the median −31.3%. The largest reductions in range size (because of removing suitable areas far away from the places where the species has been observed) were for *V. tisserantiana* (−96.2%), and *V. monantha* (−95.8%) (S2 Table). A species with a very large increase in range size was *V*. *unguiculata* (+ 48%) because large areas in West Africa where the species clearly occurs were not classified as suitable (See Supplemental diagrams). The suitable area and the range size had a very low correlation coefficient (r = 0.09) (Fig 5).

Most species with an *XC* score above 0.5 had a range size between 2–10 × 10^6^ km^2^. Most species with a *XC* below 0.25 had a range size below than 2 × 10^6^ km^2^ (Fig II in S1 Figures).

The sensitivity analysis with twelve different inclusion/exclusion buffer size combinations showed a correlation coefficients for the *XC* scores of at least 0.95. The average *XC* score was lowest (0.19) when using no adjustment, and also relatively low when using no inclusion buffer and 500 km exclusion buffer (0.2) or a 250 km exclusion buffer (0.21). The highest average *XC* scores were observed for an exclusion buffer of 125 km and an inclusion buffer of 50 (0.24) (Fig III in S1 Figures).

### Sample size

**Fig 6:**
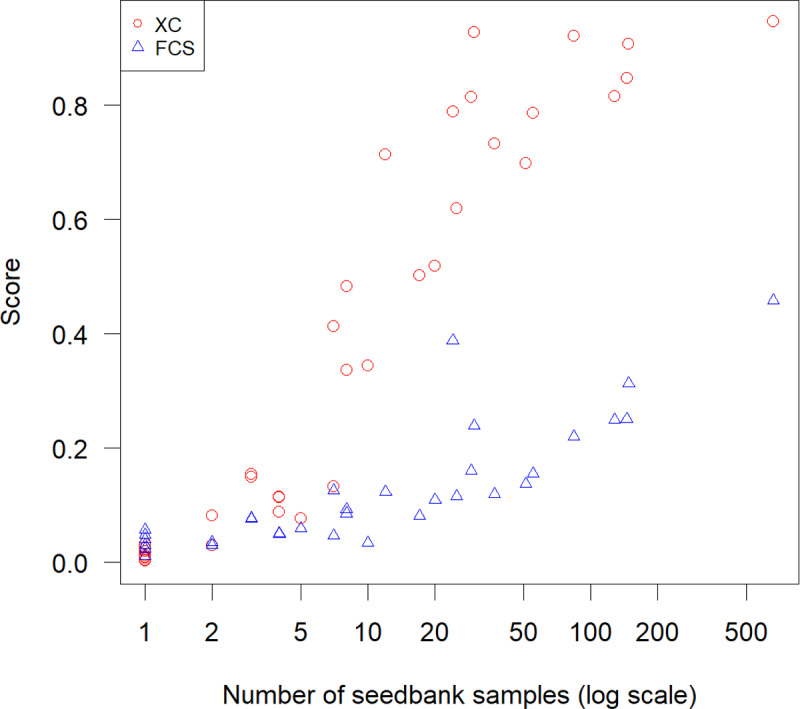
Ex-situ conservation score (*XC*) in relation to the number of seedbank samples for wild *Vigna* species in Africa.

The Pearson correlation coefficient between the *XC* and the natural log of the number of seed samples was 0.94 (Fig 6). Species with less than 10 seed samples had *XC* scores ranging from 0 to about 0.5. *XC* reached 0.85 for species with about 30 seed samples or more (Fig 6).

### Comparison with FCS

The FCS ranked from 0 to 0.45 (S2 Table). The highest score was for *V. unguiculata* that had 661 seed samples and an *XC* of 0.95. The scores for *V. oblongifolia* were also strikingly different. We had 84 seed sample records for this species that has a range that spans much of Sub-Saharan Africa. The *FCS* was 0.22 and the *XC* was 0.92. The *FCS* score was mainly low due to the low geographic representativeness score of 0.065 (S2 Table).

The Pearson correlation coefficient between the *FCS* and *XC* scores was 0.89 and the Spearman rank correlation was 0.98 (Fig IV in S1 Figures). For fifeteen species, *XC* was one to eight ranks higher than *FCS*, and for fourteen species *FCS* was one to seven ranks higher than *XC*. The species with the largest change, *V. comosa*, had an *FCS* of 0.034 and rank of 31, and a *XC* of 0.34 and rank of 44 (Fig IV in S1 Figures). Species with a much lower *XC* score included *V. kokii* went from rank 35 in *FCS* to rank 28 in *XC*, followed by, in order of decreasing differences (*FCS* rank, *XC* rank), *V. mudenia* (39, 27)*, V. wittei* (60, 54), and *V. pubigera* (41, 35).

The correlation coefficient between the number of ecoregions (environmental metric used for computing the *FCS* score) occupied by seed samples and the geo-environmental distance was 0.46 (Fig V in S1 Figures; S3 Table; S4 Table). The sampling representativeness score had a considerable impact on the *FCS* (S2 Table). For instance, *V. wittei* had a geographical representativeness score of 0.03, environmental representativeness score of 0.21, and a sampling representativeness score of 0.92, which resulted in an *FCS* of 0.39 (S2 Table).

## Discussion

We described a new distance-based framework to assess the *ex-situ* conservation status of plant species and illustrated this method using African wild *Vigna* species. A key element of our method is that we consider that genetic distance between populations is generally associated with the geographic distance between them. We divided the species range into contiguous zones and developed a method to expressing environmental distance between zones in terms of expected geographic distance, such that geographic and environmental distance can be expressed in a single geo-environmental distance metric. We used a network that connects adjacent zones, using the geo-environmental distance as link-weights, and computed *XC* as proportional reduction in network distance weight, after accounting for ex-situ conservation.

The *XC* score is inspired by the phylogenetic diversity (PD) metric [27]. Phylogenetic diversity is computed as the relative decrease in the summed branch lengths of a phylogenetic tree, after setting branch length for conserved species to zero. *XC* expresses the relative decrease in summed network distances after considering conservation. We used a network because it is a more direct representation of geographic data than a tree structure, and because it avoids the data loss associated with creating a tree from a distance matrix.

The weak correlation between geographic and environmental distances suggests that there is scope for more research to explore ways to combine geographic and environmental distance into a single variable to explain genetic distance between populations. Future work could also consider modeling geographic distance based on landscape resistance, that is, to account for environmental barriers that would make the likely gene flow paths deviate from the shortest path [28,29].

We considered a minimum number of seed samples required for a high conservation score. If a species’ range is very small; the seed samples for a species with a high score should ideally be from multiple populations, even if they occur near each other. In that case, if a species is only known to occur in a single locality it may not be possible to reach a high conservation score. Cases like this could be adjusted after computing the conservation score in a prioritization context. For example, the likelihood of finding more seed samples could be evaluated considering the overall sampling effort for a genus in the general area of occurrence of the species.

Determining the geographic range of a species is a key step in assessing its ex-situ conservation status. The best way to determine a geographic range will depend on the type and quality of available data. In some countries observation networks are so comprehensive that it is not needed to use modeling to determine a species range. We used SDMs and adjusted the predicted area of suitability with inclusion and exclusion buffers. This led to large changes in range size relative to the area of suitability, underlining the need to inspect the SDM predictions, and possibly adjust them for omission and commission error, especially in large regions where suitable environments may exist very far from where the species occurs.

We used 100 km inclusion buffers around observations to avoid omission error and 250 km exclusion buffers to avoid commission error. These buffer sizes are somewhat arbitrary and could vary with the extent of the study area and other aspects of the data. Our sensitivity analysis showed that *XC* is not very sensitive to the exact buffer sizes, as long as a reasonable exclusion buffer is used. The exclusion buffer size could be made adaptive to the number of observations of other species. That is, if there is a higher number of observations for closely related species in an area, it becomes less likely that the focal species occurs there if it was not observed. Even if the environment is suitable, the species may be absent due to dispersal limitation or interspecific competition [30,31]. Other approaches, such as buffered convex polygons around occurrence locations have also been employed [13]. That may work well in some instances, but convex polygons are highly sensitive to geographic outliers and the shape of the range. Moreover, if there are disjunct populations it may greatly overestimate the actual range. Inclusion buffers could be made adaptive to the SDM similarity score, using a lower threshold than used for suitable/unsuitable, to avoid including area that are clearly unsuitable.

The *XC* method has a few other parameters in addition to the inclusion and exclusion buffer. These include ω, which is used to determine the number of zones to create given a range size. We do not have a good reason to prefer a particular value, but fortunately our method was not very sensitive to this parameter either. This is because to get a high score, zones that are far away from each other need to be covered. Having a somewhat lower or higher number of zones does not change much in that computation.

### *Vigna* conservation

Most of the African wild *Vigna* species are poorly conserved in seed banks. Forty-two species had *XC* ≤ 0.2 and could be considered a priority for collection and conservation; but in an actual priority setting other factors such as accessibility, efficiency (visiting areas where multiple species can be collected), and perceived value of the species in crop breeding [32] could play an important role as well. Our method could be used to compute the expected change in *XC* arising from alternative collecting expeditions, and it could also be adapted to evaluate the in-situ conservation status of species.

Van Zonneveld et al [13] assessed the conservation status of wild and cultivated African *Vigna* species by comparing the spatial distribution of taxonomic richness computed from seed collections with richness computed from herbaria data. They identified geographic sampling gaps as areas with a high taxonomic richness and a low coverage of samples in seedbanks and concluded that the following where highly underrepresented *Vigna* species in seed banks (with our *XC* scores in parenthesis): *V. keraudrenii* (0), *V. monantha* (0), *V. somaliensis* (0), *V. gazensis* (0), *V. bosseri* (0), and *V. mendesii* (0); illustrating that very different method can identify the same (extreme) cases.

Maxted et al [2] reported that *V. comosa* subsp. *abercomensis*, *V. debanensis*, and *V. dolomitica* were the most threatened in the Red List of *Vigna* species. In our analysis *V. comosa* (the entire species) had rank 44 (out of 61; a low number is a high priority for conservation), *V. debanensis* had rank 12 and *V. dolomitica* had rank 34. This is a large discrepancy, but these authors already indicated that the Red List assessment is not a good reflecting the proper status of threat and conservation of these species [2].

### Comparison with the FCS method

In both the *XC* and the *FCS* methods, the entire range needs to be sampled to obtain the highest score. In the *FCS* method, this is implemented as follows. Each sample is assumed to cover a circle with a radius of 50 km, that is a zone of 7854 km^2^. Since overlapping areas are counted only once, the actual minimum number of seed samples needed to cover the entire range will always be higher than R/ 7854 km^2^, where *R* is the range size. The underlying assumption in the *FCS* that there is a linear increase of diversity with range size is questionable, as genetic distance generally increases linearly with distance, that is, diversity is more likely to be proportional to the square root of range size. The *FCS* will therefore overestimate the seed-sample size required for species with a large range as was illustrated for some of the best conserved species, such as *V. unguiculata* that had 661 samples and an *XC* of 0.95 but a *FCS* of only 0.45 and *V. oblongifolia* that had 88 seed samples and an *XC* of 0.93 and an *FCS* of 0.2. Overestimation of the number of seeds required is also expected because the *FCS* does not adjust SDM predictions by excluding suitable areas that are far away from the areas where a taxon is known to occur.

The *FCS* method uses a separate measure for environmental distance using ecoregions [33]. The use of ecoregions to assess environmental variation is suboptimal as the actual environmental distances between locations within a single ecoregion could be much larger than between nearby locations that are across ecoregion borders. The number of ecoregions covered may not be a good indication of the amount of environmental variation covered, as the environment of a pair of two ecoregions may be very similar or a very different. We note, however, that the use of ecoregions can have the benefit that it may implicitly account for the degree to which the seed samples are geographically spread out; which is something desirable that is built into the *XC* through the creation of conservation zones.

We did not consider the third *FCS* metric, the sampling representativeness, which is computed as the ratio of seed bank to other (herbarium) records. This score had a considerable impact on the *FCS* for *Vigna*, even after correcting it by capping it at one*.* We did not incorporate an equivalent metric in the *XC* method, as we do not consider it to be relevant: the *ex-situ* conservation status of a species (the proportion of extant genetic diversity in seed banks) is not affected by the number of known herbarium samples, and it should not go down if the number of herbarium records increases. Any future work that employs the *FCS* method should not include this third metric.

### Data quality

The type of data analysis described in this paper requires good quality occurrence data for seed samples and other records [2]. GBIF is an indispensable resource for species distribution data, but it is a federated database with many providers, and GBIF exercises limited quality control. Specifically for this type of work, it is important to know whether a record refers to a living seed sample or something else such as herbarium and/or observation. GBIF has as “basis of records” field with six valid possible entries, including “living specimens”. However, many genebanks use the ambiguous term “occurrence” for seed samples and several previous studies have described this as a synonym to “living specimens” [4,34–36]. We noted that for the *Vigna* species we worked on this would clearly be wrong. We found thousands of “occurrence” records for *Vigna* that in fact refer to herbarium specimens and this finding casts some doubt on the results reported by these prior studies. We therefore used records in the Genesys database as the source of “seed” samples. Whereas there may be more seed samples than reported by Genesys, the genebanks that do not have their data in Genesys are also least likely to supply seed upon request; and the long-term conservation of these samples may also be less certain. In fact, one could argue that to say that seed from a location has been conserved, there should be a sample in at least two collections in different countries; or a backup copy in another country, such as in the Svalbard Global Seed Vault.

Data cleaning is a challenging task, and currently, there are limited options for community contributions to enhance data quality. Geographic outliers can be difficult to handle with certainty. They may be caused by inaccuracies in geo-referencing and other errors such as taxonomic misclassification, but if they are correct they may of great interest. Most observations do not have associated Internet accessible images, and even with such images it may be a very challenging task to determine whether a record was correctly classified.

We found that many records are duplicated in GBIF; but these can be hard to detect given the absence of standard collector numbers. For example, it is common to find different location coordinates, from different georeferencing efforts, for records representing the same samples. This could be addressed by assigning globally unique identifiers (IDs) to samples. The Treaty for Plant Genetic Resources for Food and Agriculture has assigned DOIs to some seed samples, and in principle such as system could be expanded or complemented for other sample types. This would facilitate the creation of data curation and cleaning processes that not only enhance the original data but also ensure the continuous curation of improved data that would allow for updating cleaned and compiled databases. The current dire situation is essentially that each project starts from scratch because there is no easy way to update cleaned compiled databases.

### Outlook

The *XC* is especially useful for species with few seed samples and many other samples such that parts of the full range from which no seed samples have been collected can be identified. The species with most seed samples had the highest *XC* scores, suggesting that the number of seed bank samples can be a robust and very simple first estimate of *ex-situ* conservation status.

The *XC* scores reflect relative rather than absolute conservation status. Genetic data were unavailable for our samples, but it would be of interest to compare the geo-environmental distance estimates with genetic distance estimates for taxa for which such data are available [12]. This would allow for a more objective setting of some of the parameter values in our model. It could also allow for interpreting the *XC* between different genera, as some may encompass significantly more diversity than others.

The actual distribution of genetic diversity on the landscape is often far more complex than our method assumes. Species with large ranges may have undergone recent expansions and some parts of their range might not be as genetically diverse as those with smaller ranges. Genetic diversity within a species may also be concentrated near former refugia [37]. Although we lack detailed knowledge of such patterns for most species, these could potentially be modeled [38] and incorporated into the *XC* approach. Species traits could also be used to set *XC* parameters. For example, species that are wind-pollinated, and that have seeds that are easily transported by wind are expected to have a smaller increase in genetic distance per unit geographic distance than species that are insect pollinated and have heavier seeds [39,40]. Parameter ω could be reduced for species that are expected to have a relatively small increase in genetic distance per unit geographic distance, but then the number of samples per zone should probably be increased because a weaker geographic gradient does not imply that the same fraction of a species’ genetic diversity can be captured with a smaller sample. Explicit accounting for the degree of within-population genetic differentiation [41] could also be considered.

## Supporting information

S1 Diagrams The major steps to compute *XC* for 61 African wild *Vigna* species. (DOCX)

S1 Figures Supporting figures I to VII. (DOCX)

S1 Table Data of wild *Vigna* species occurrences. Abbreviations: “sample_type” = seed or non-seed.
(CSV)

S2 Table Species level statistics of African wild *Vigna* species. Abbreviations: “nseed” = number of georeferenced seed sample records; “nother” = number of georeferenced non-seed sample records; “nall” = total number of georeferenced records; “sdm_range size” = raw SDM predicted range size in km²; “adjusted_range size” = size of the inclusion/exlusion buffers adjusted range in km²; “relative_change_range” = relative change of range size between the raw and adjusted range sizes (%); “XC” = XC scores. “XCadj” = XC scores adjusted by accounting for non-georeferenced seed samples; “GRex” = FCS/geographical representativeness score; “ERex” = FCS/ecological representativeness score; “SRex” = FCS/sampling representativeness score; “FCS” = final conservation scores of the FCS method; “rank_XC” = ranks of XC scores; “rank_FCS” = ranks of FCS scores; “nogeoref_seed” = number of non-georeferenced seed samples.
(CSV)

S3 Table Statistics on the number of ecoregions covered by wild *Vigna* species’ seedbank samples, their temperature and precipitation ranges. Abbreviations: “necoregions” = Number of ecoregions covered by seed samples; “range_temp” = Temperature range of seed samples (°C); “range_prec” = Precipitation range of seed samples (mm).
(CSV)

S4 Table Environmental versus geographic distance for African wild *Vigna* species. Environmental distance is expressed in terms of expected geographic distance. Abbreviations: “dst” = sum of geographic and transformed environmental distance; “geodst” = geographical distance; “envdst” = environmental distances expressed as expected geographic distance.
(CSV)
