# Supplementary material for: A distance-based framework for assessing the ex-situ conservation status of plants
Source: PLoS One. 2025 Jun 3;20(6):e0324820. doi: 10.1371/journal.pone.0324820 (PMC12133166; doi:10.1371/journal.pone.0324820)

# Supporting information for El Graoui et al., A distance-based framework for assessing the ex-situ conservation status of plants

Diagrams to illustrate the major steps to compute *XC* for 61 African wild *Vigna* species. A: species distribution model (SDM) prediction for the entire region of interest showing areas that are suitable; areas that are suitable and inside the 250 km exclusion zone; and areas that are not suitable but within a 100 km inclusion zone. B: The same as A but for the region where the species occurs, also showing seed bank and other occurrence records; C: Conservation zones computed with *k*-means clustering and the collection location of the single seed bank sample for this species; D: Network connections between adjacent zones that are used to compute the conservation score. (This figure is our own work. Not copyrighted. GADM data is available under the CC-BY open license. https://gadm.org/license.html)

The *Vigna* species included (followed by the page number) are: *ambacensis* (2), *angivensis* (3), *antunesii* (4), *bequaertii* (5), *bosseri* (6), *comosa* (7), *debanensis* (8), *dolomitica* (9), *filicaulis* (10), *fischeri* (11), *friesiorum* (12), *frutescens* (13), *gazensis* (14), *gracilis* (15), *haumaniana* (16), *heterophylla* (17), *hosei* (18), *juncea* (19), *juruana* (20), *keraudrenii* (21), *kirkii* (22), *kokii* (23), *laurentii* (24), *lobatifolia* (25), *longifolia* (26), *longissima* (27), *luteola* (28), *marina* (29), *membranacea* (30), *mendesii* (31), *microsperma* (32), *monantha* (33), *monophylla* (34), *mudenia* (35), *multinervis* (36), *nervosa* (37), *nigritia* (38), *nuda* (39), *nyangensis* (40), *oblongifolia* (41), *parkeri* (42), *phoenix* (43), *platyloba* (44), *procera* (45), *pseudovenulosa* (46), *pubigera* (47), *pygmaea* (48), *racemosa* (49), *radicans* (50), *reticulata* (51), *richardsiae* (52), *schimperi* (53), *schlechteri* (54), *somaliensis* (55), *tisserantiana* (56), *trichocarpa* (57), *triphylla* (58), *unguiculata* (59), *venulosa* (60), *verdcourtii* (61), and *wittei* (62).


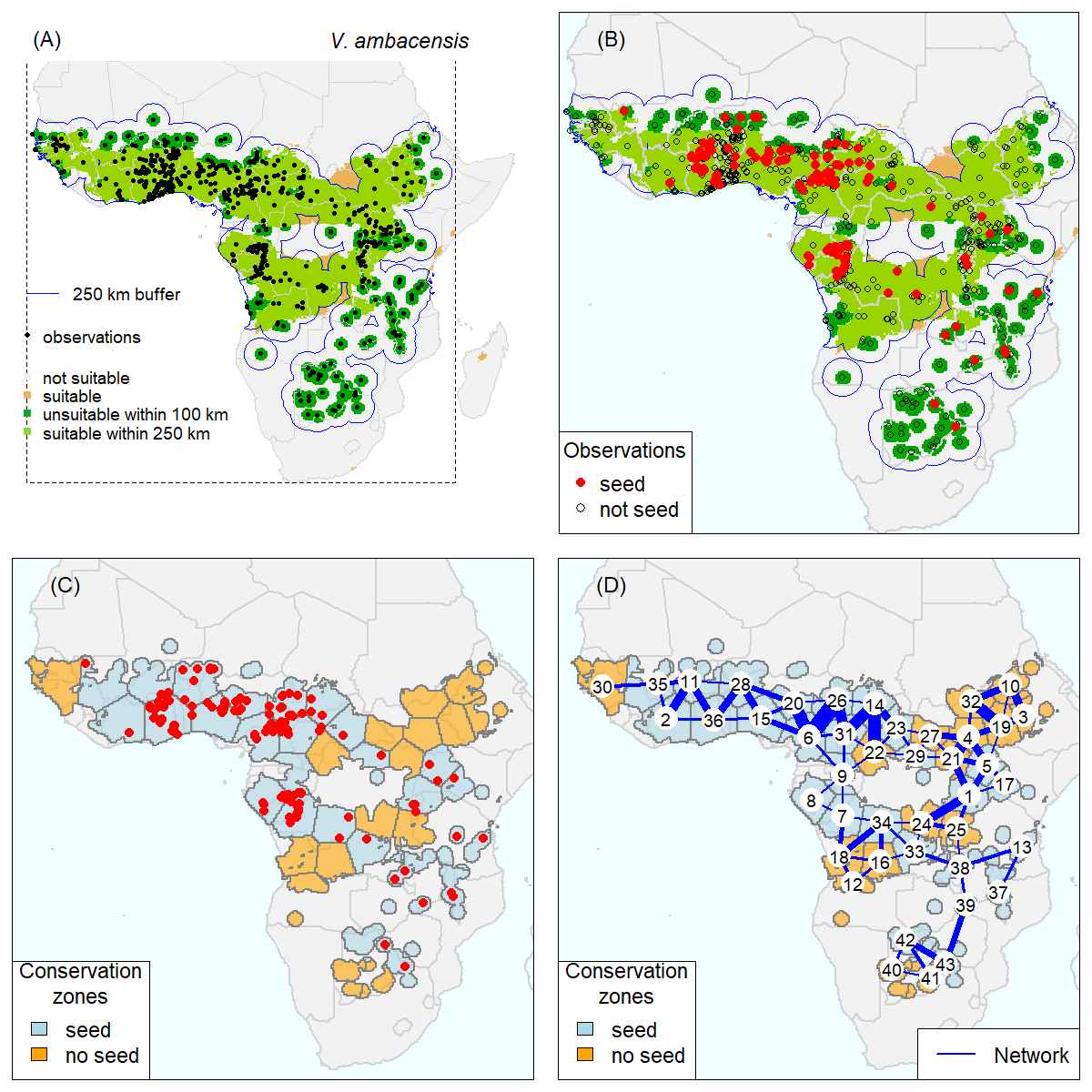

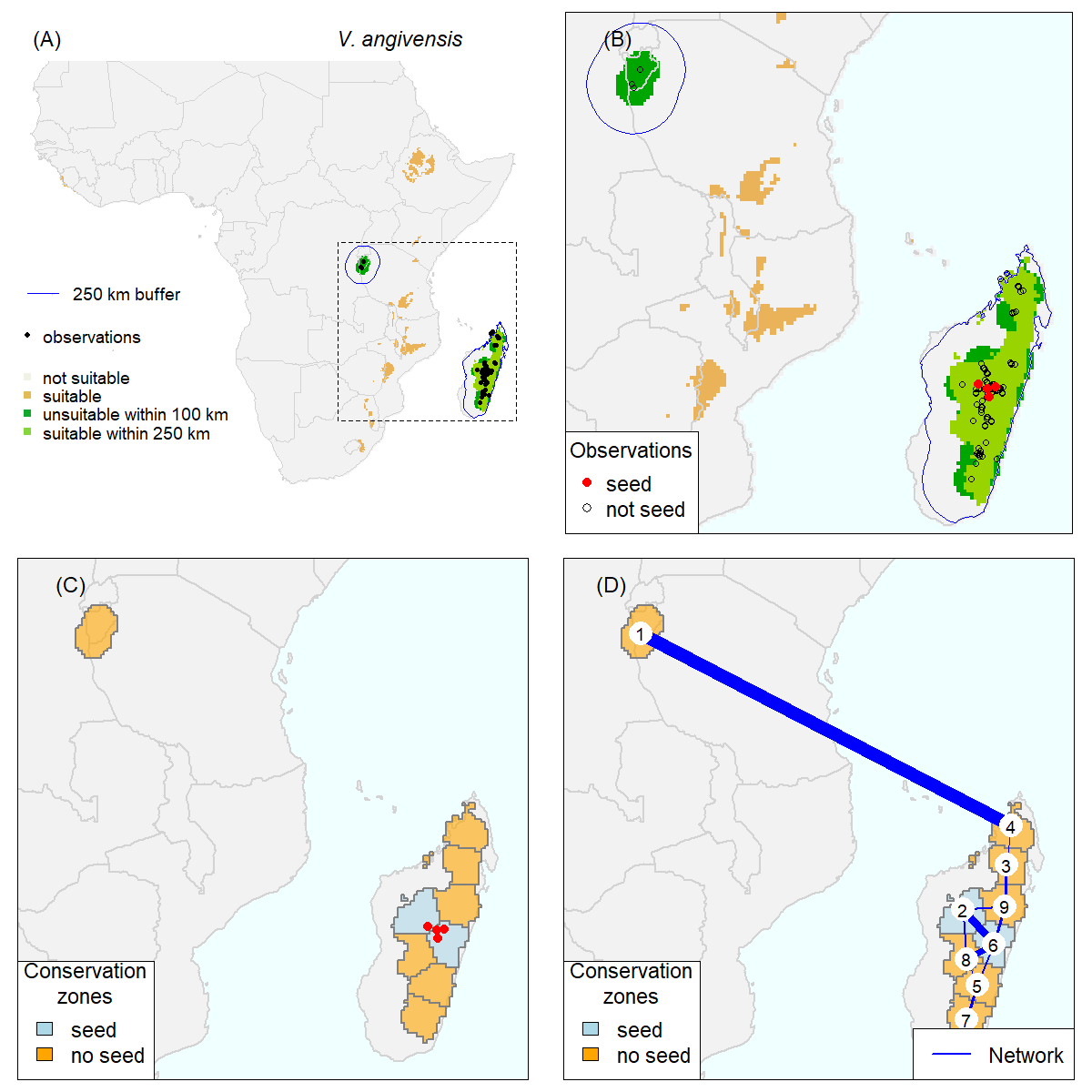

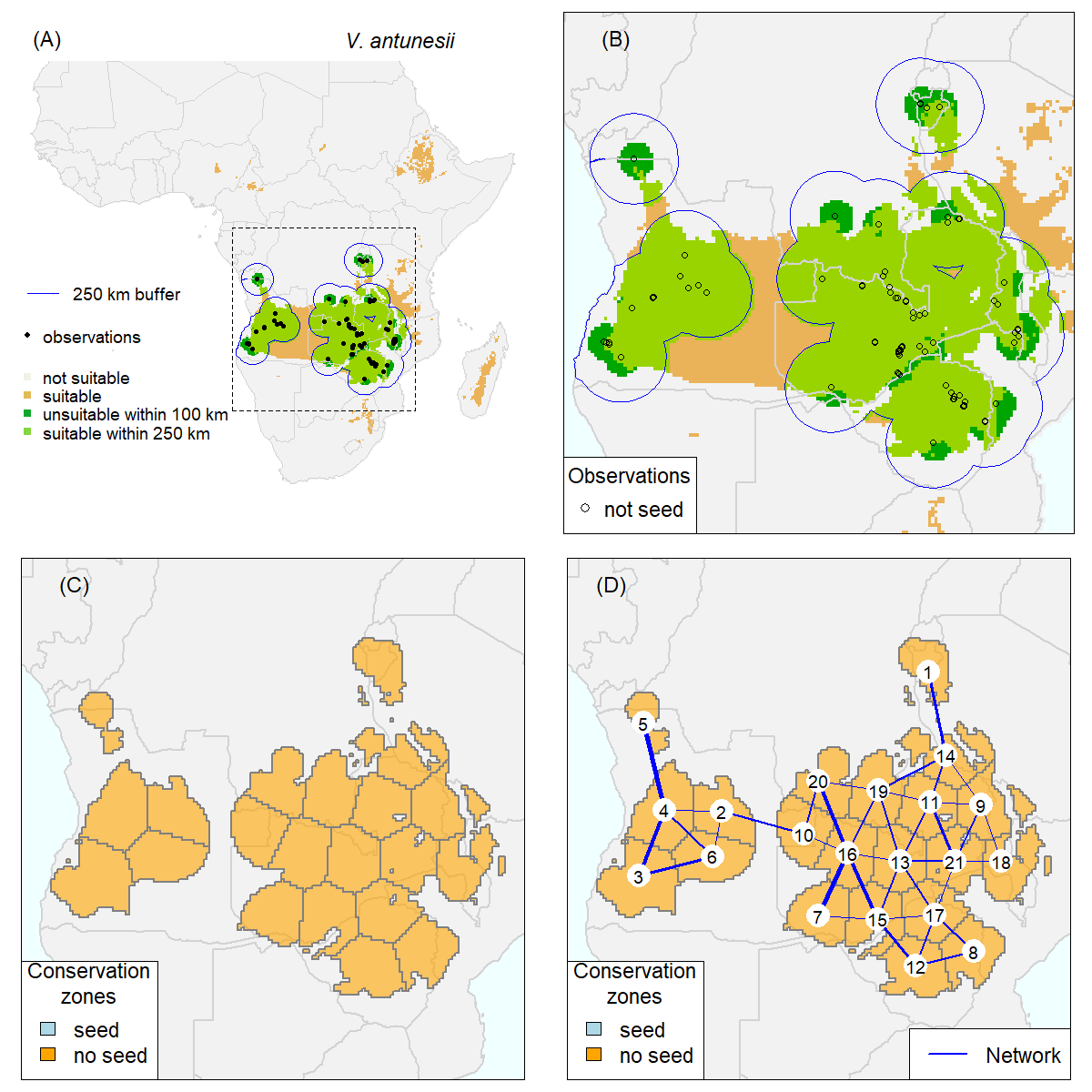

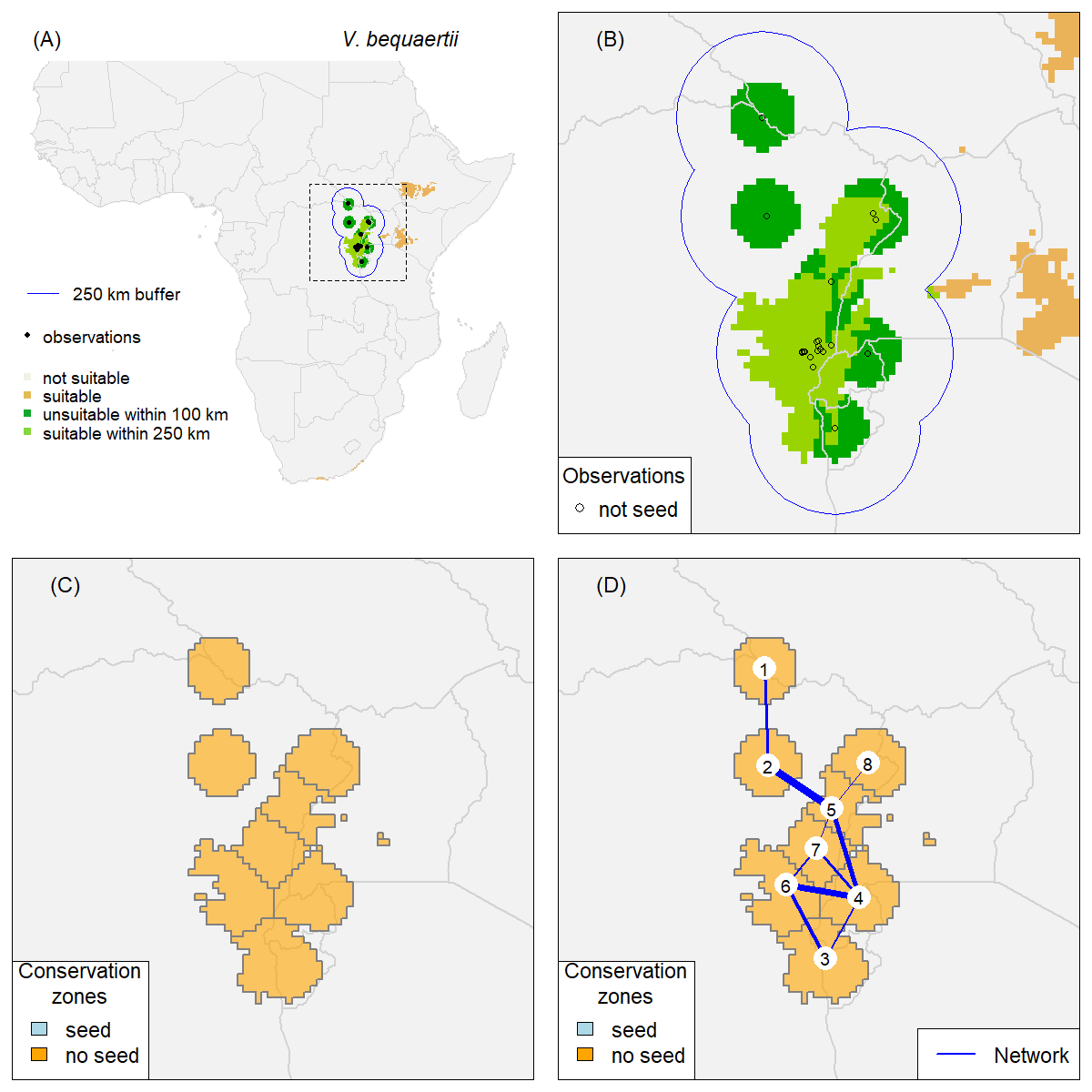

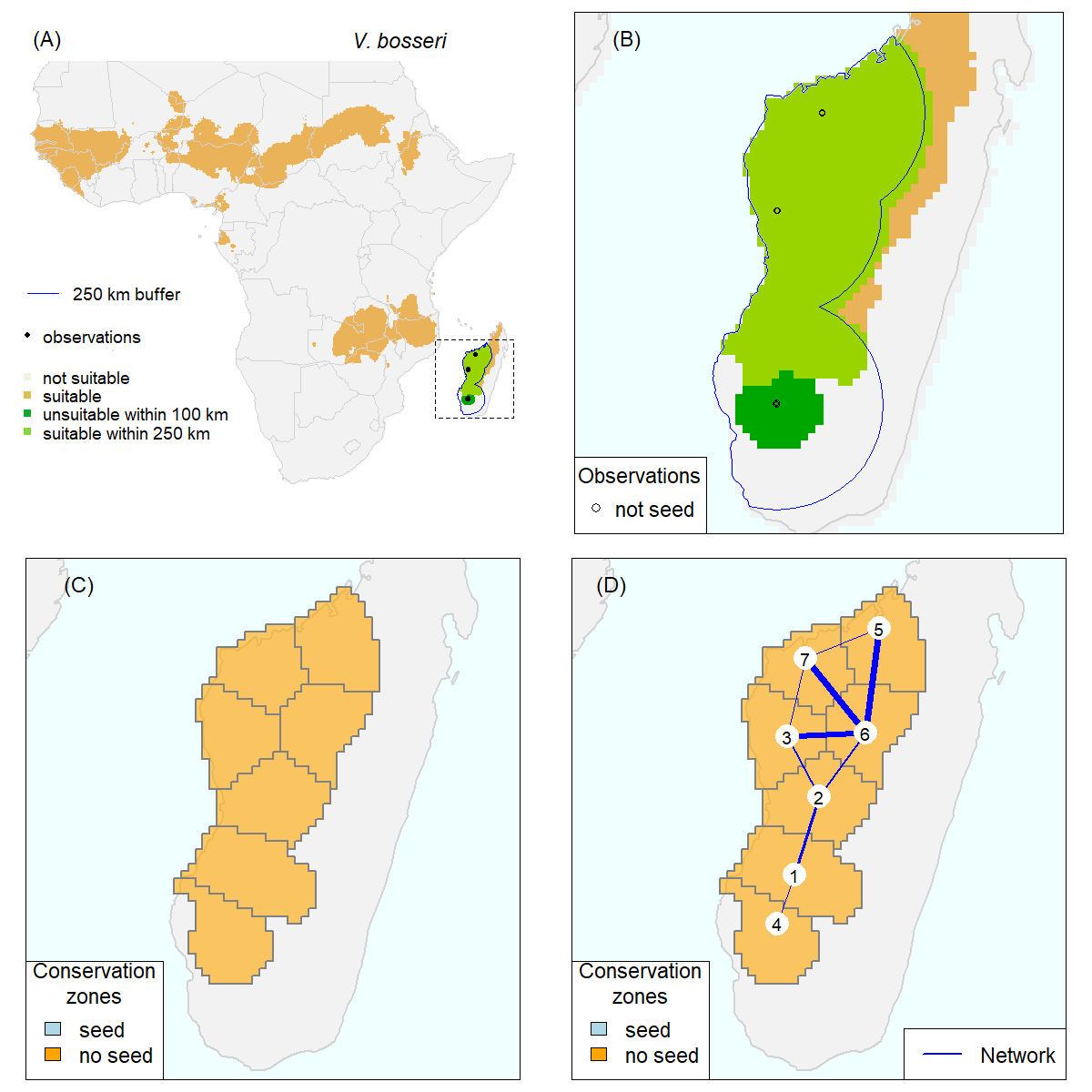

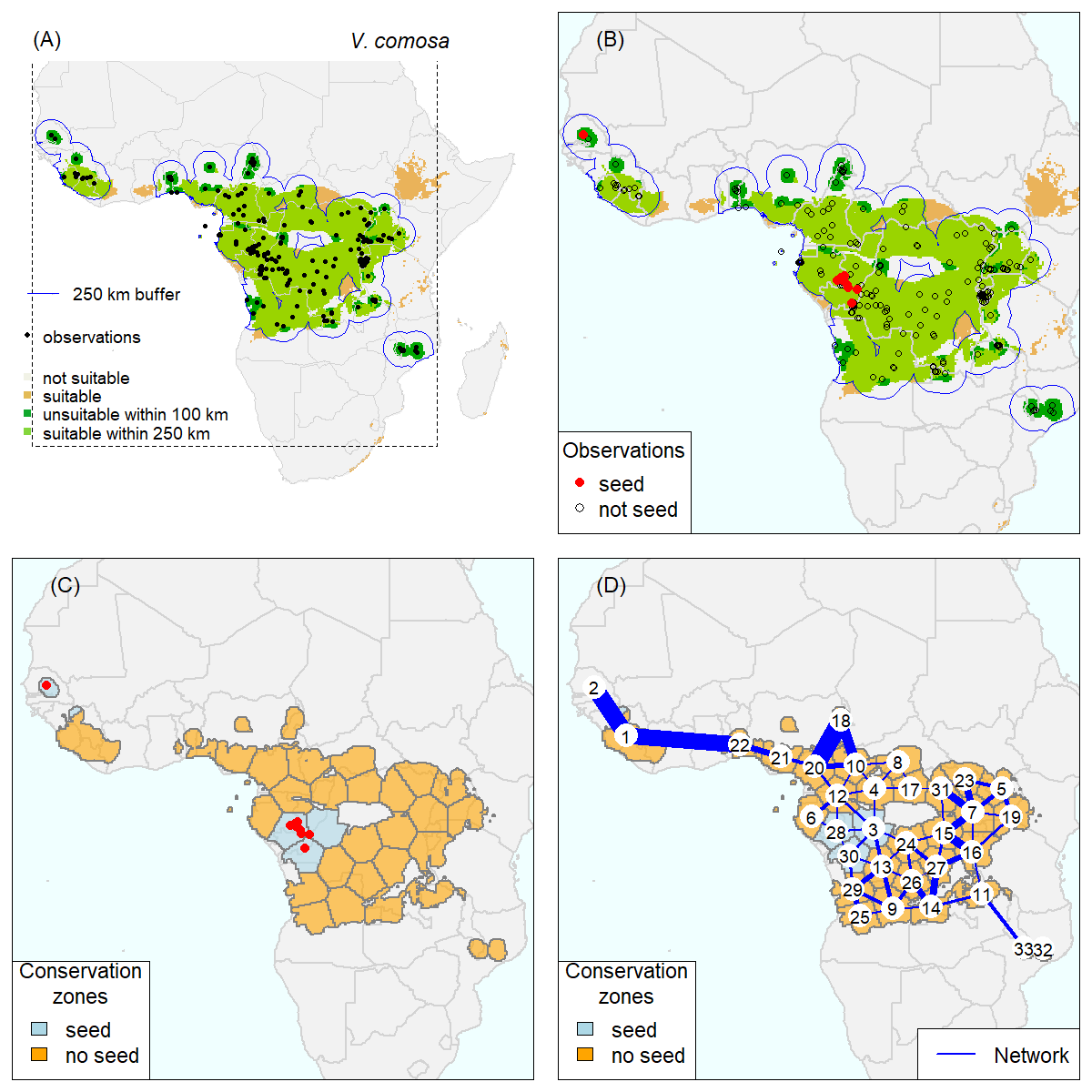

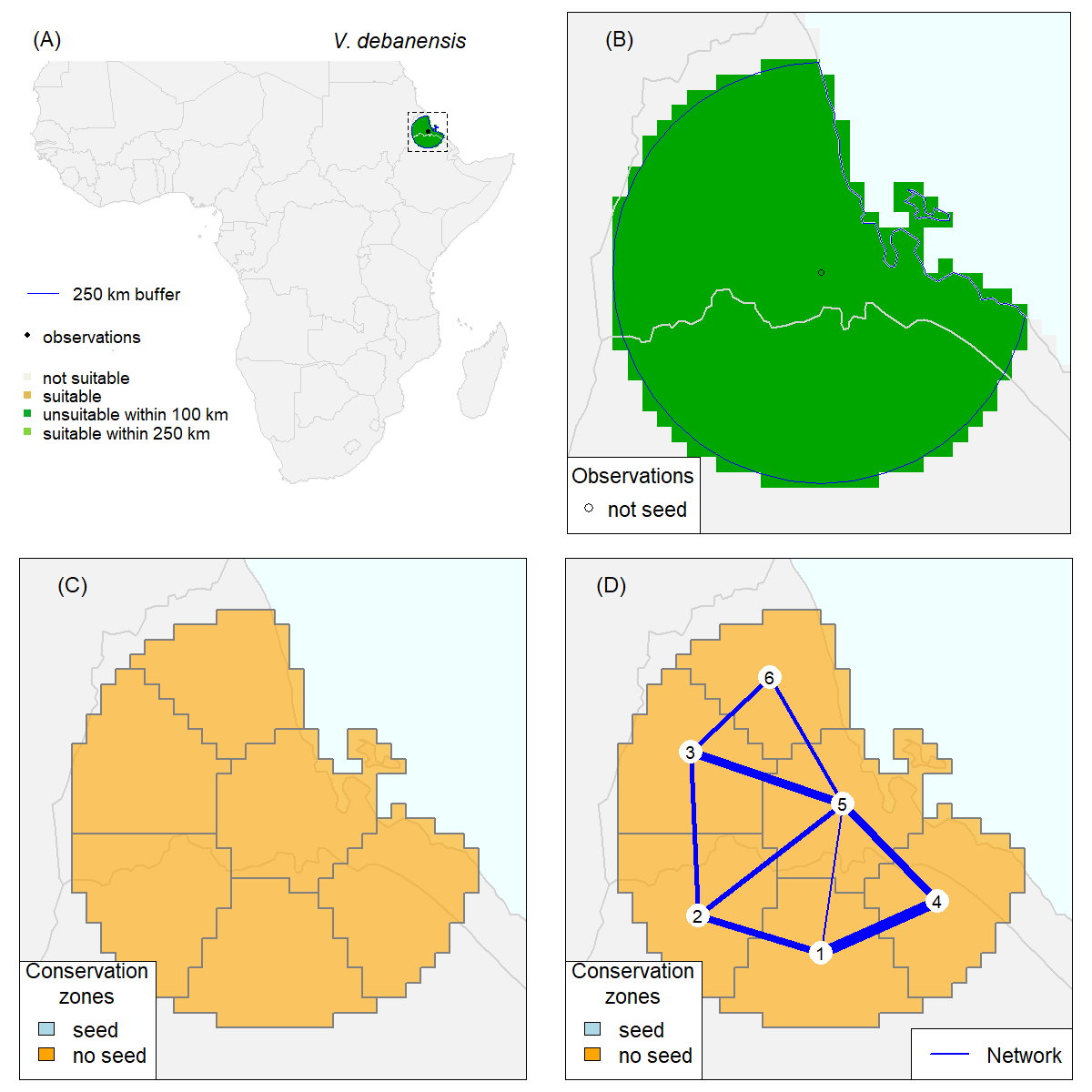

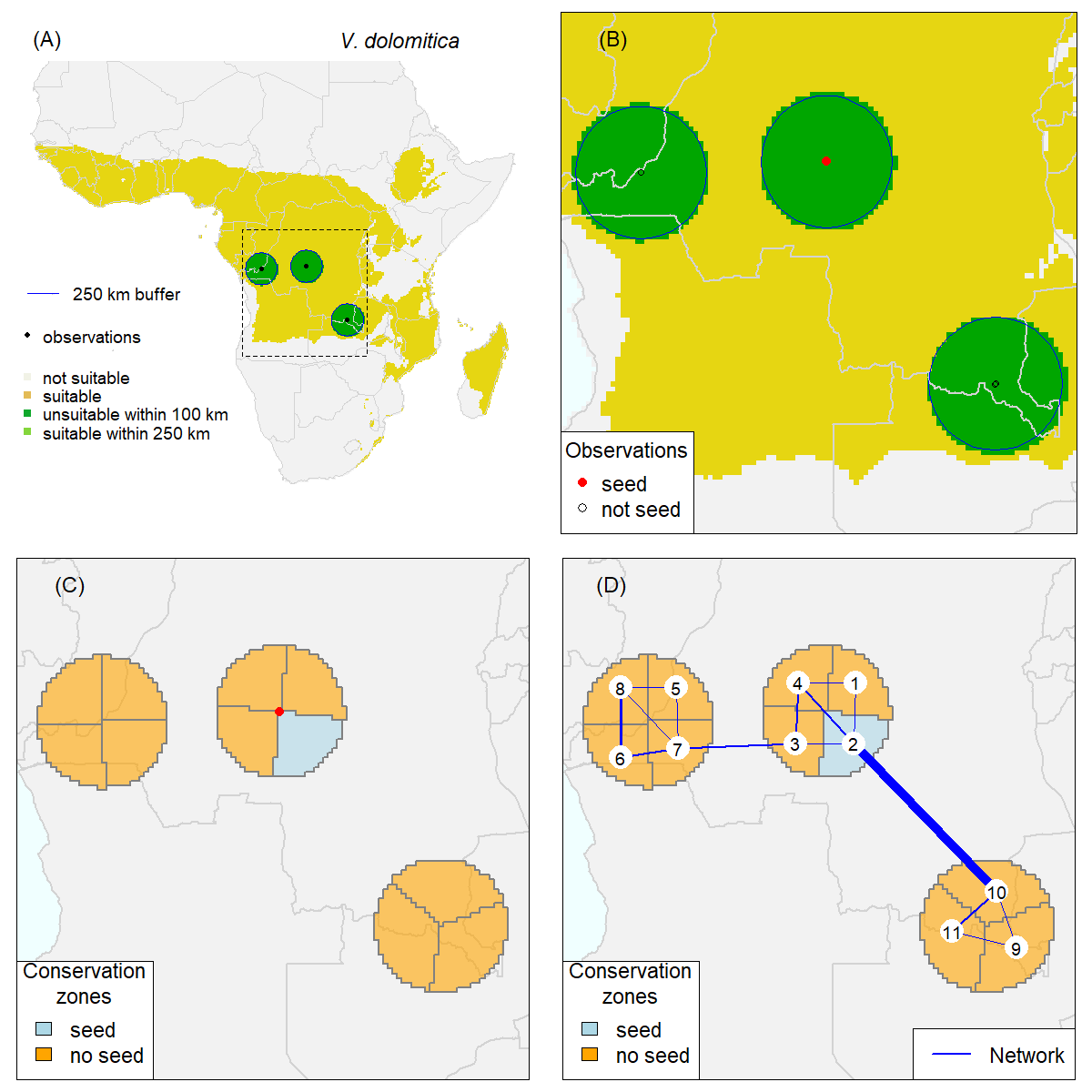

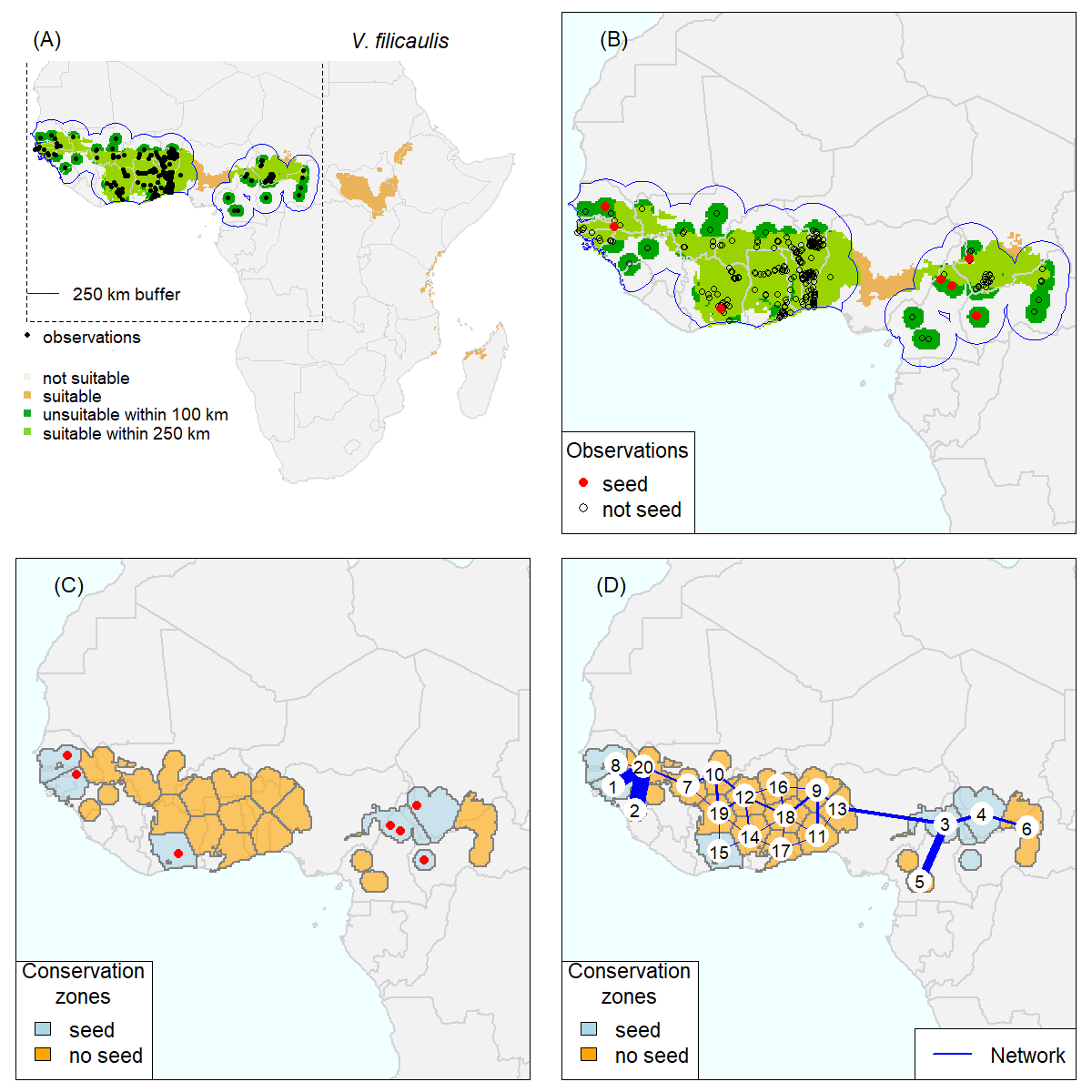

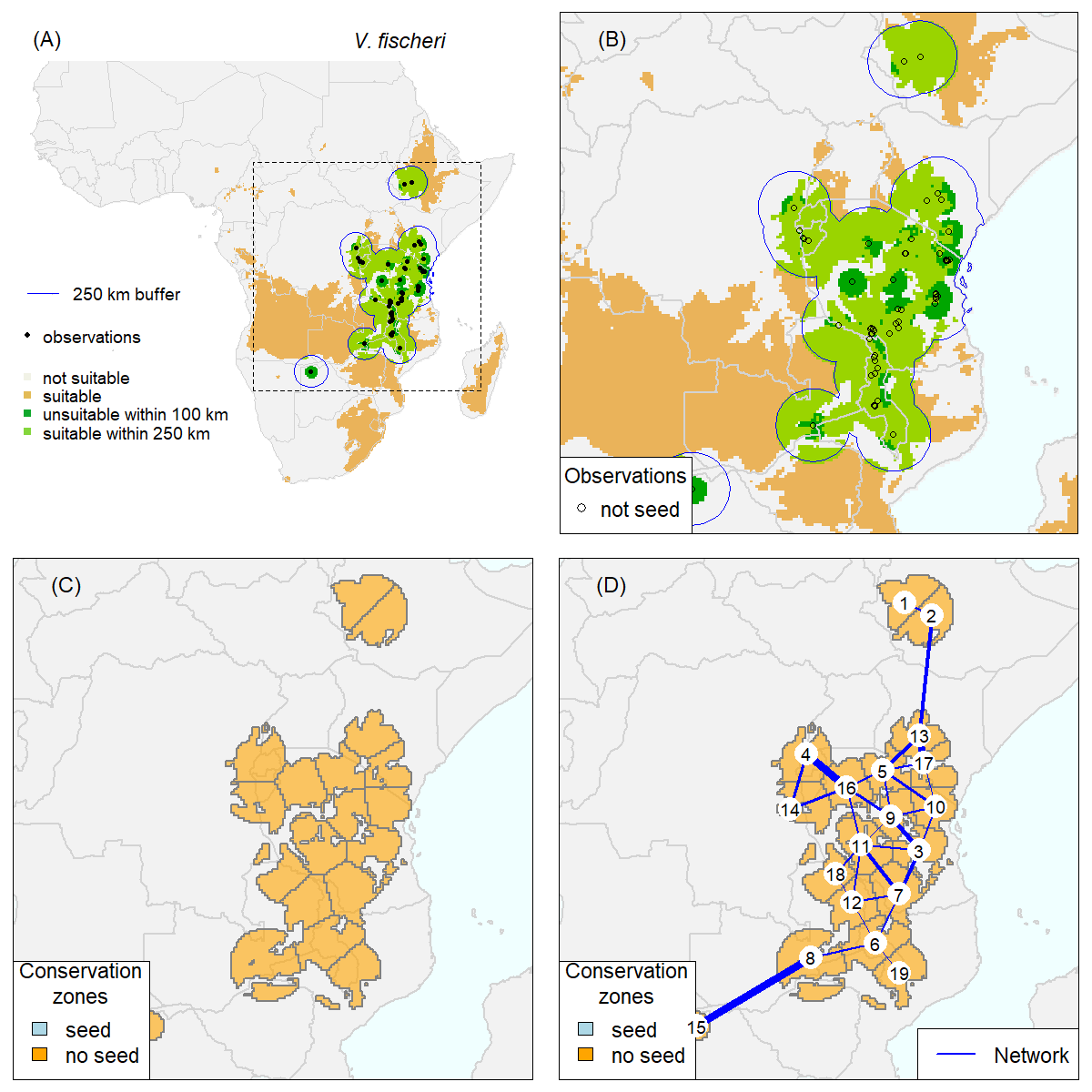

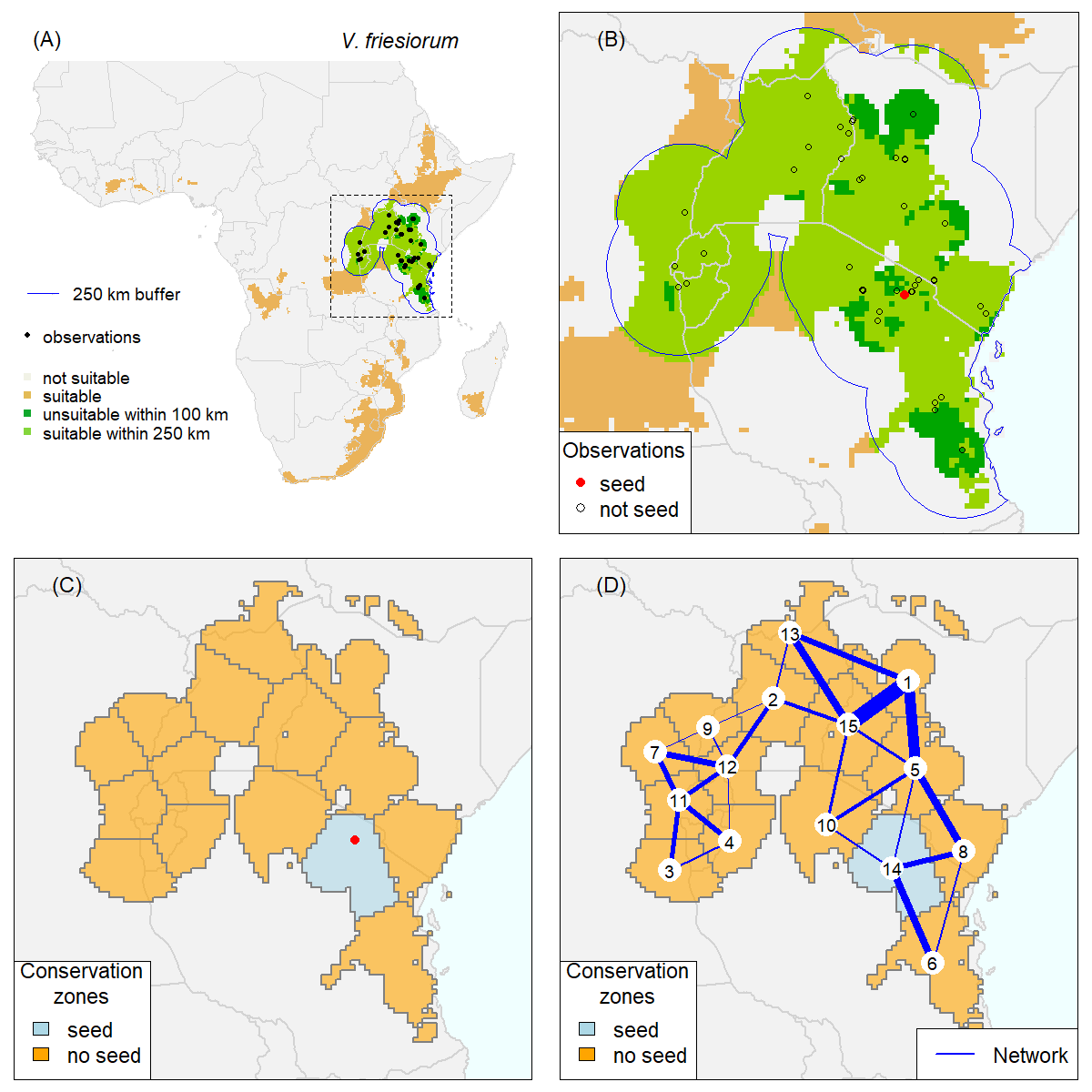

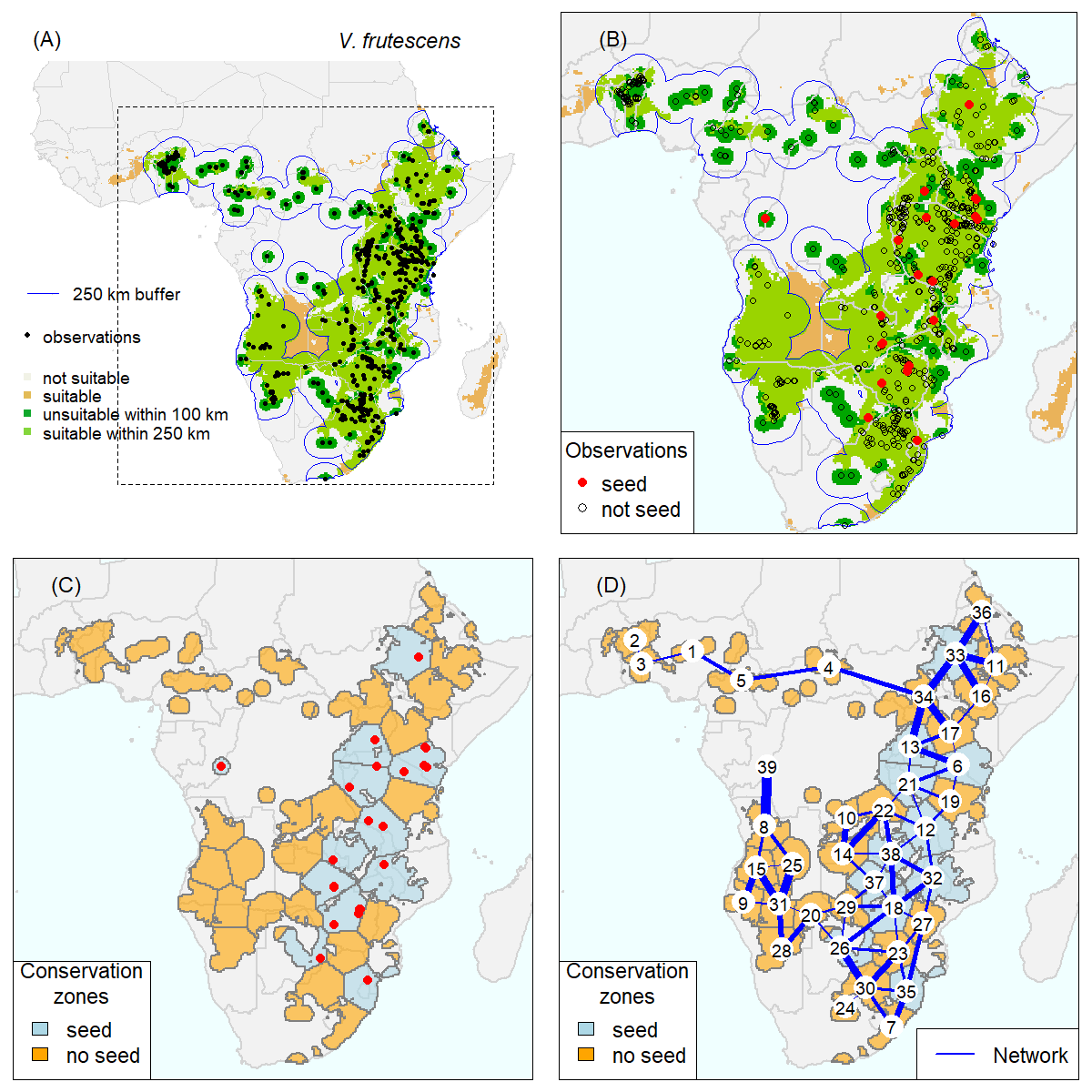

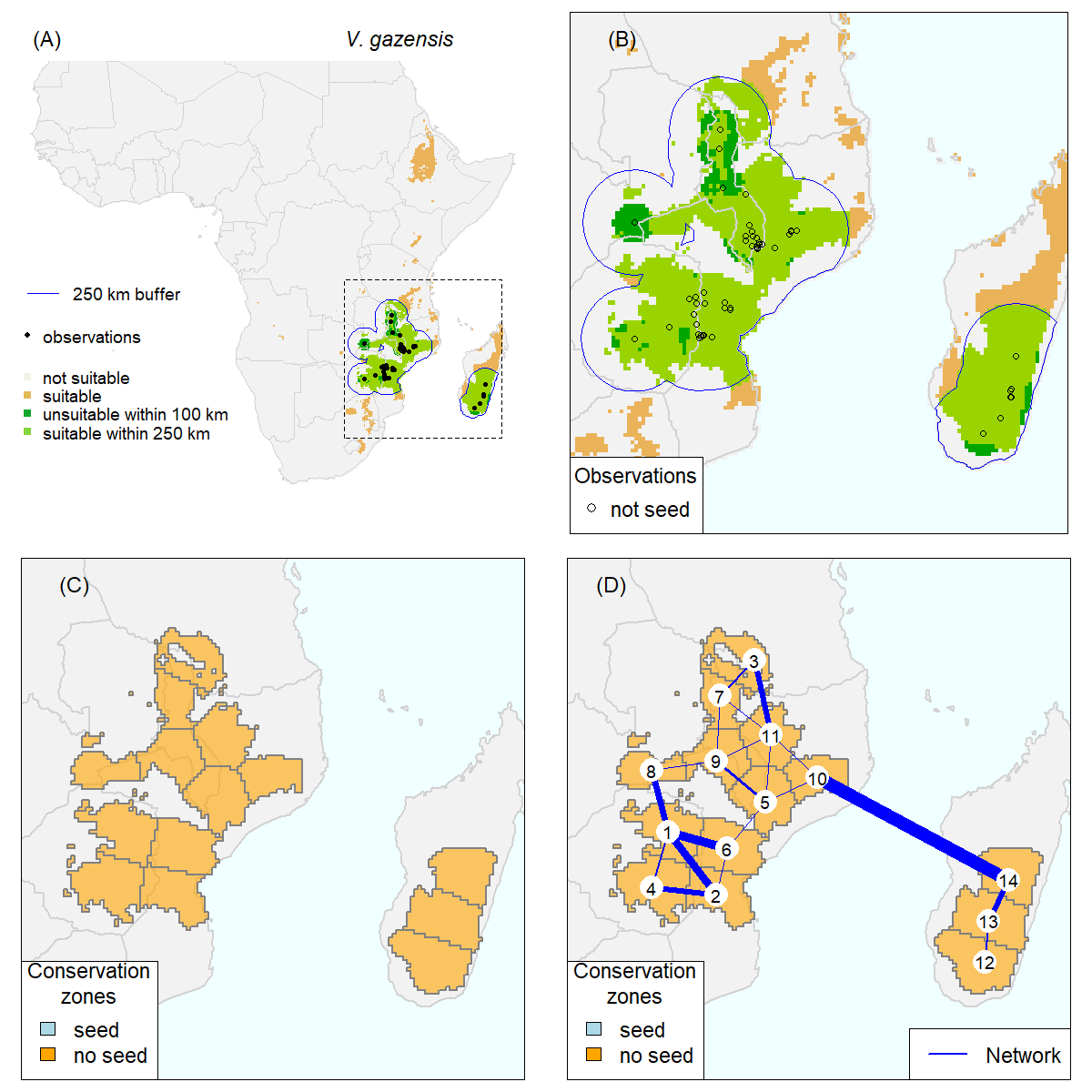

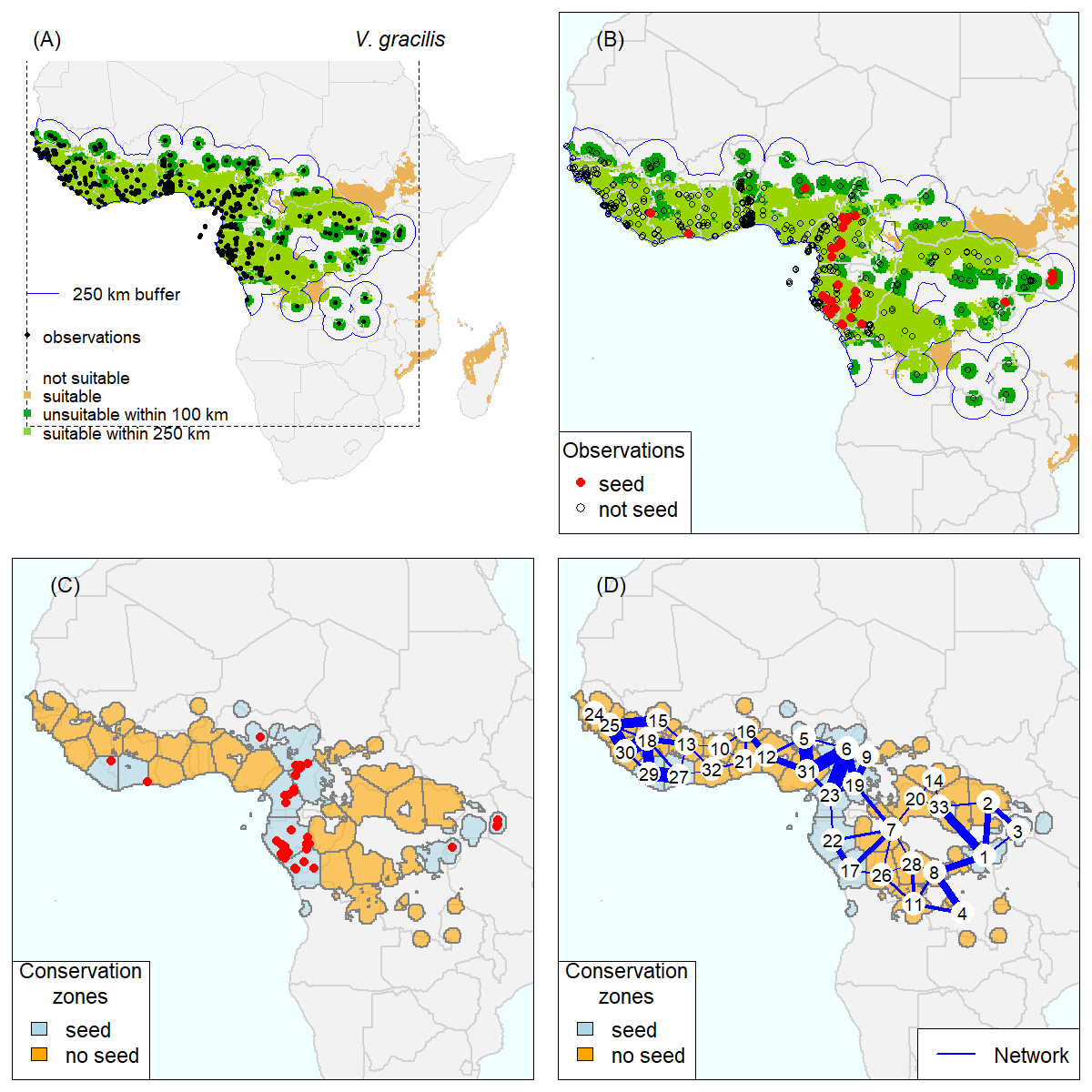

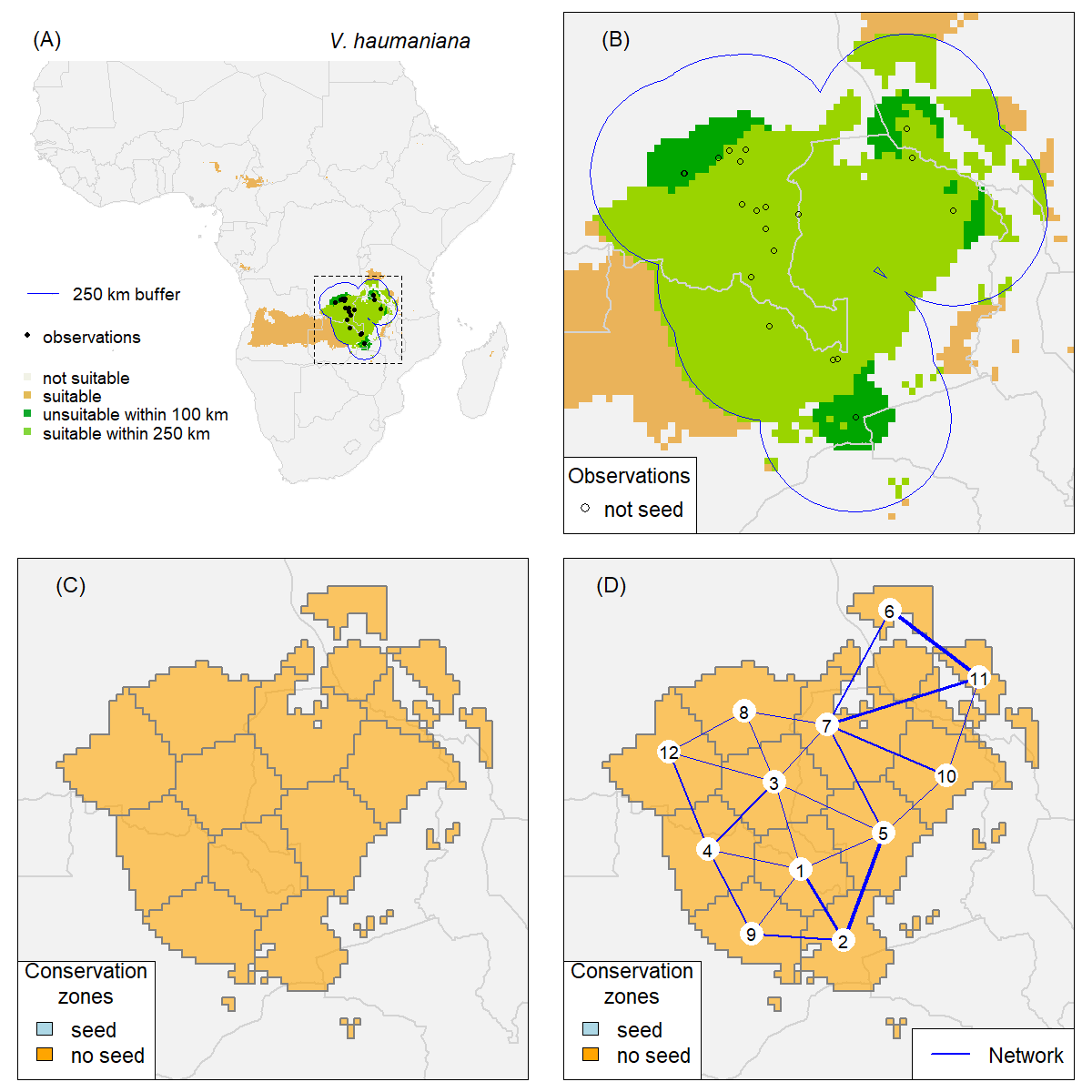

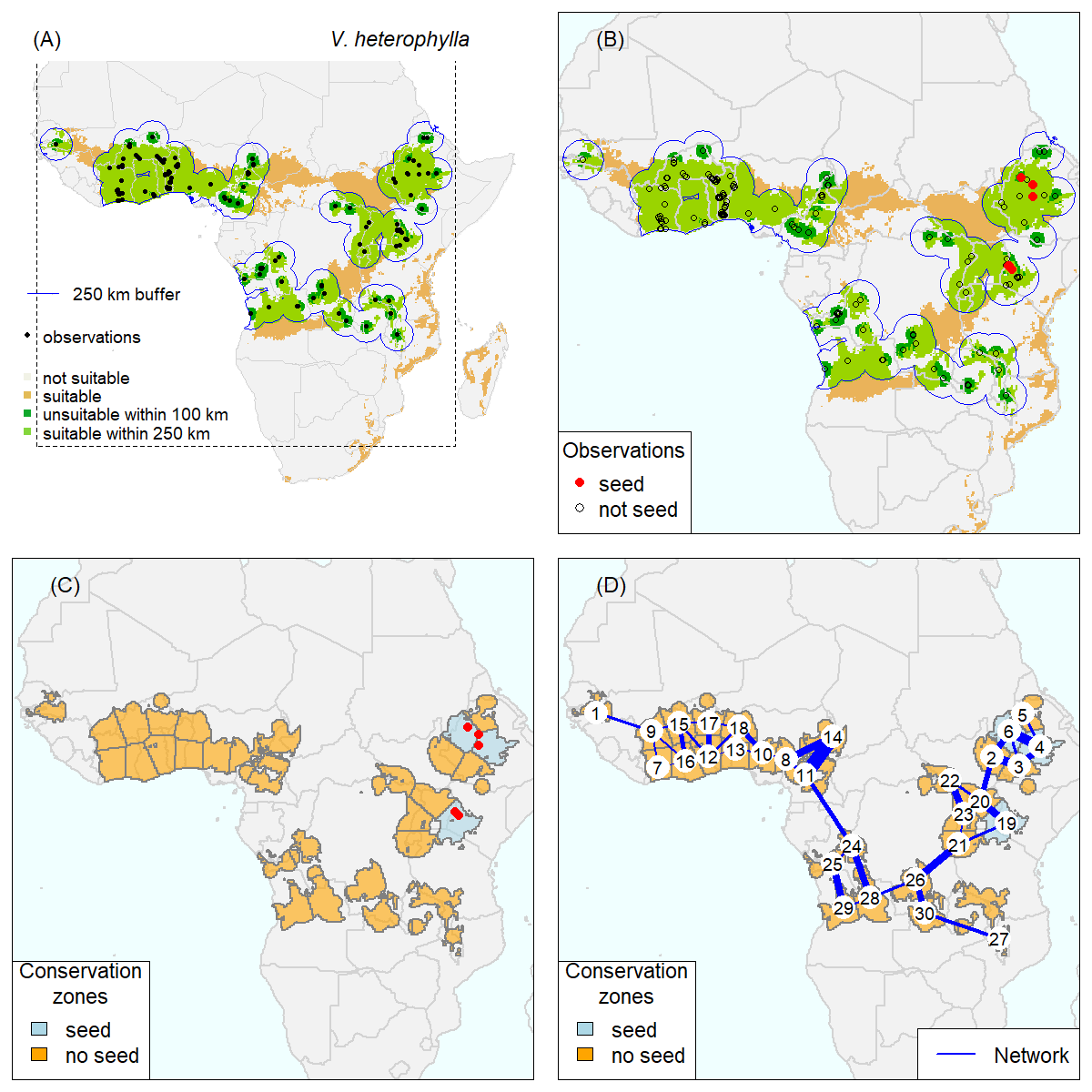

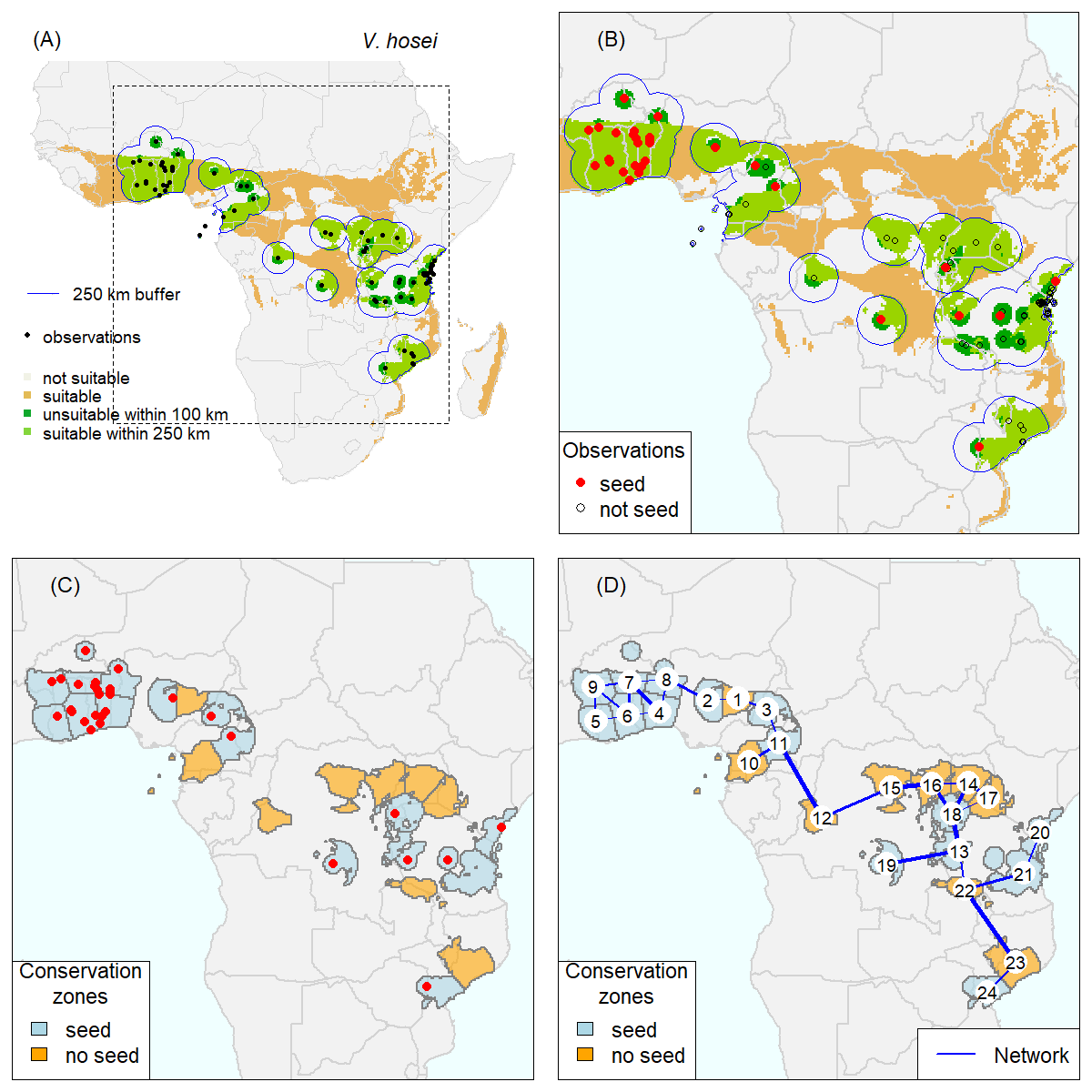

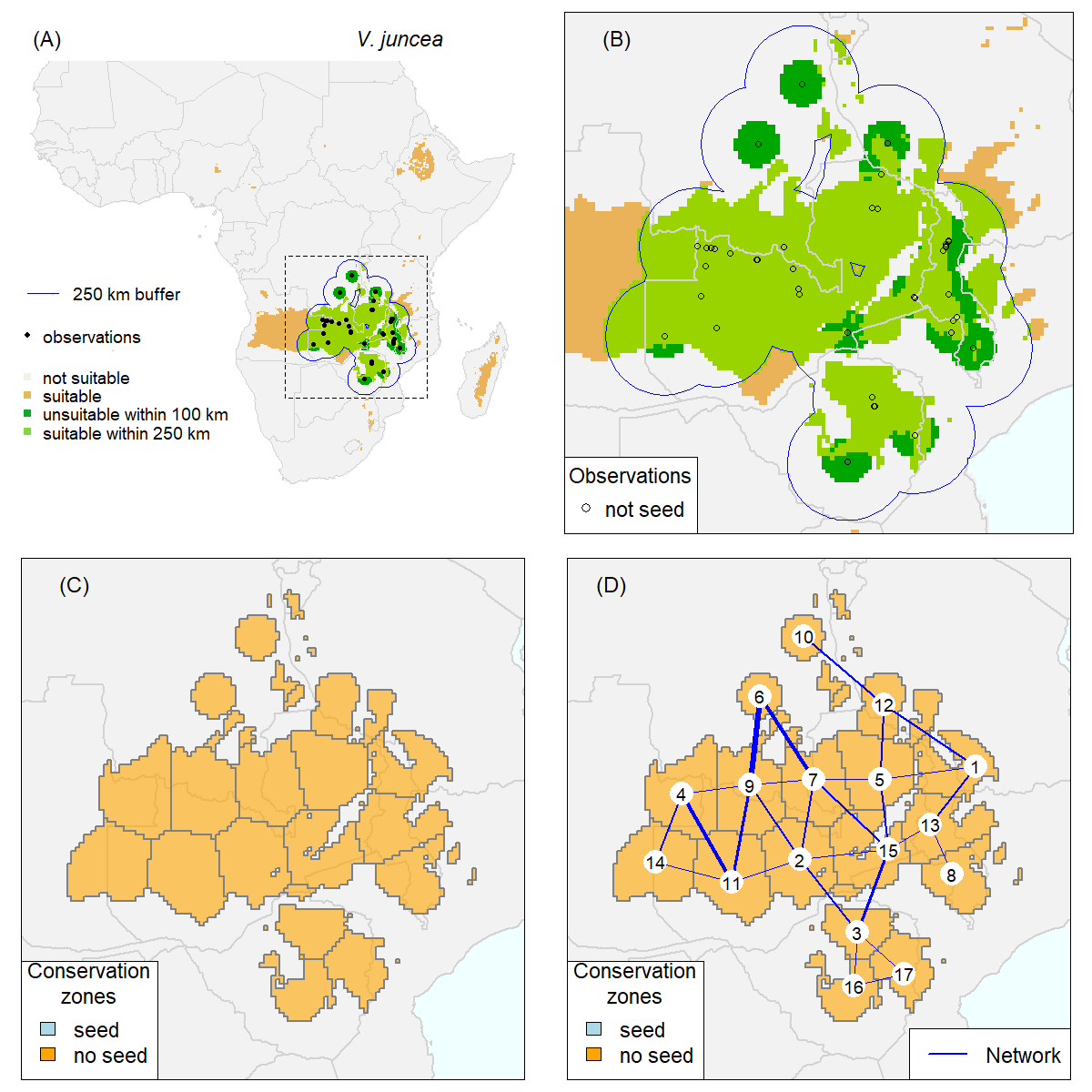

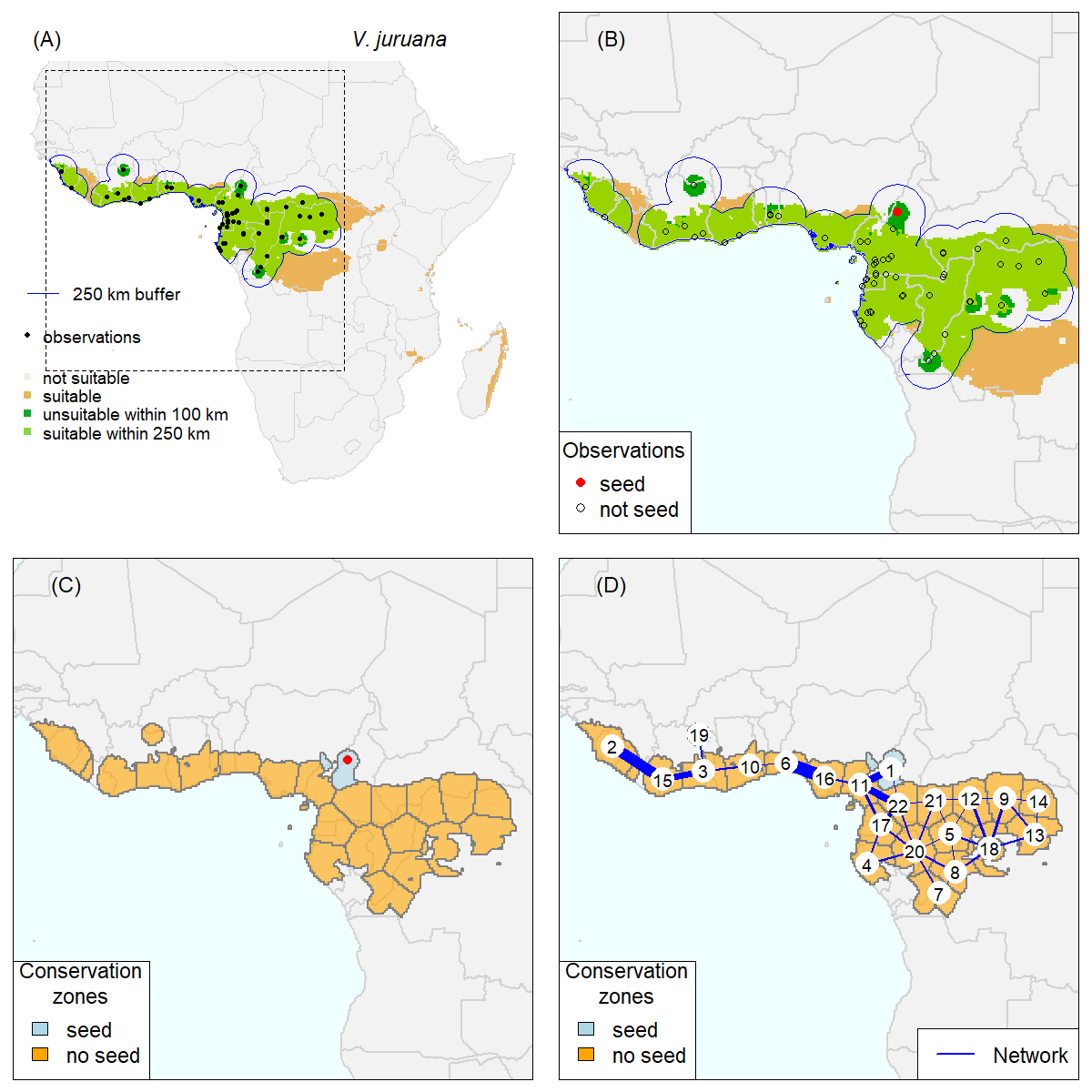

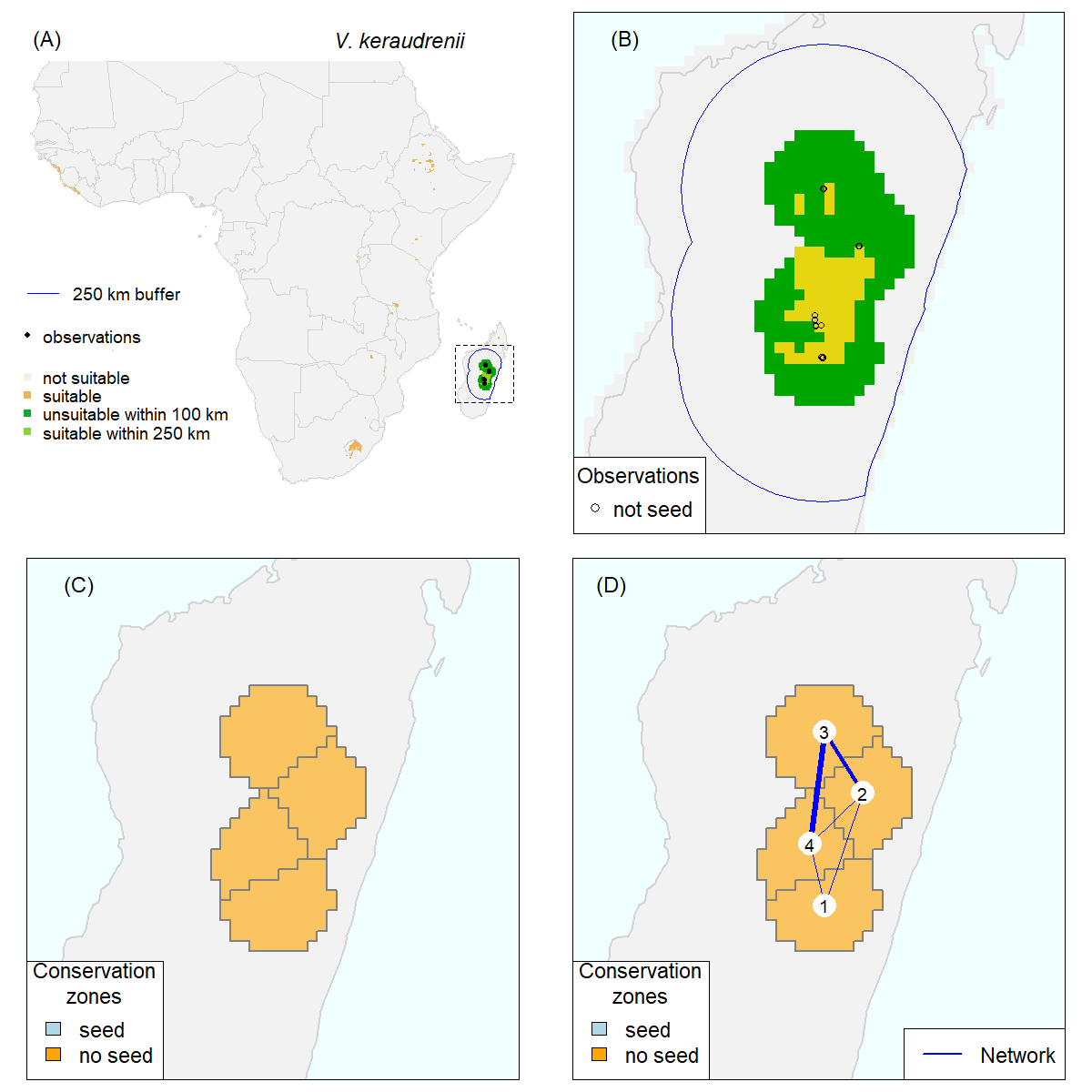

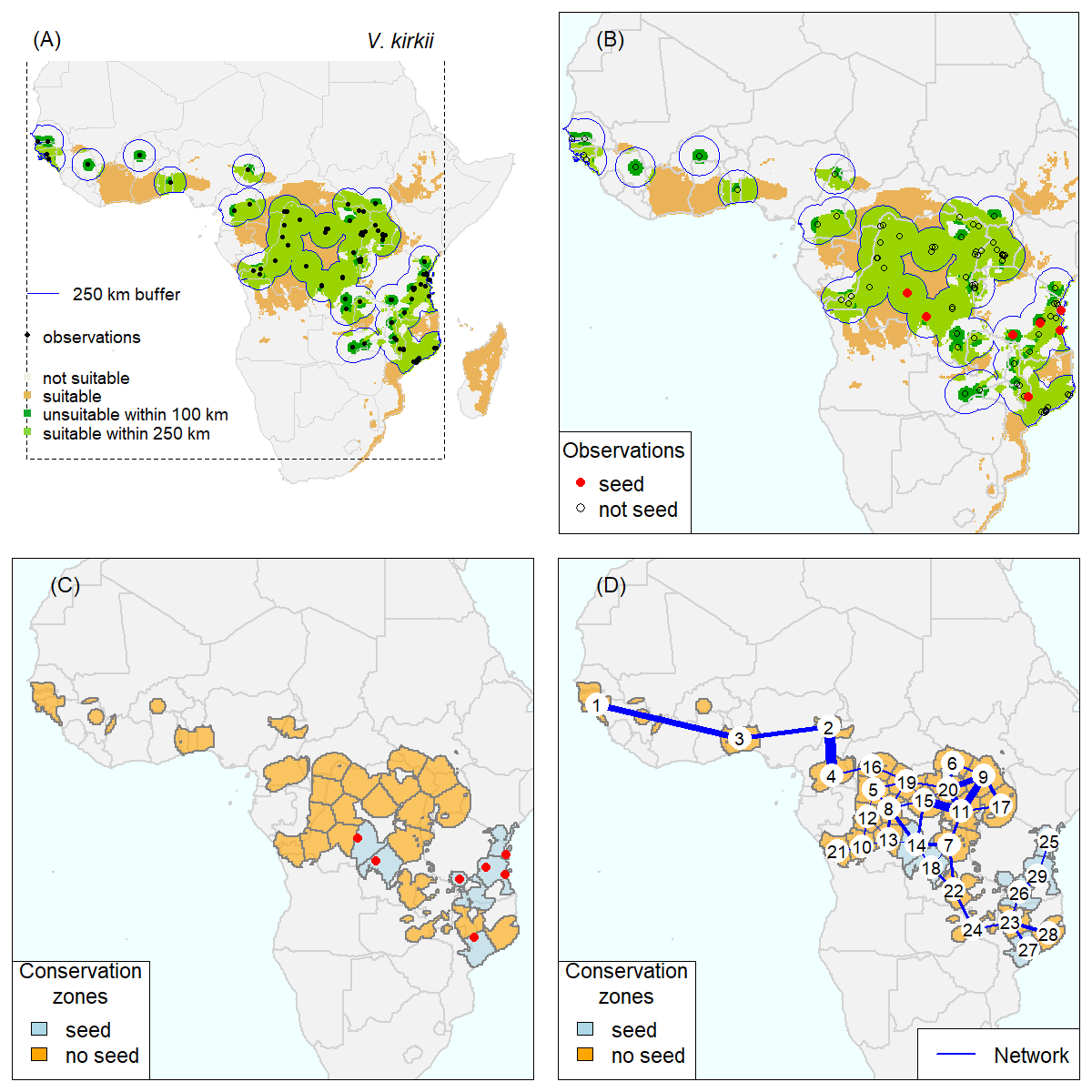

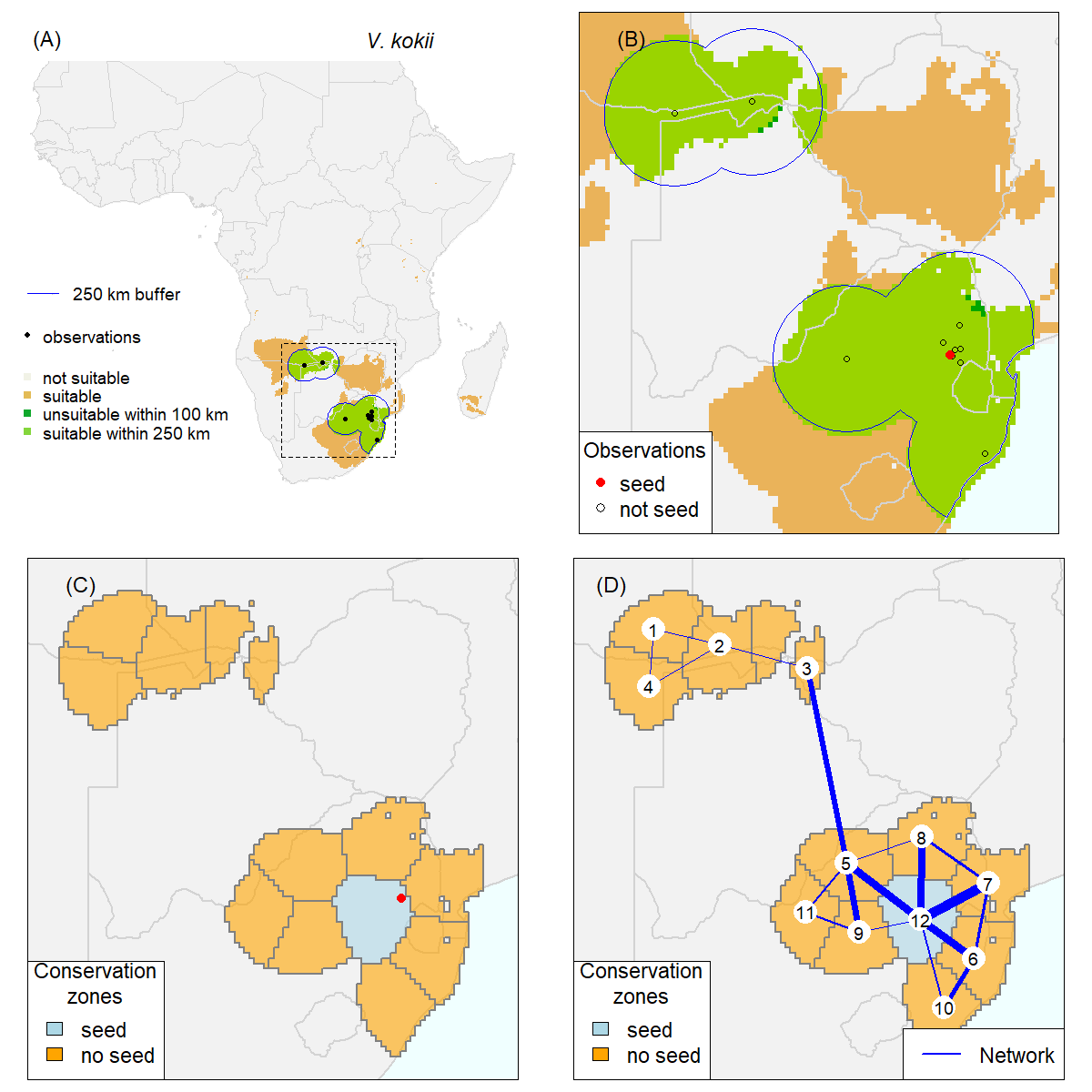

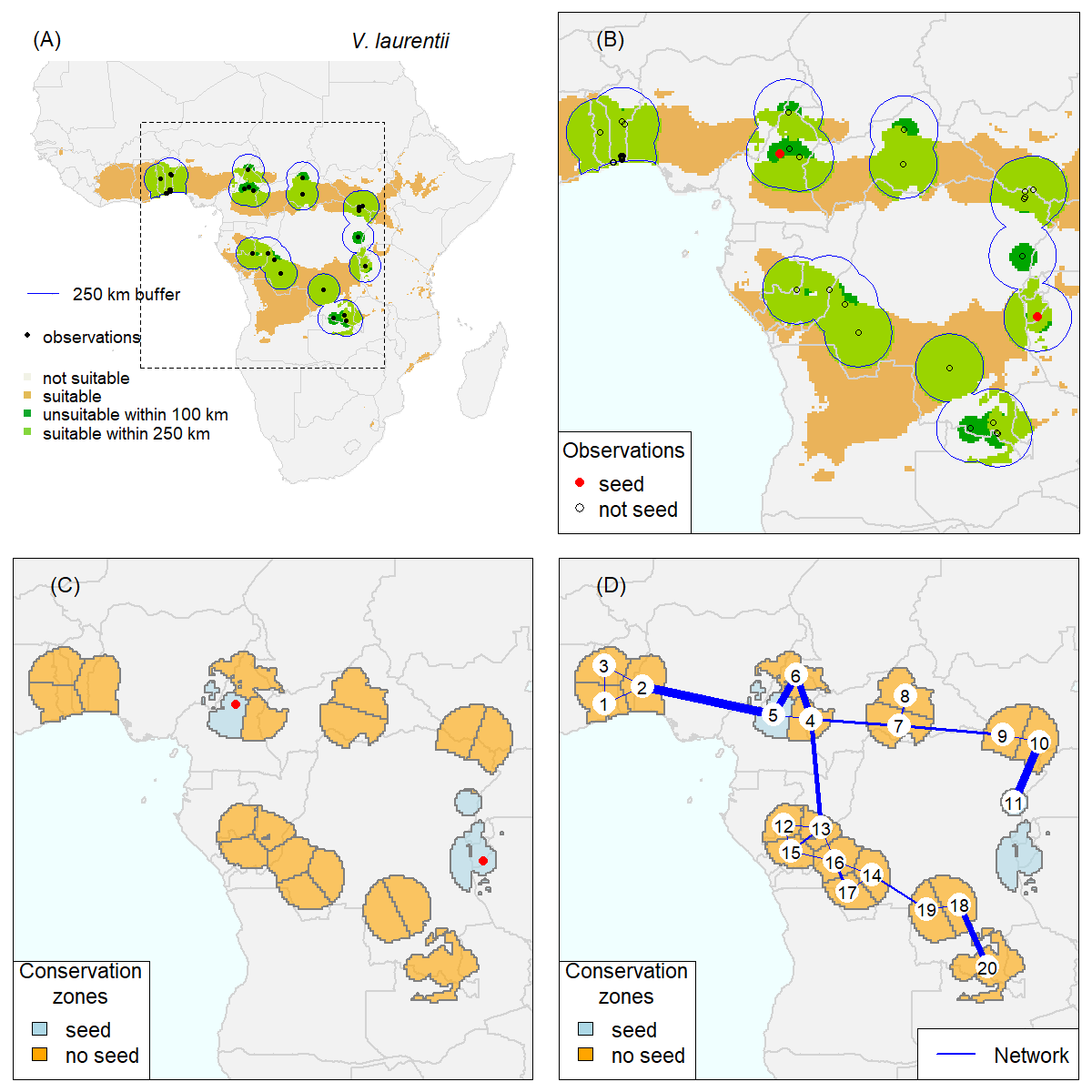

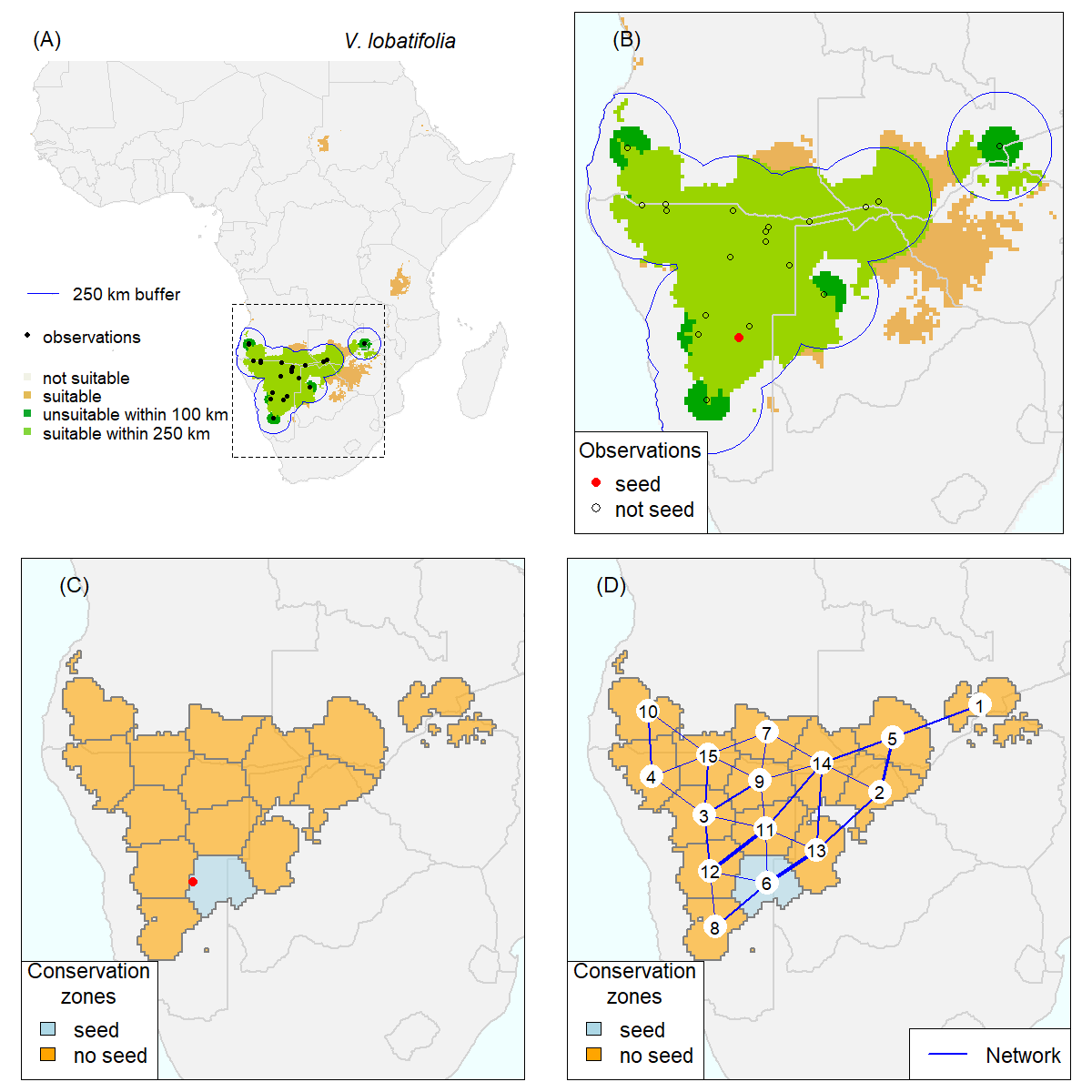

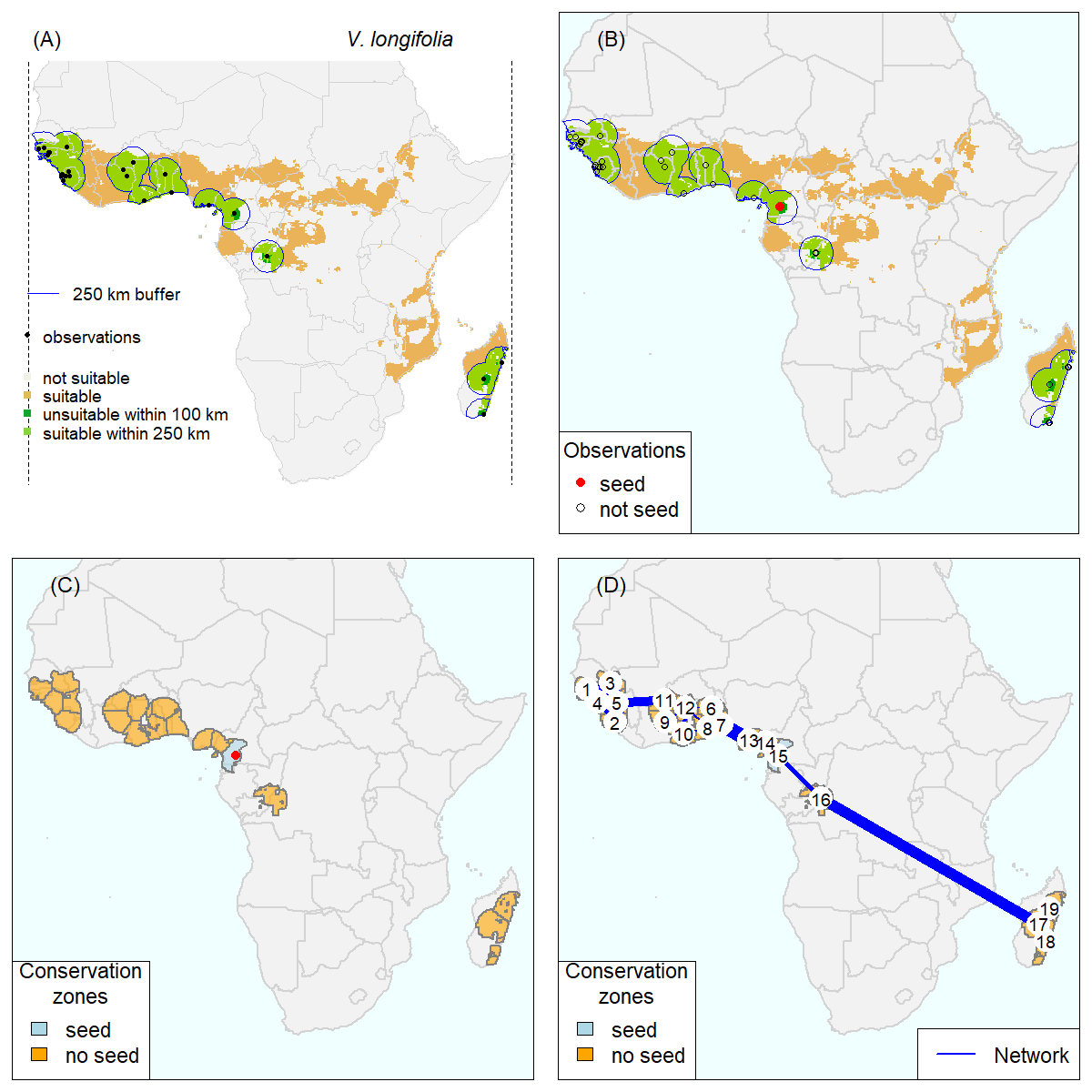

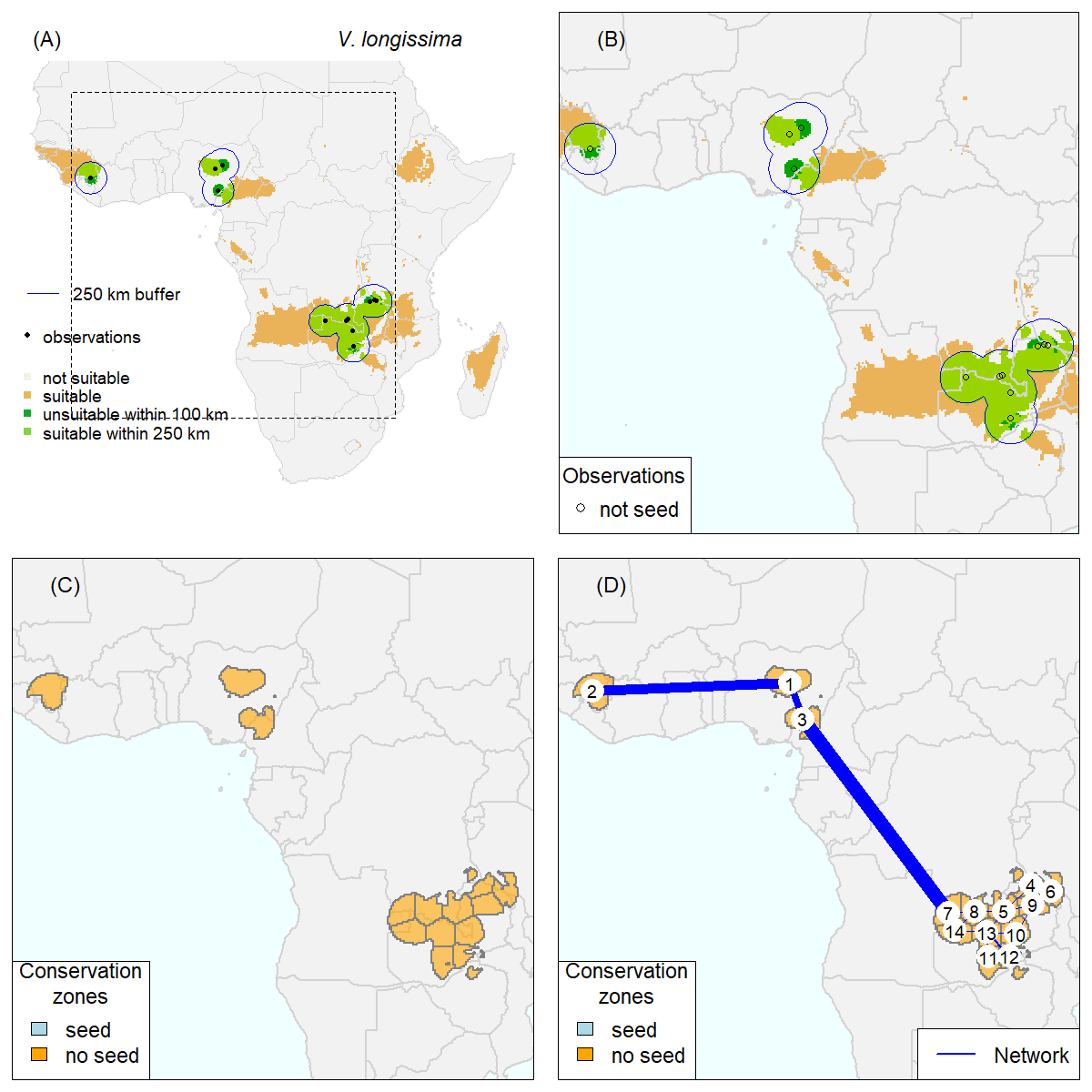

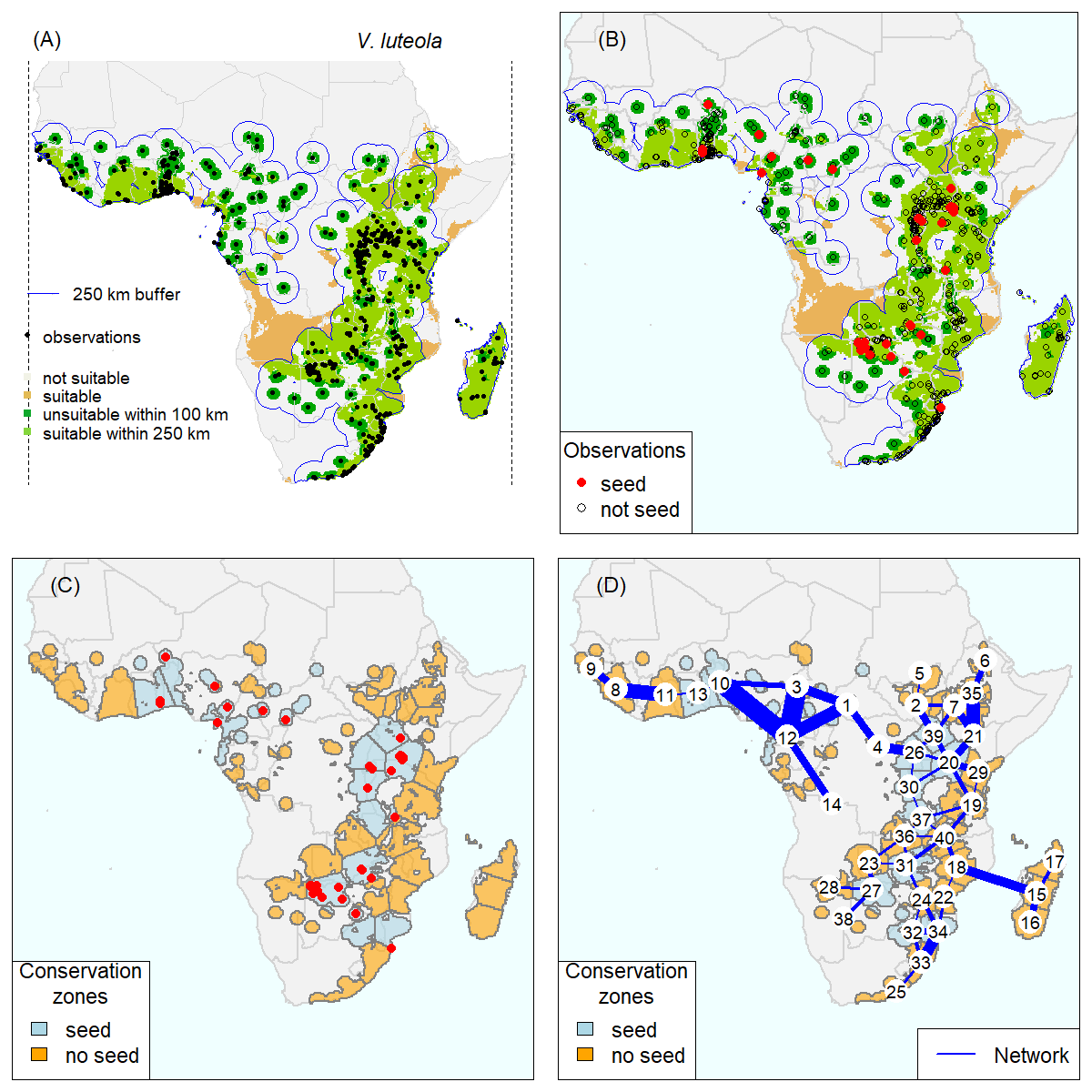

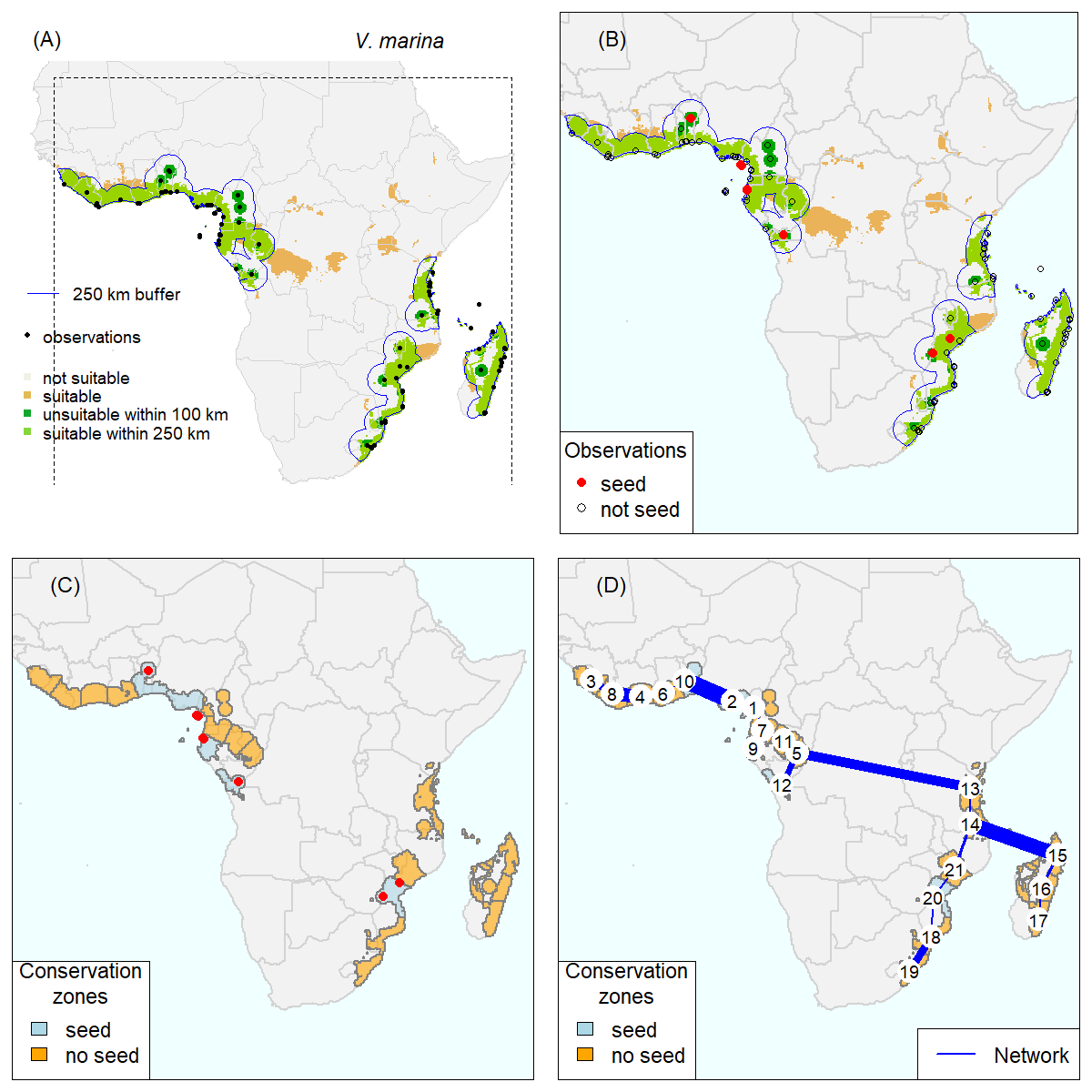

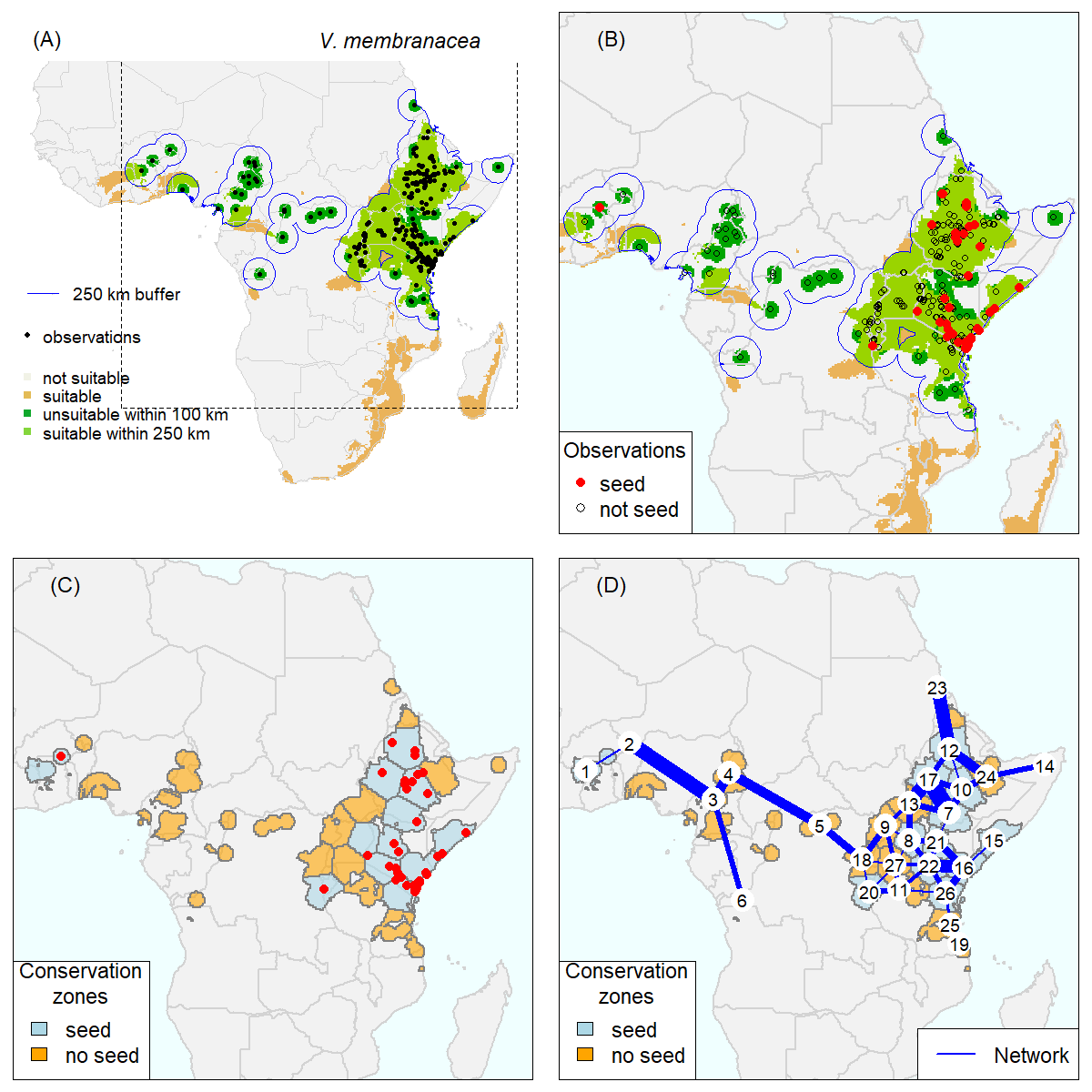

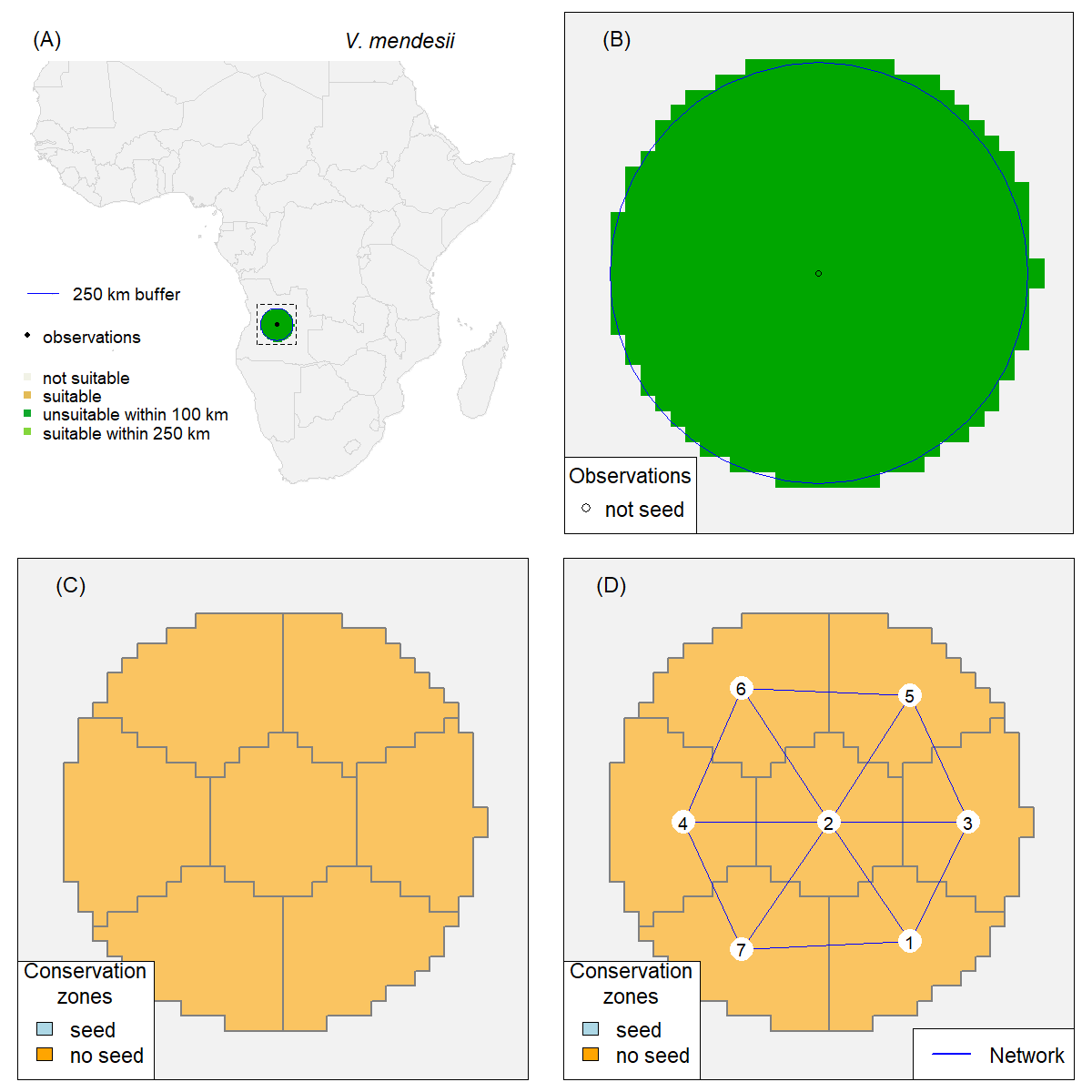

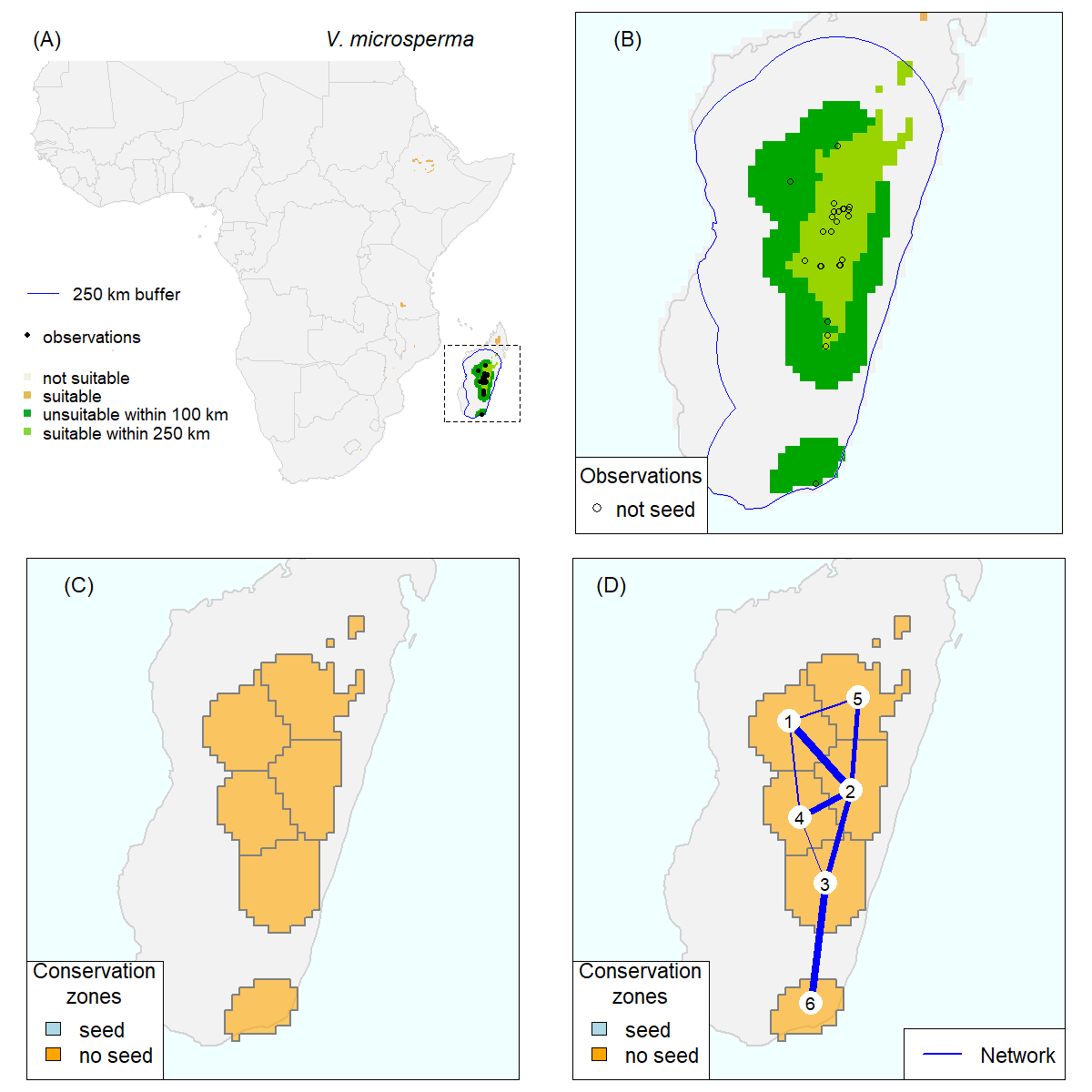

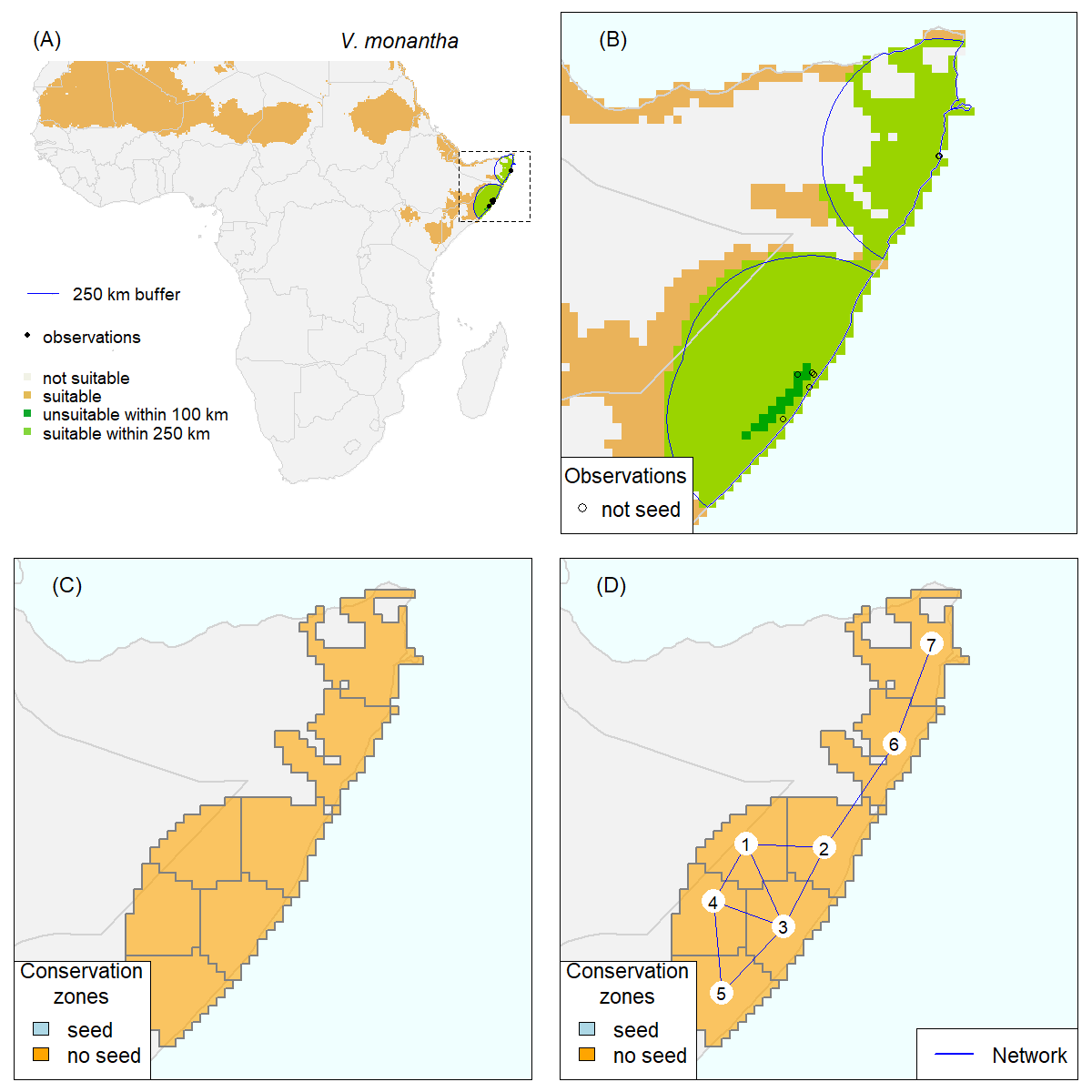

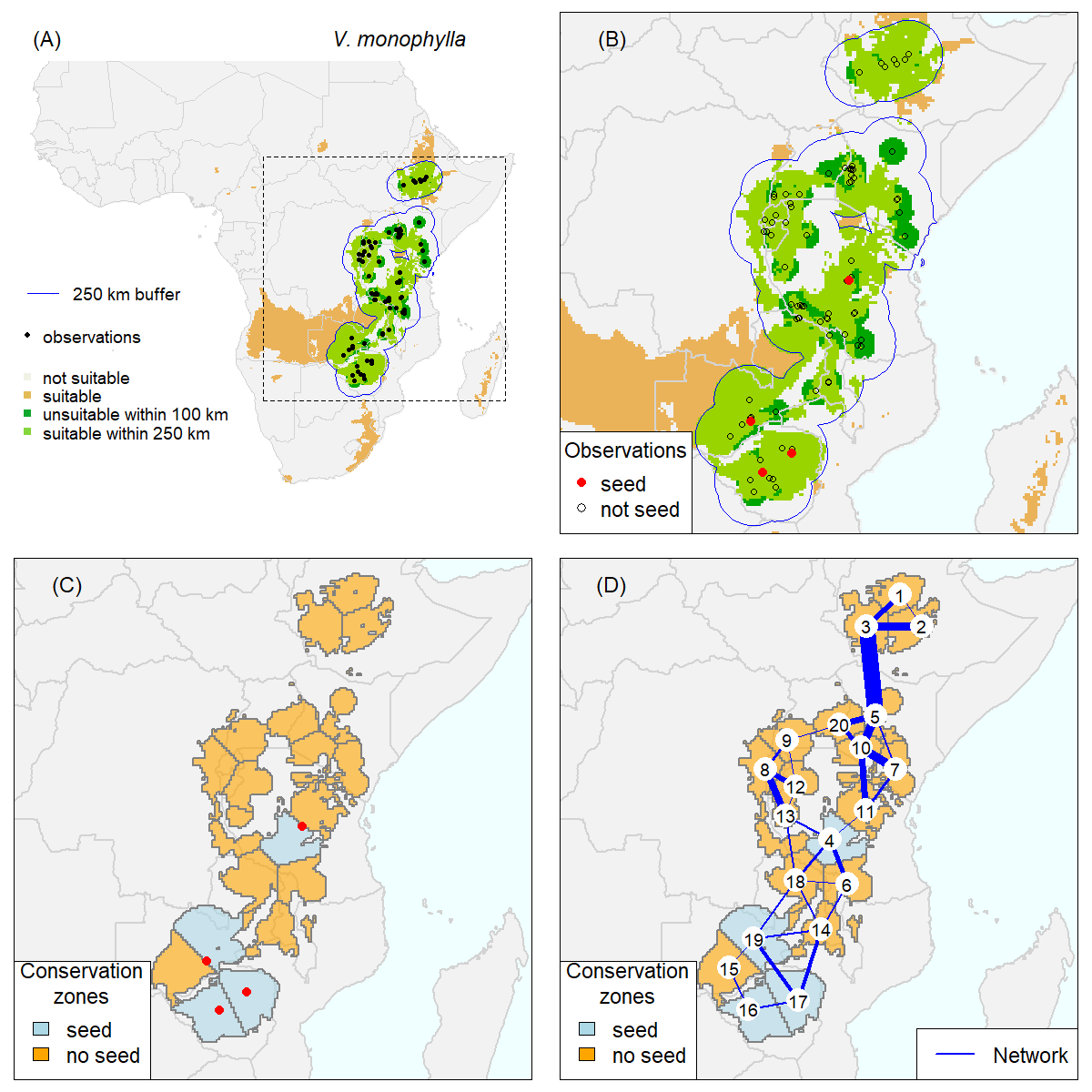

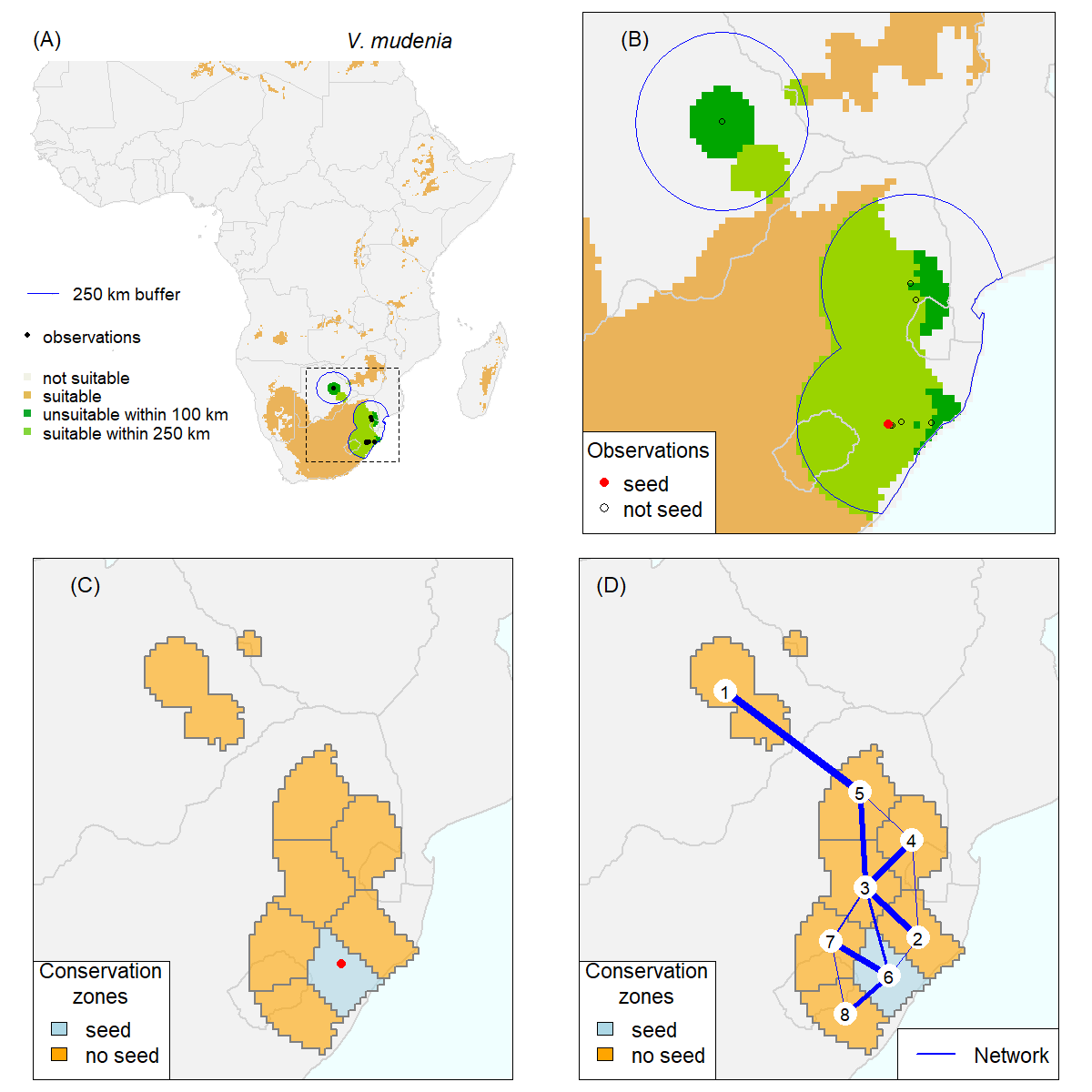

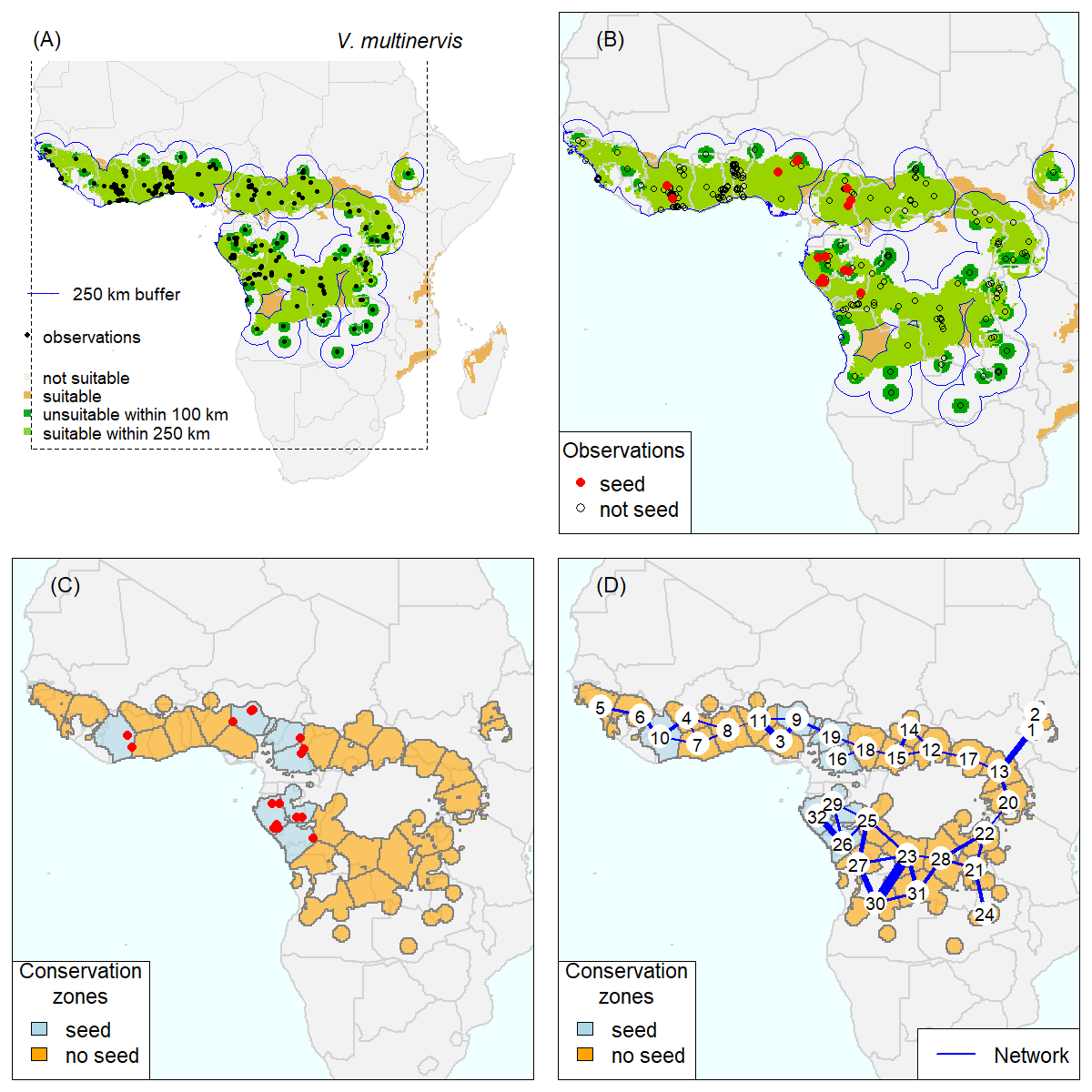

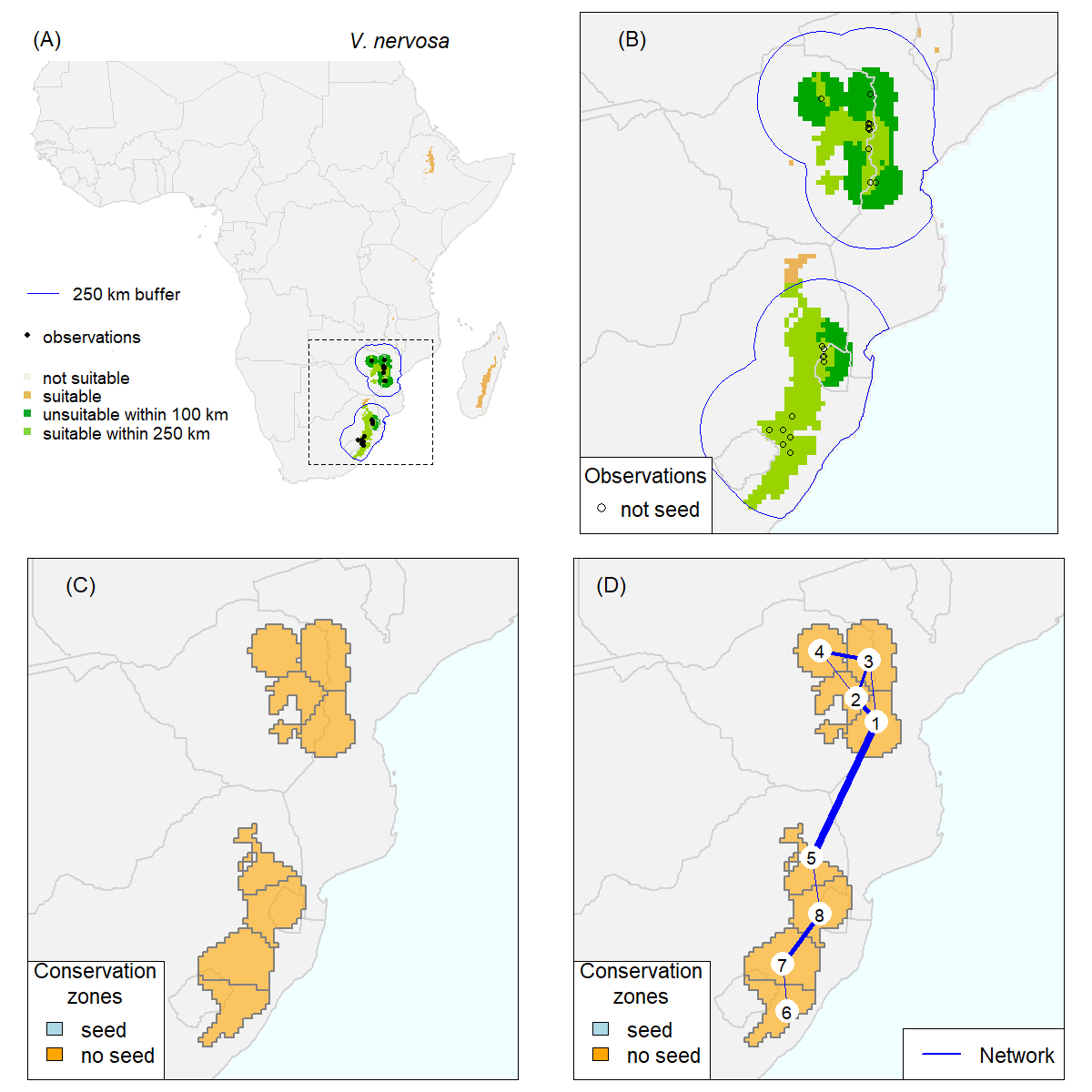

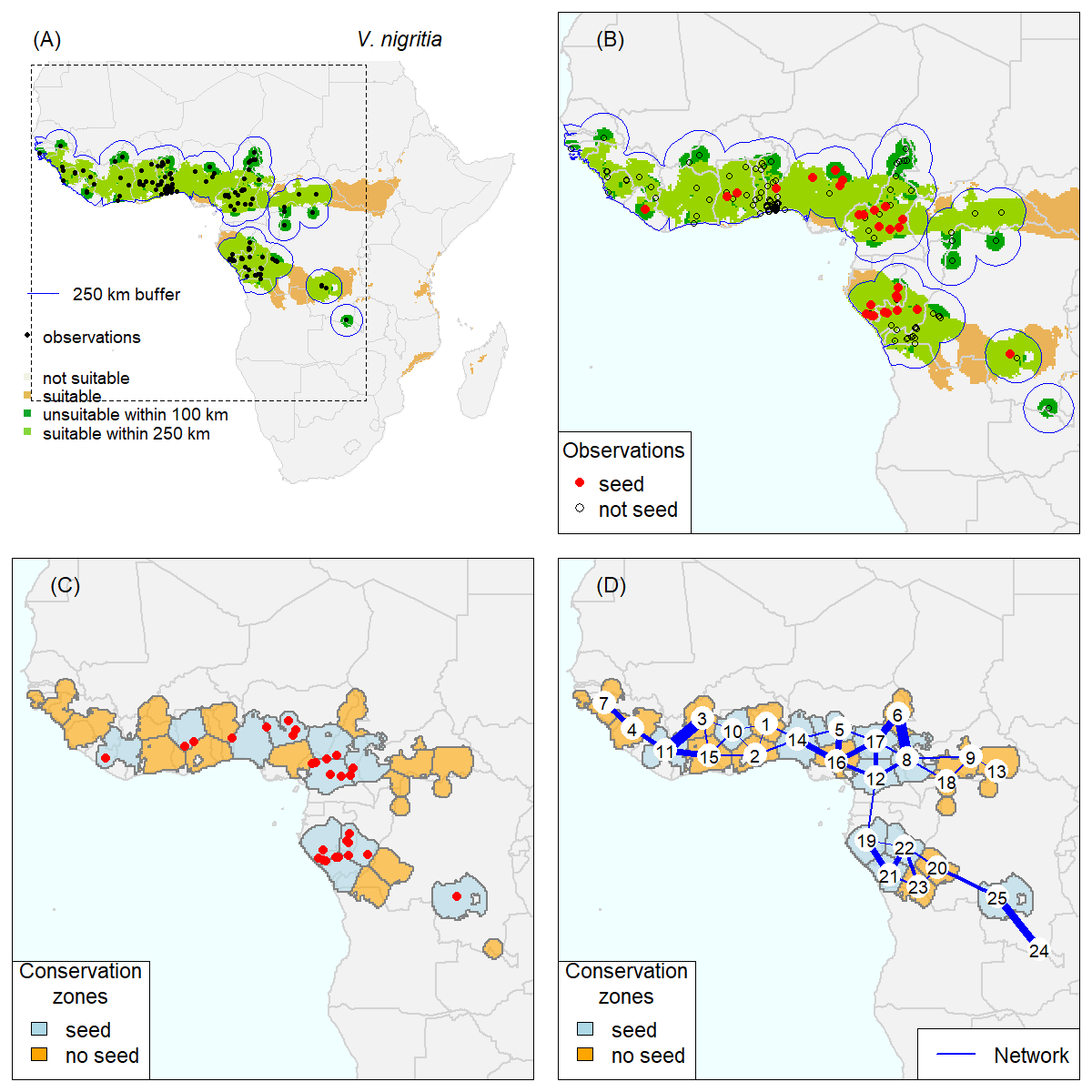

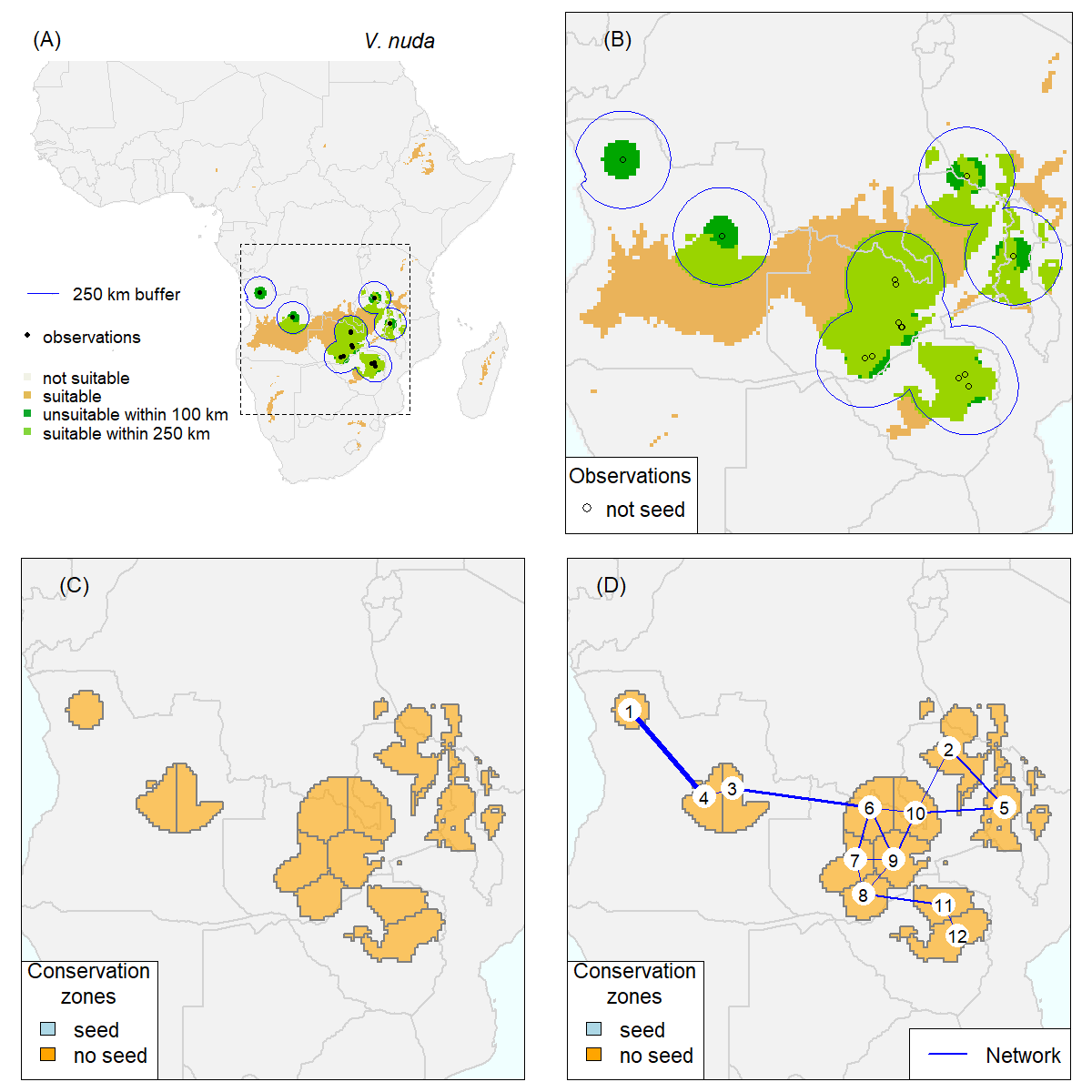

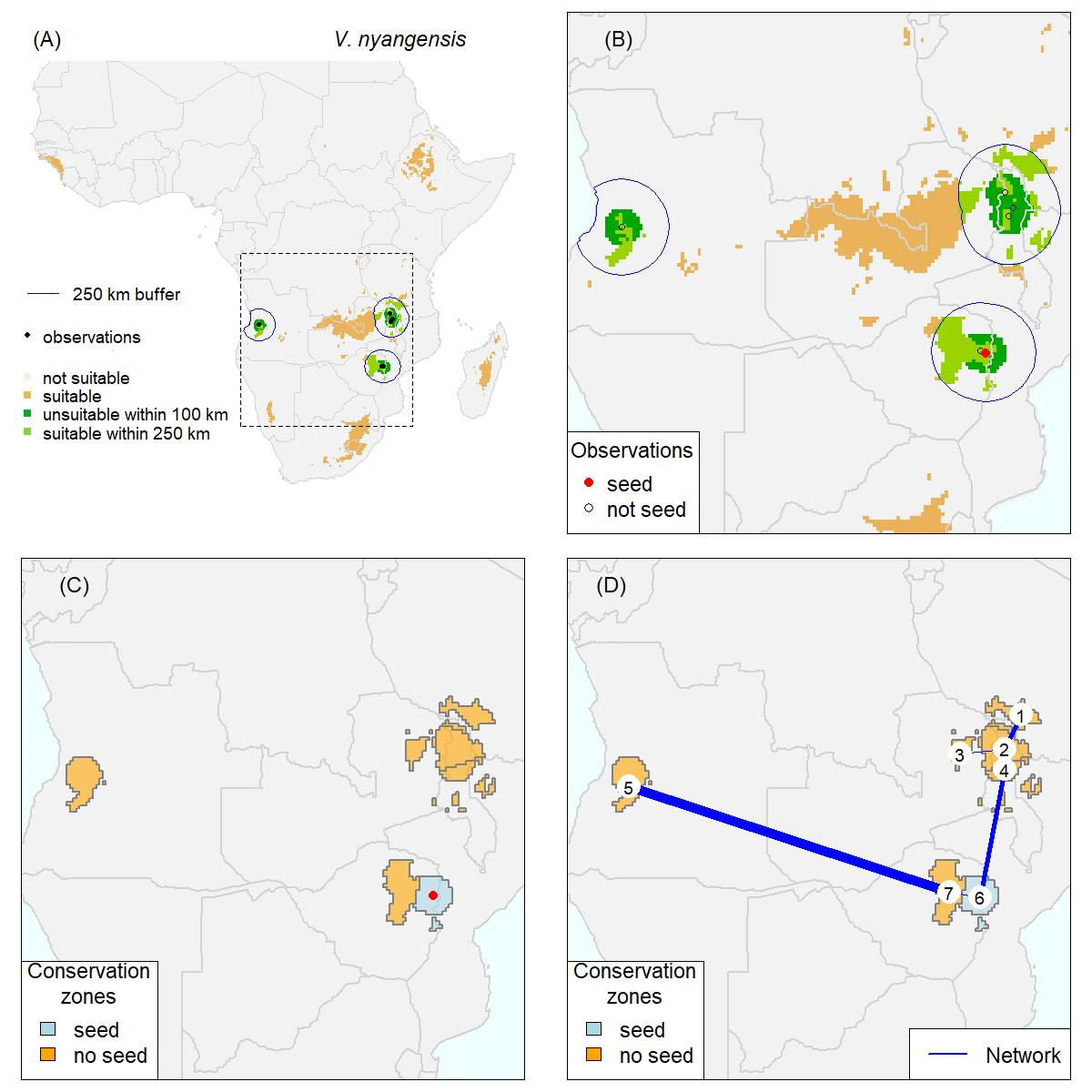

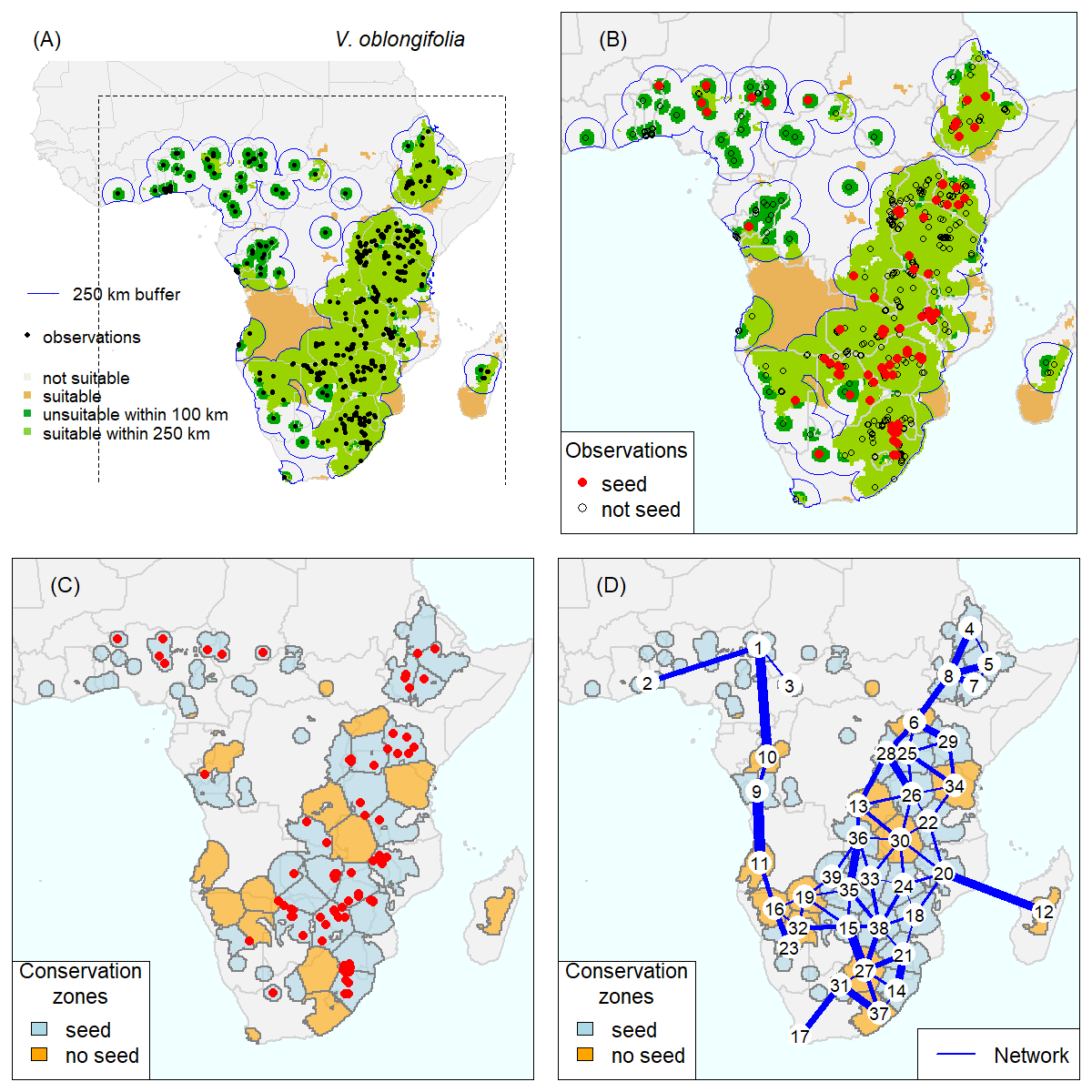

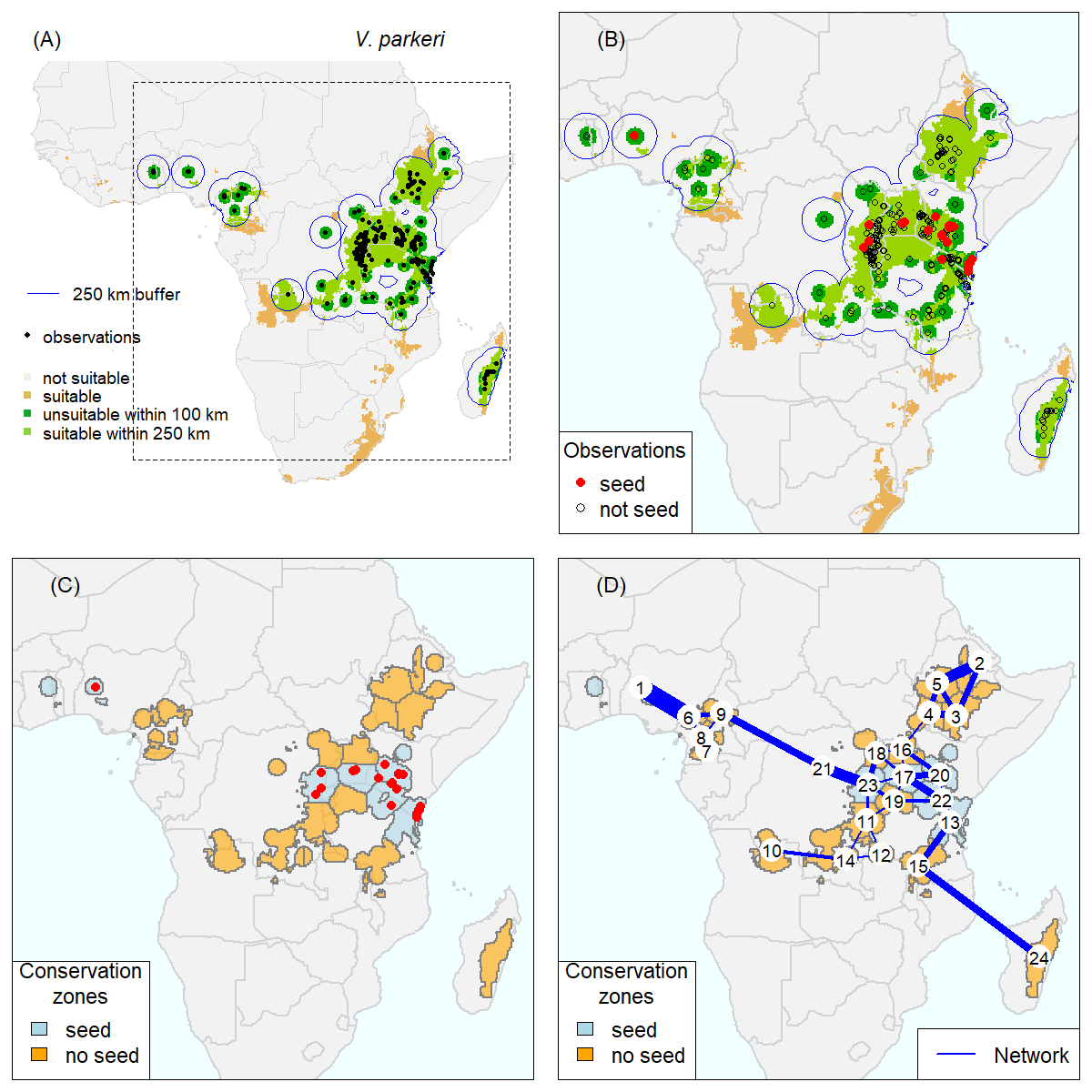

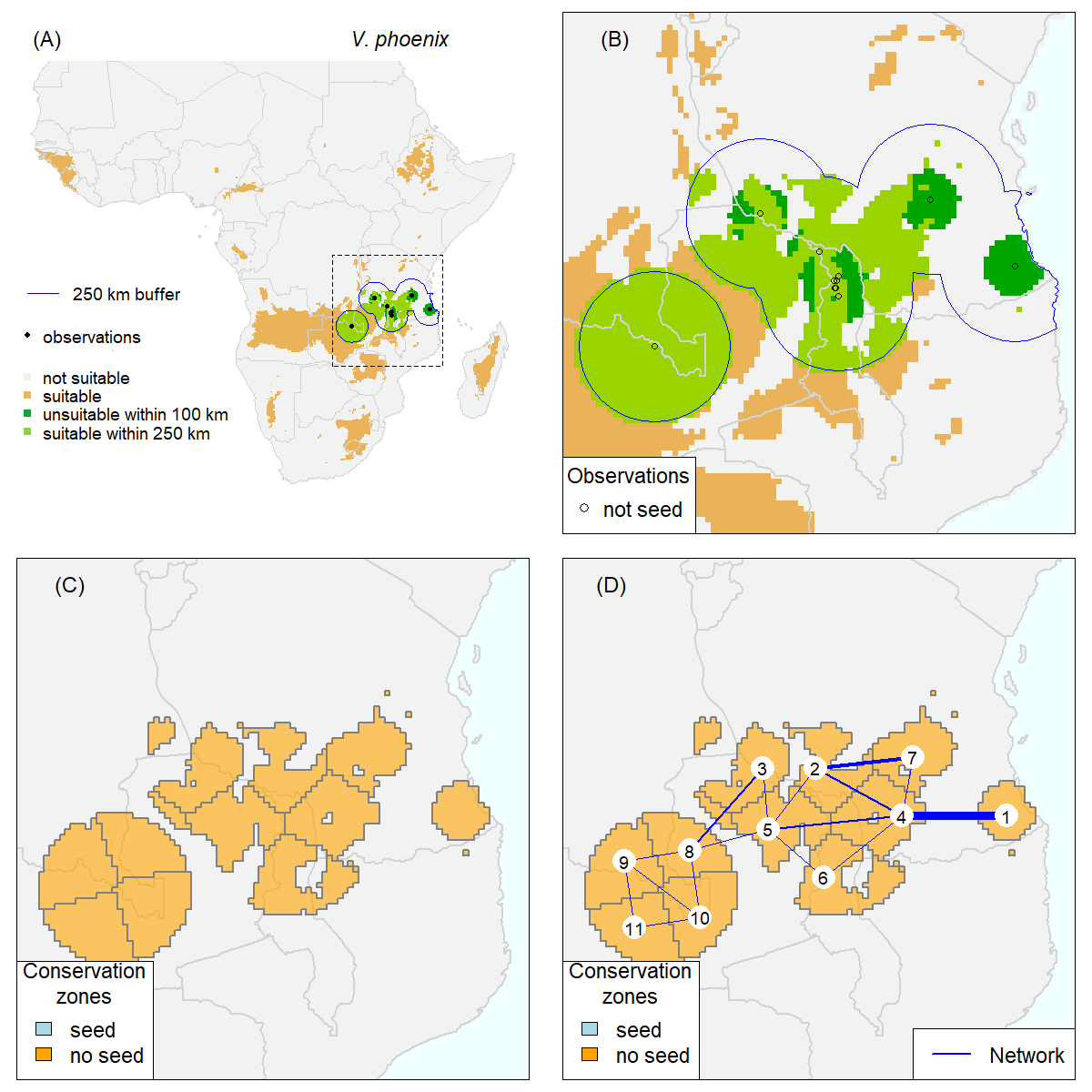

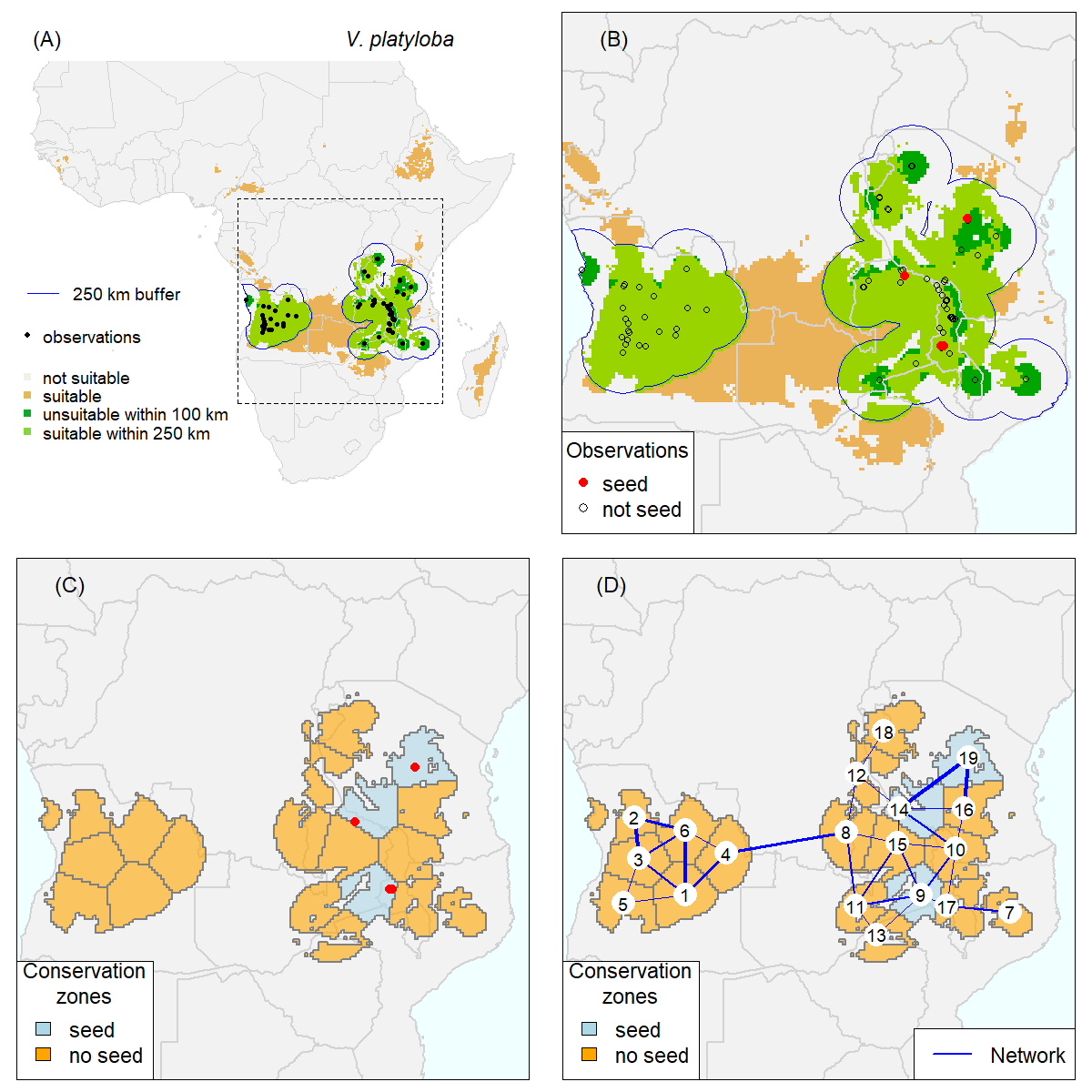

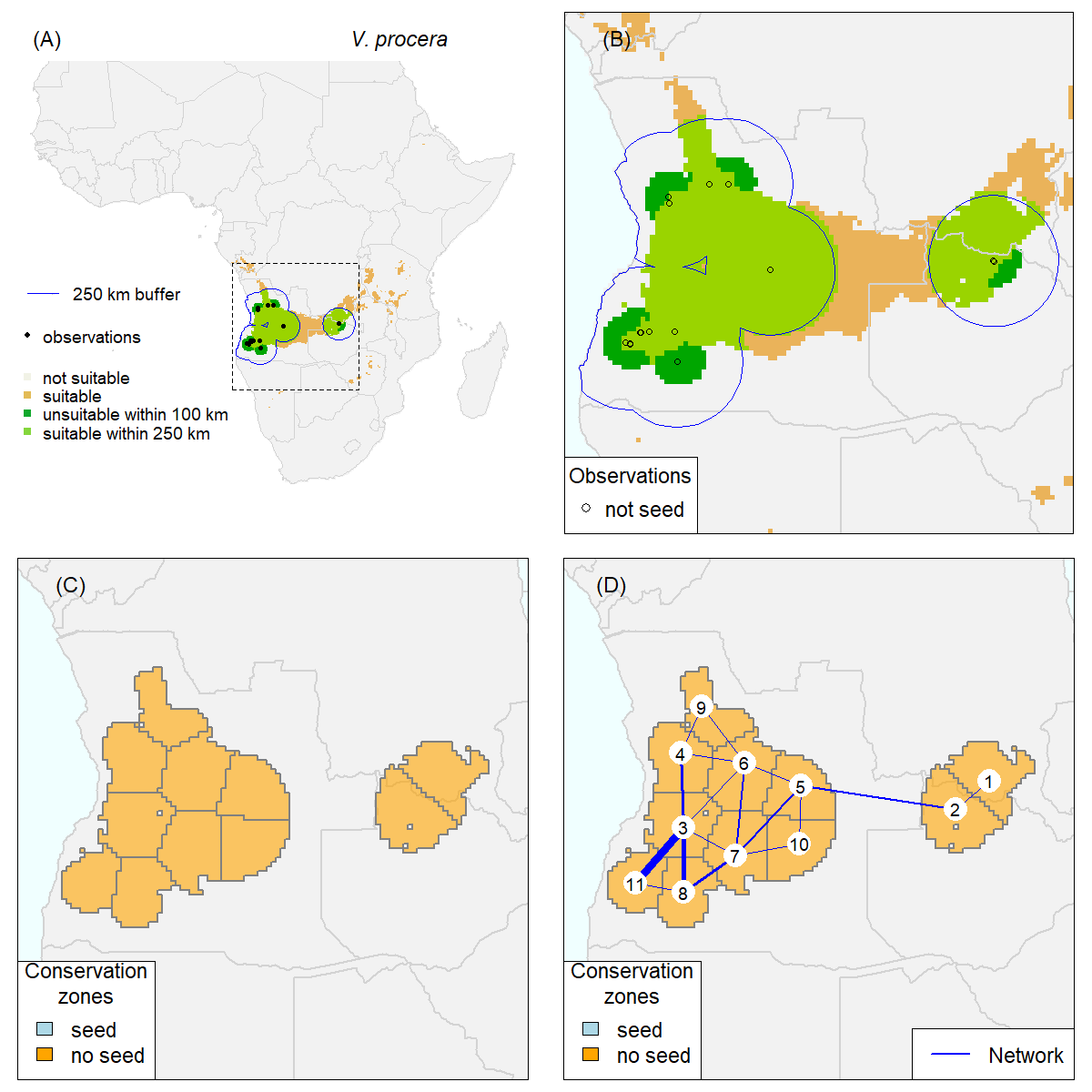

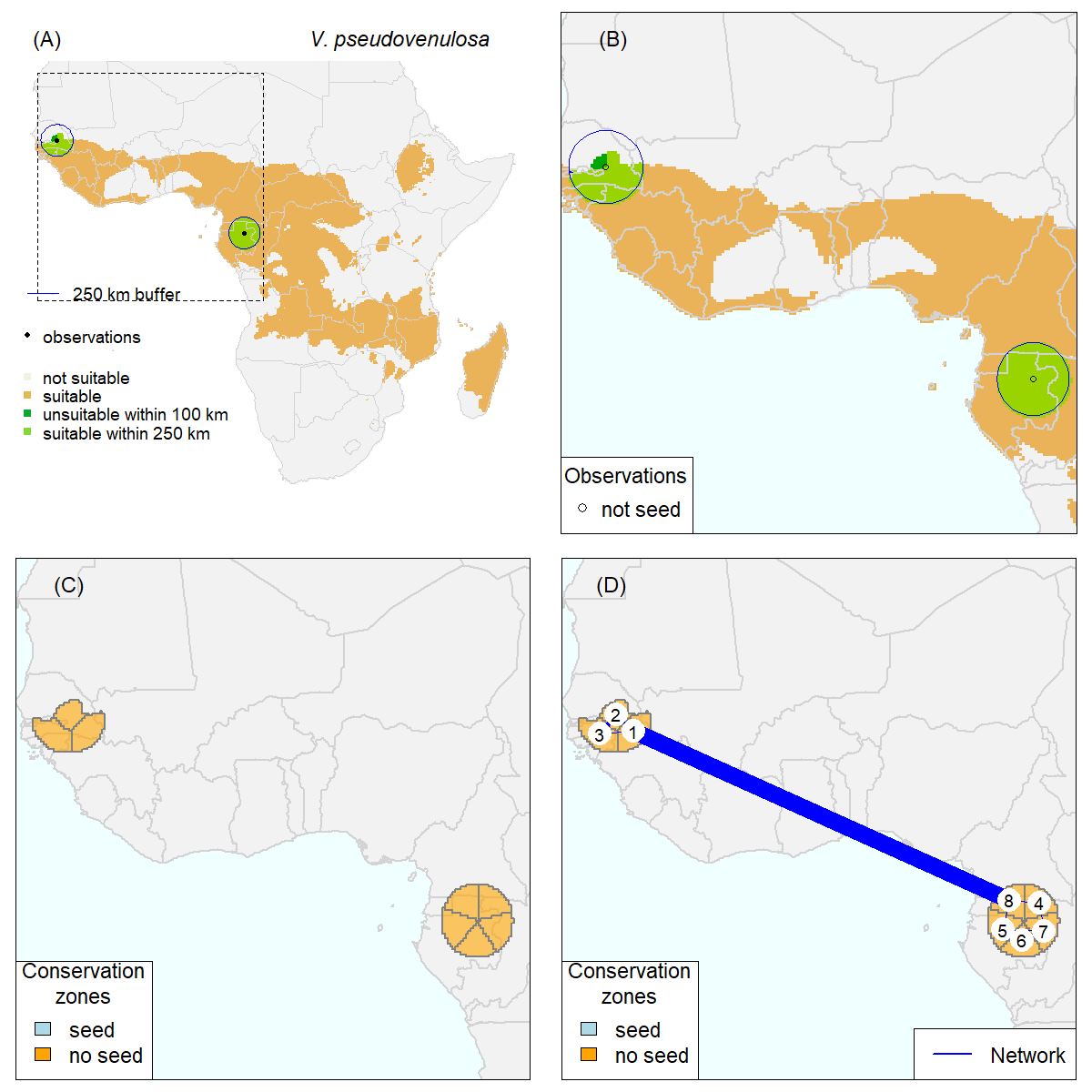

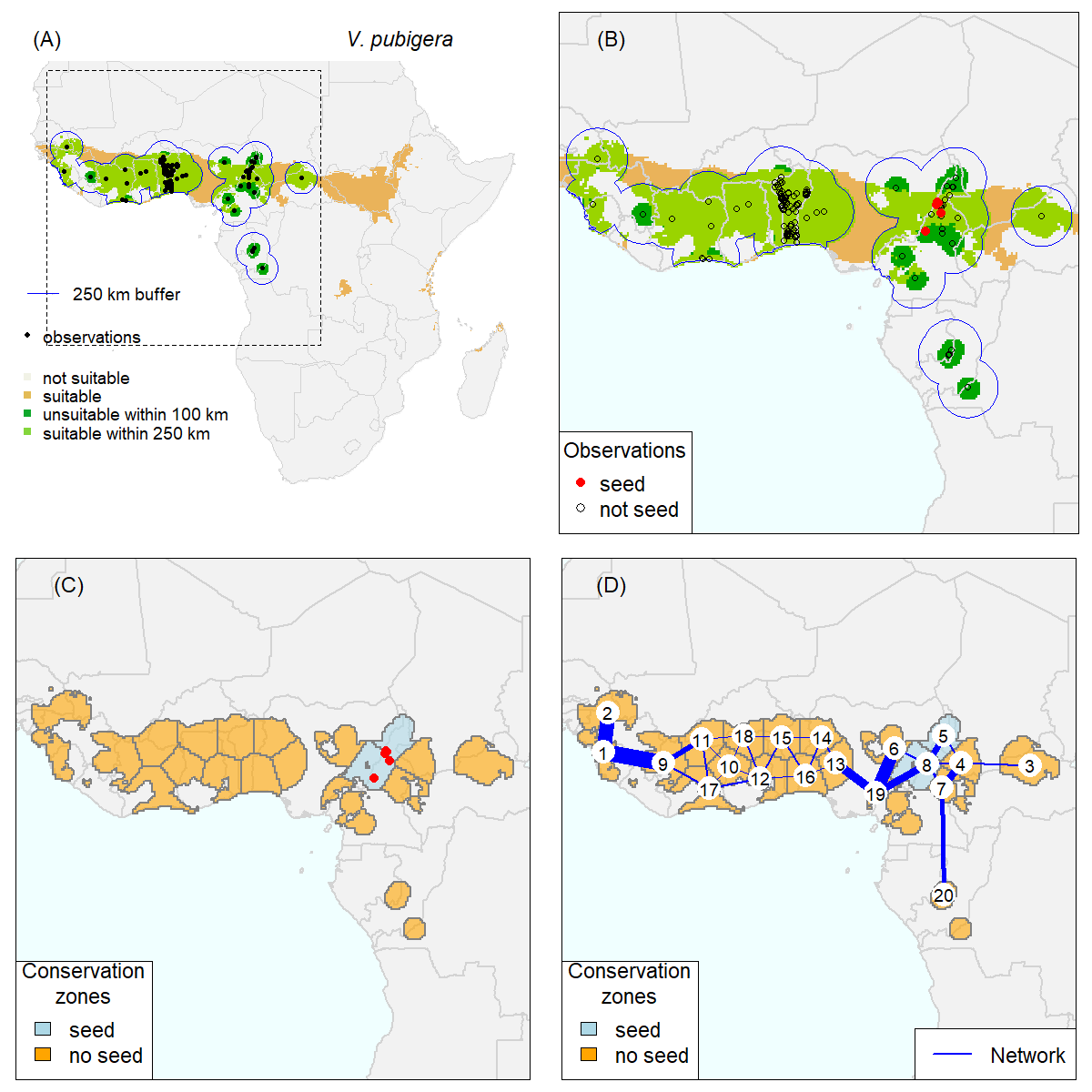

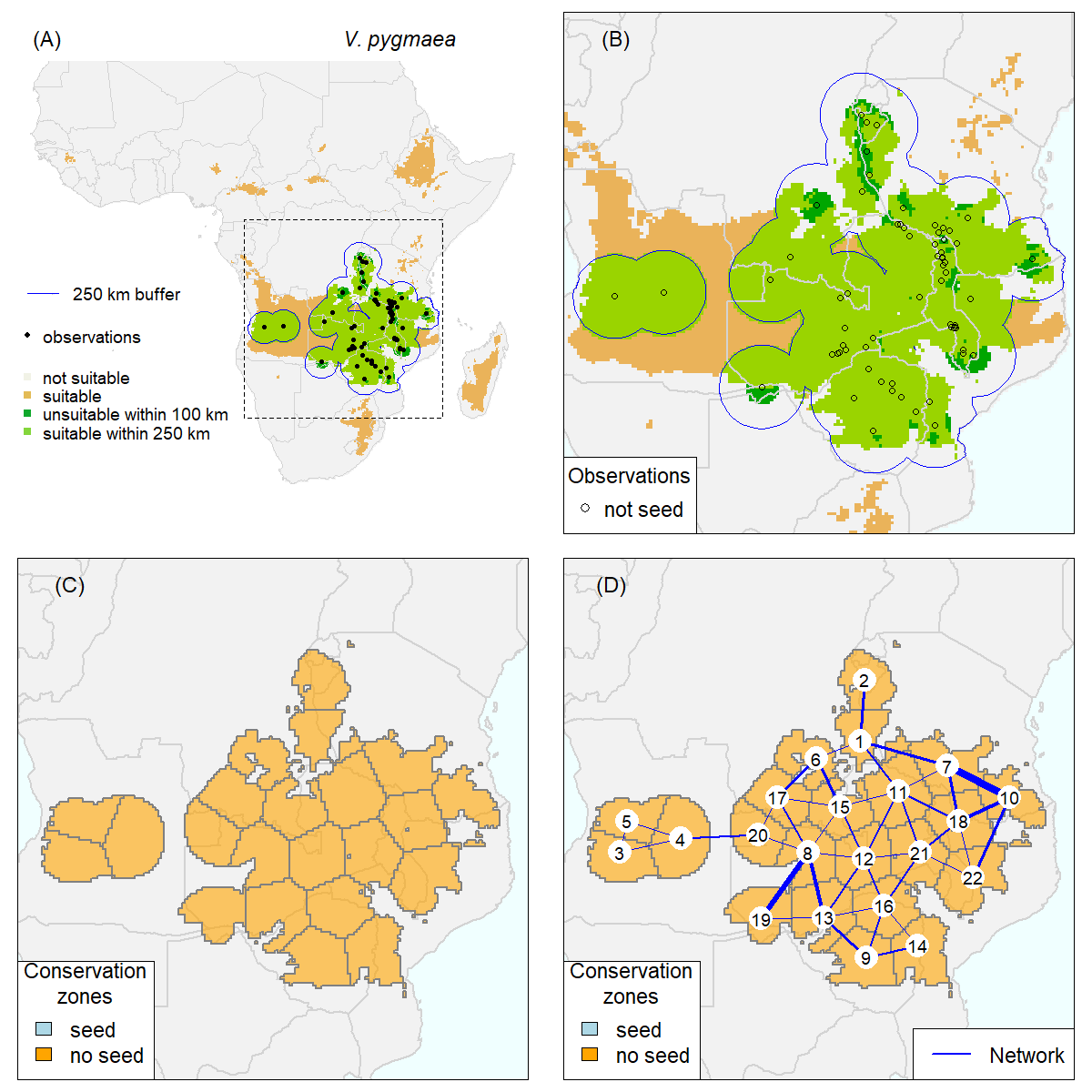

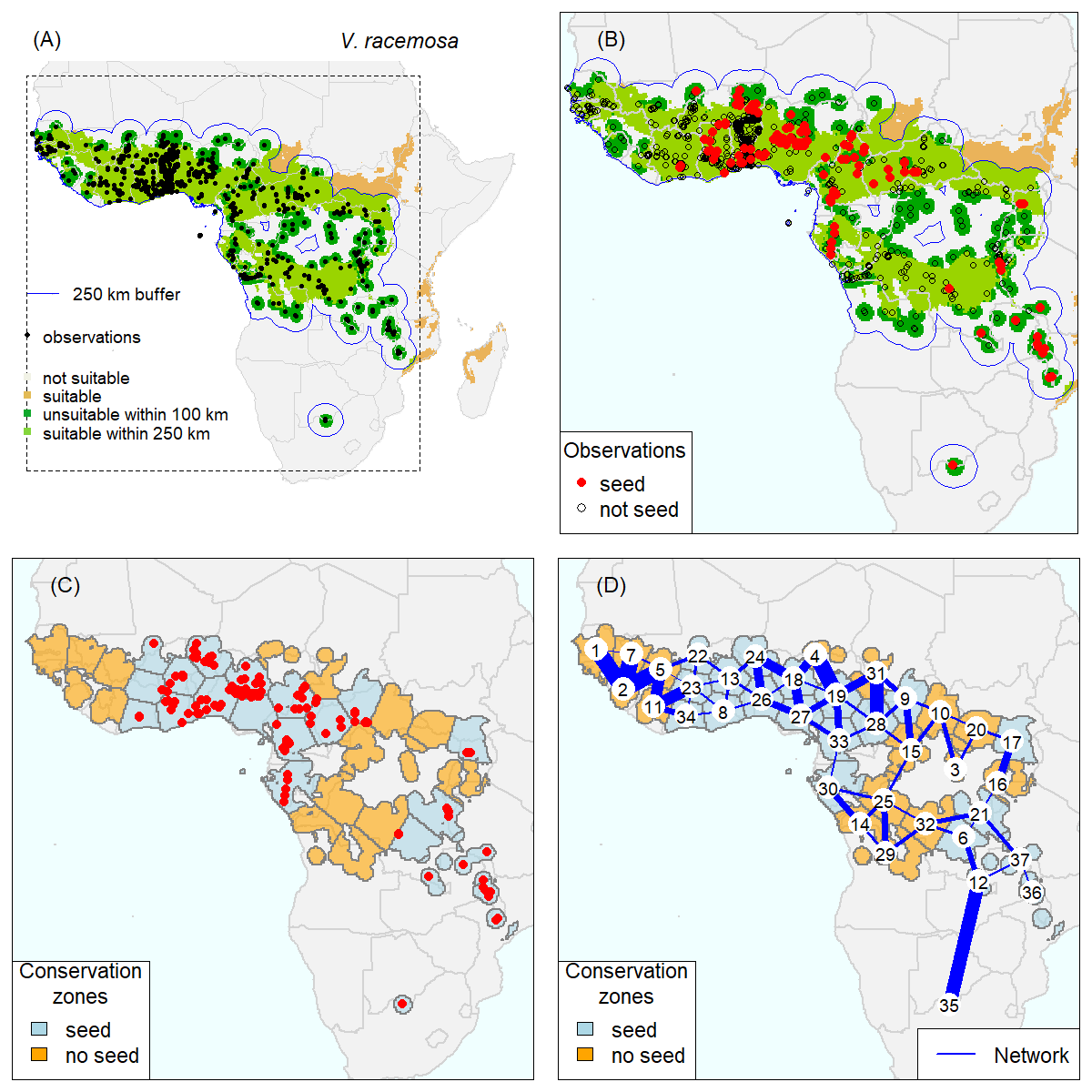

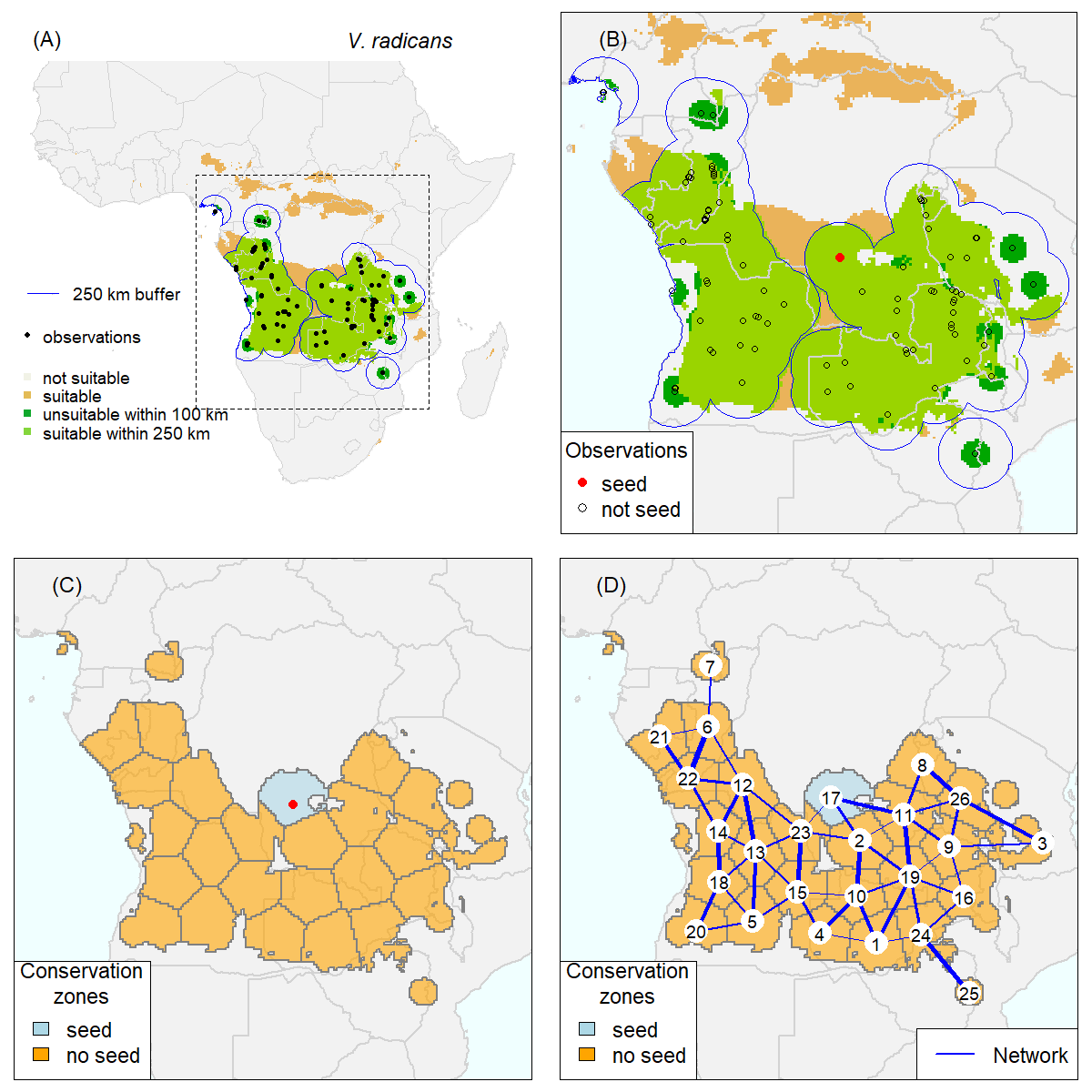

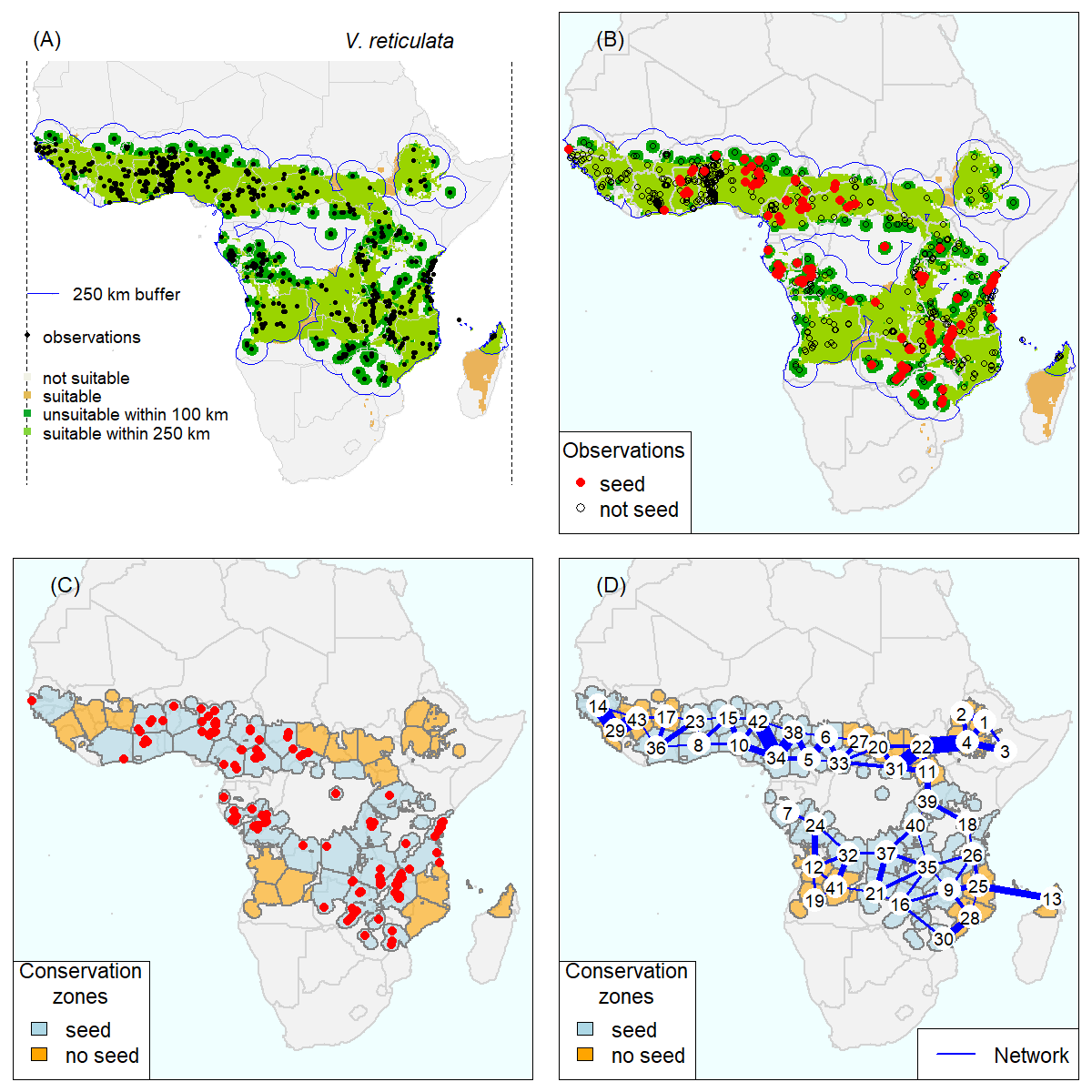

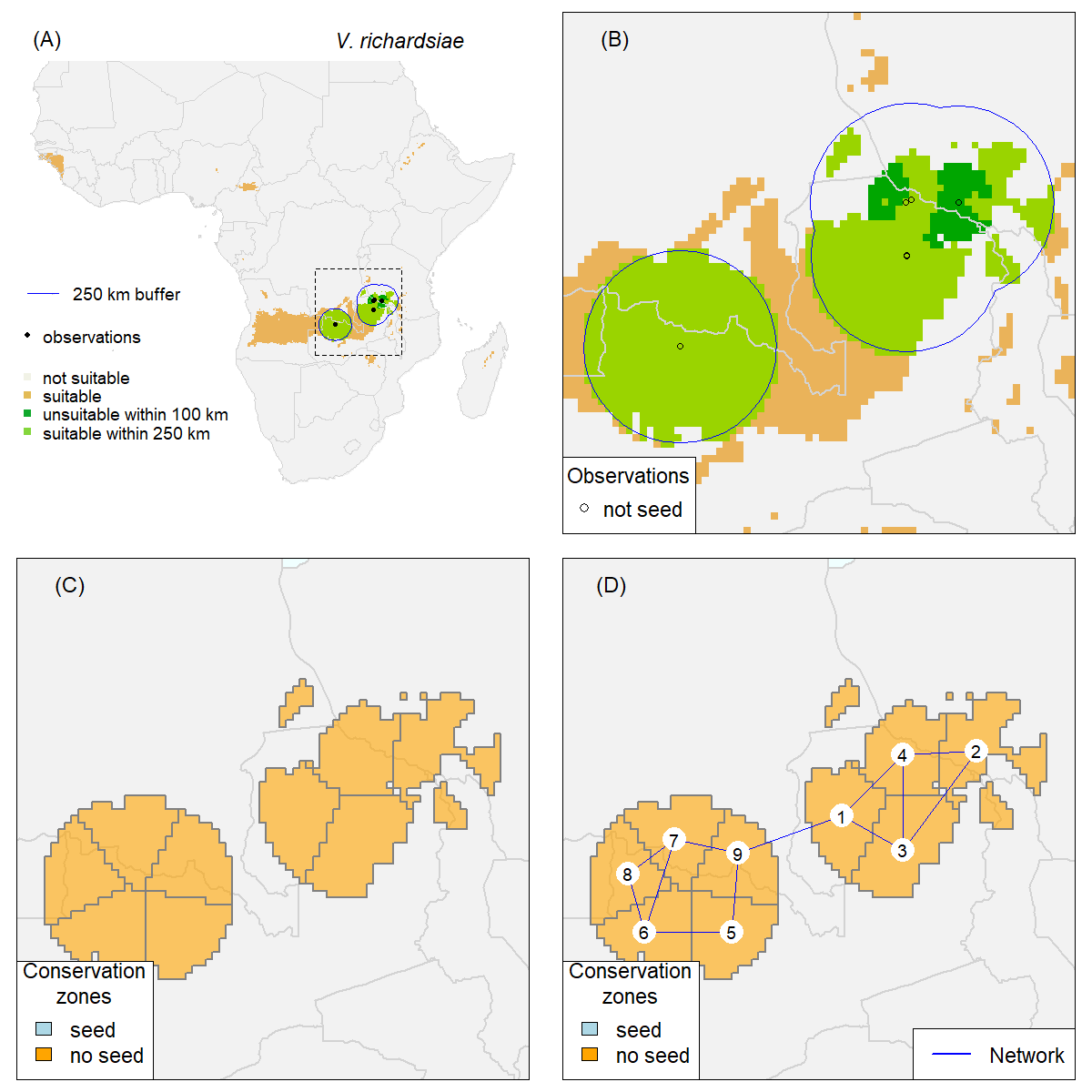

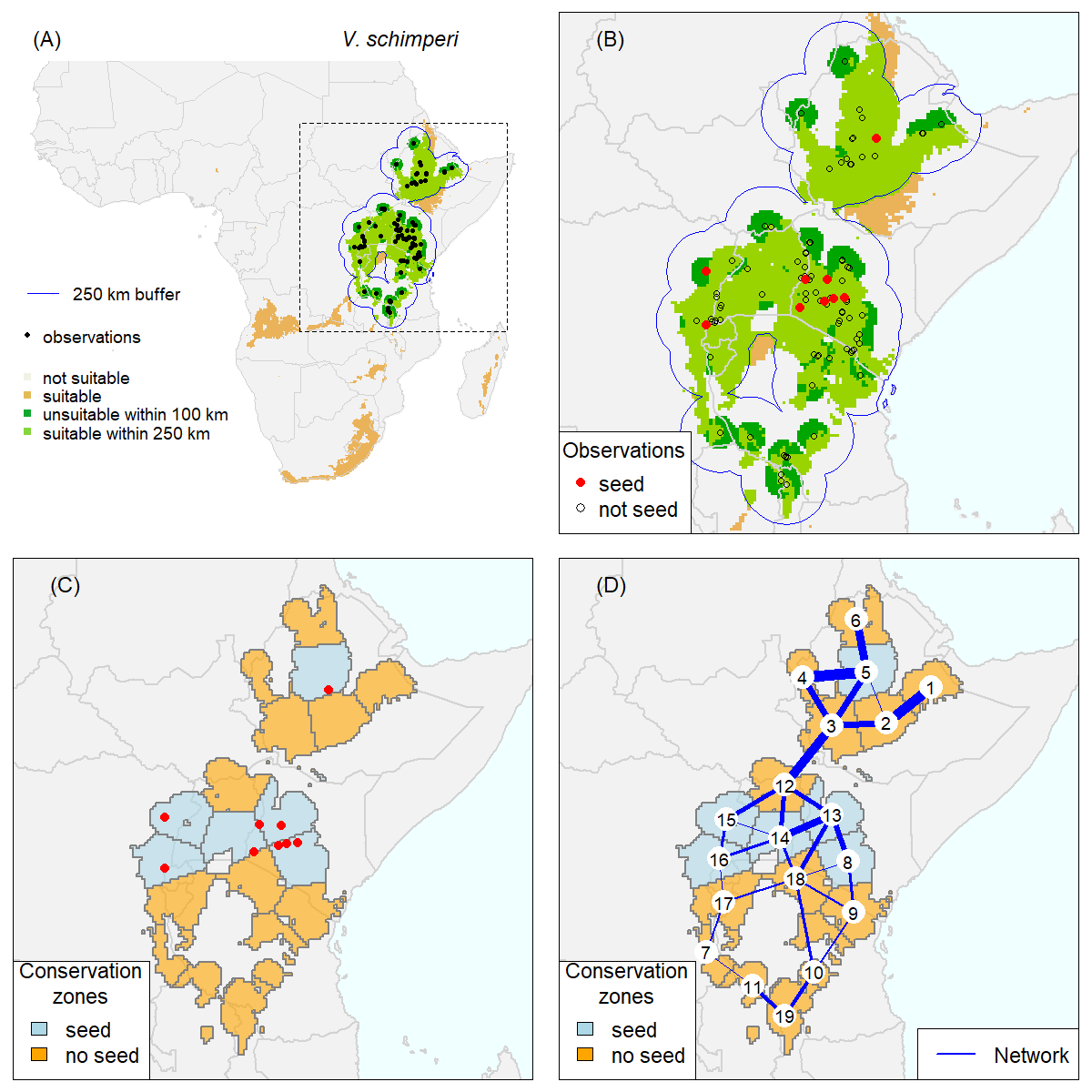

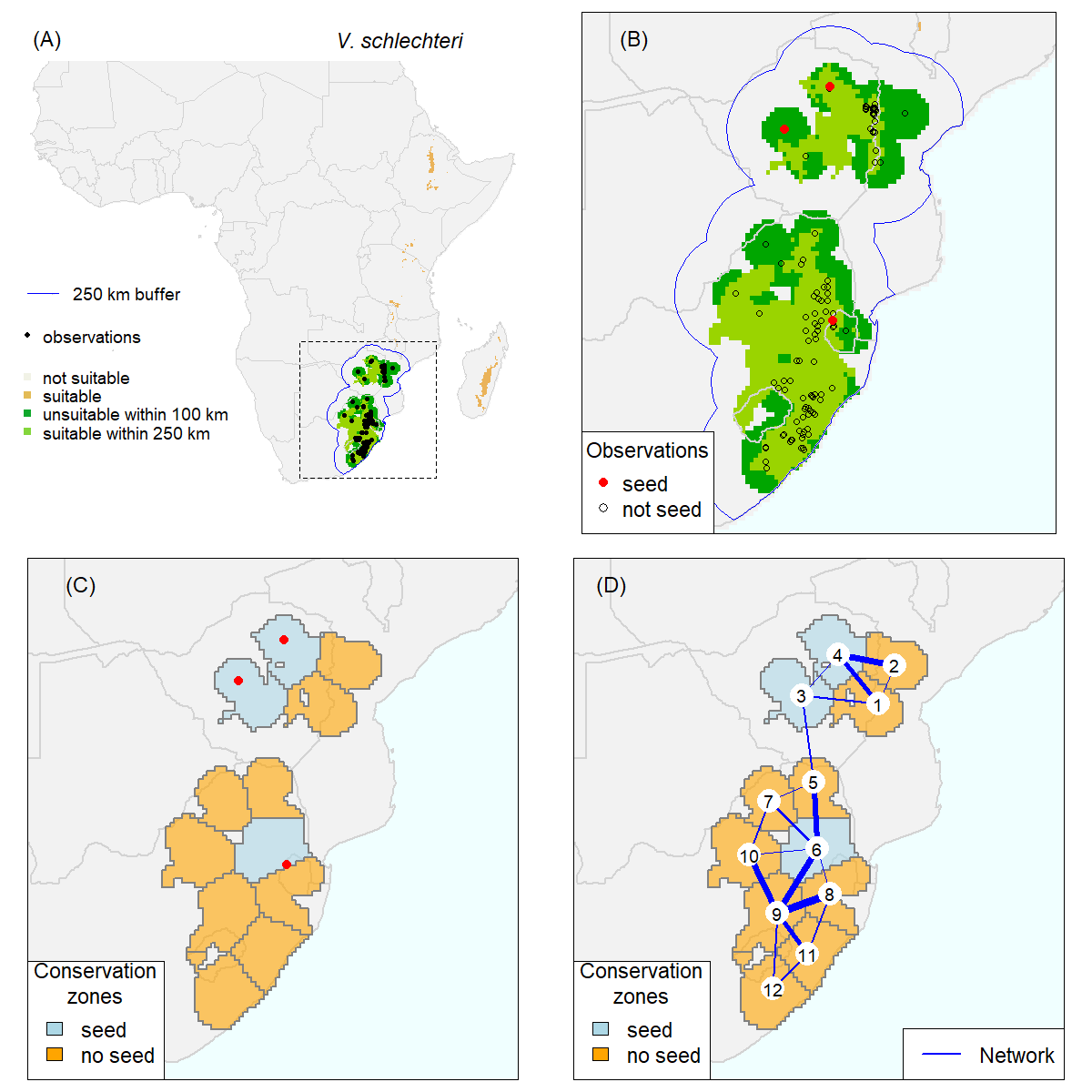

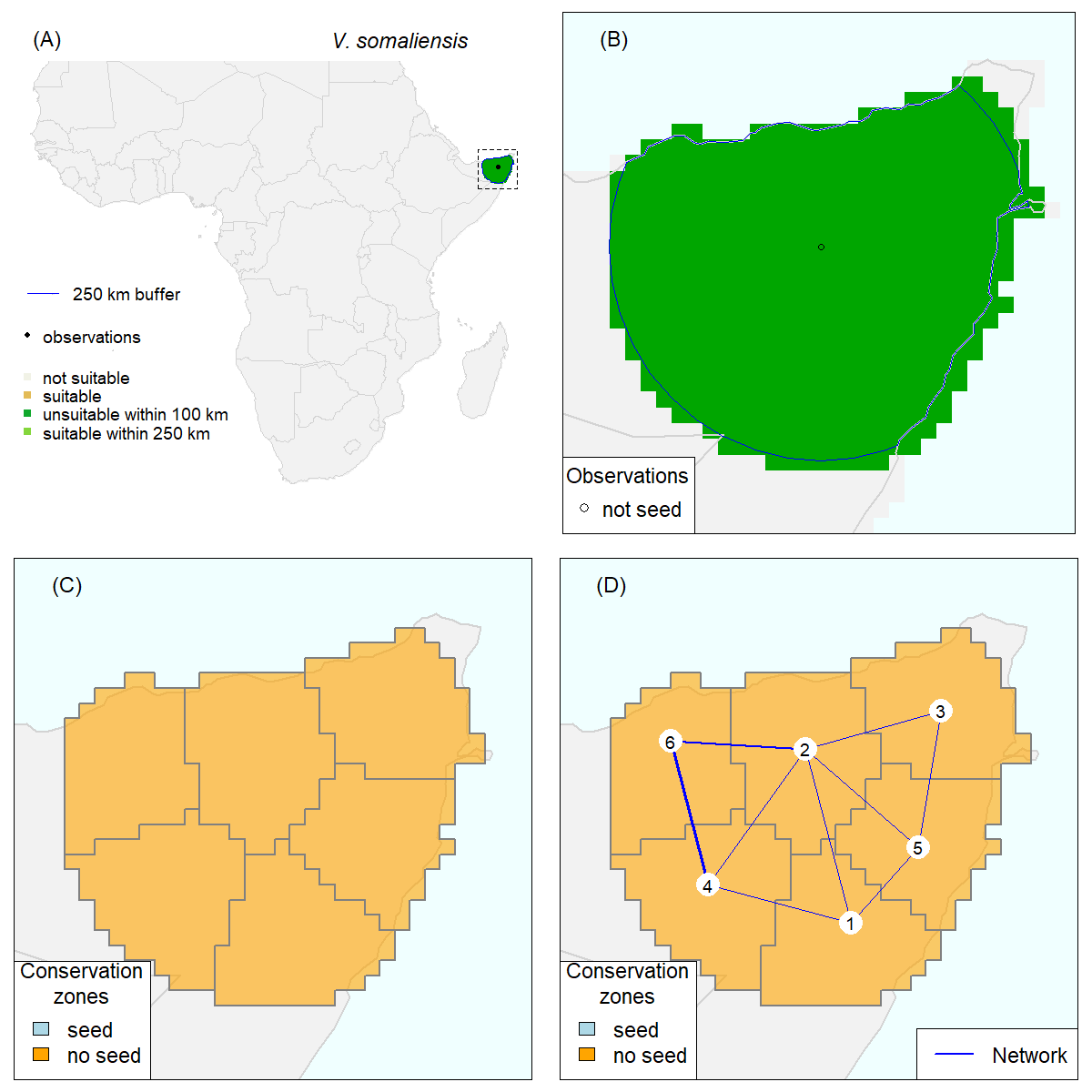

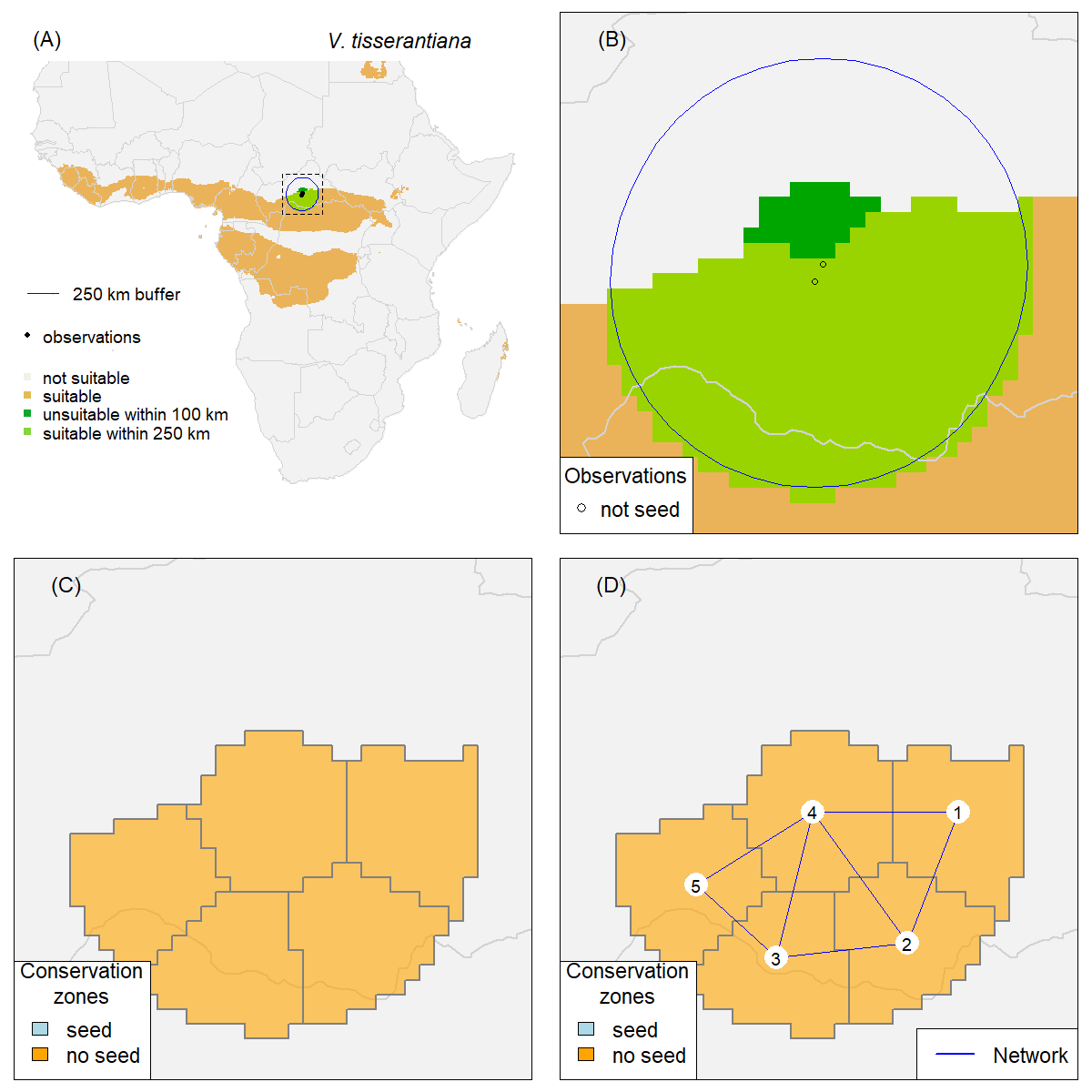

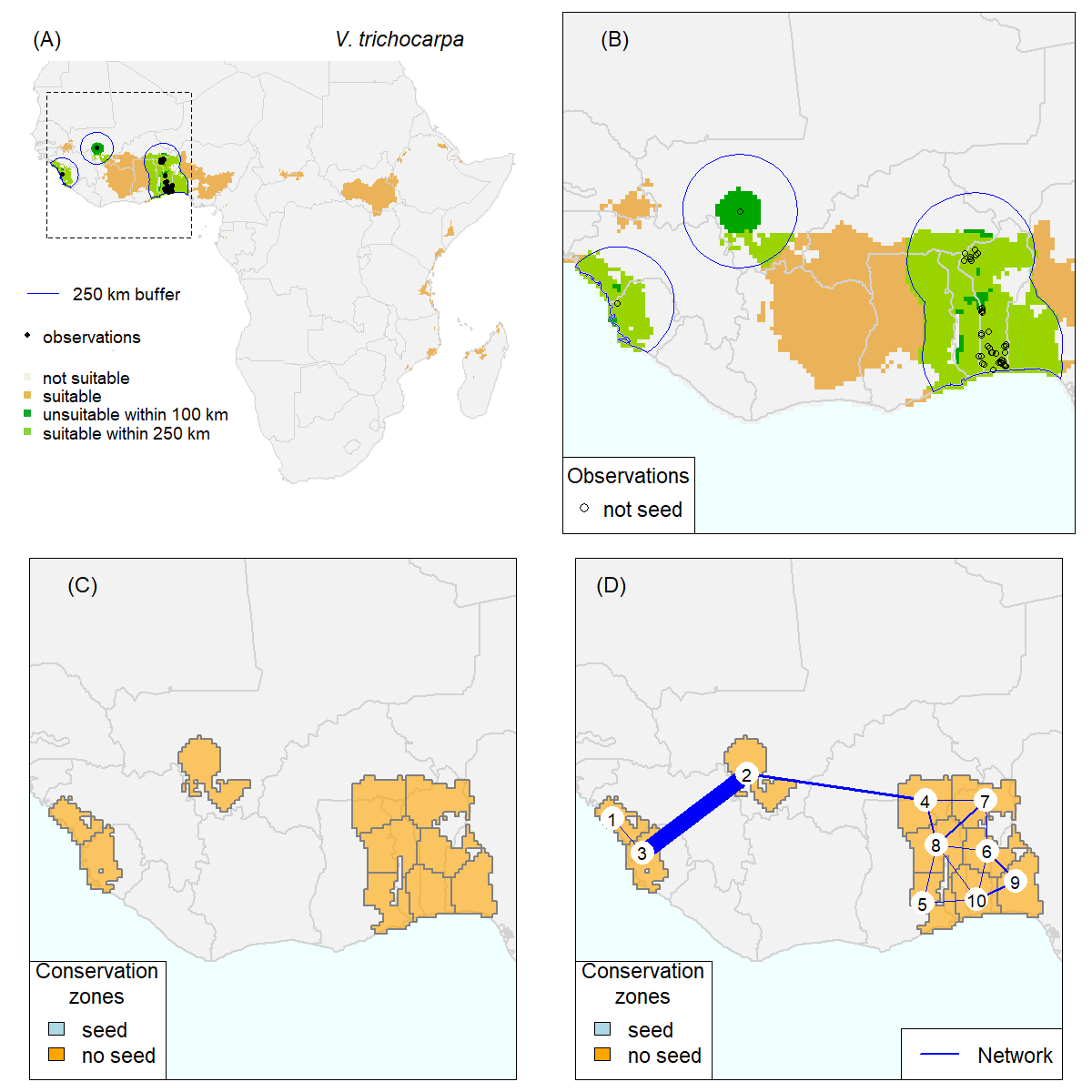

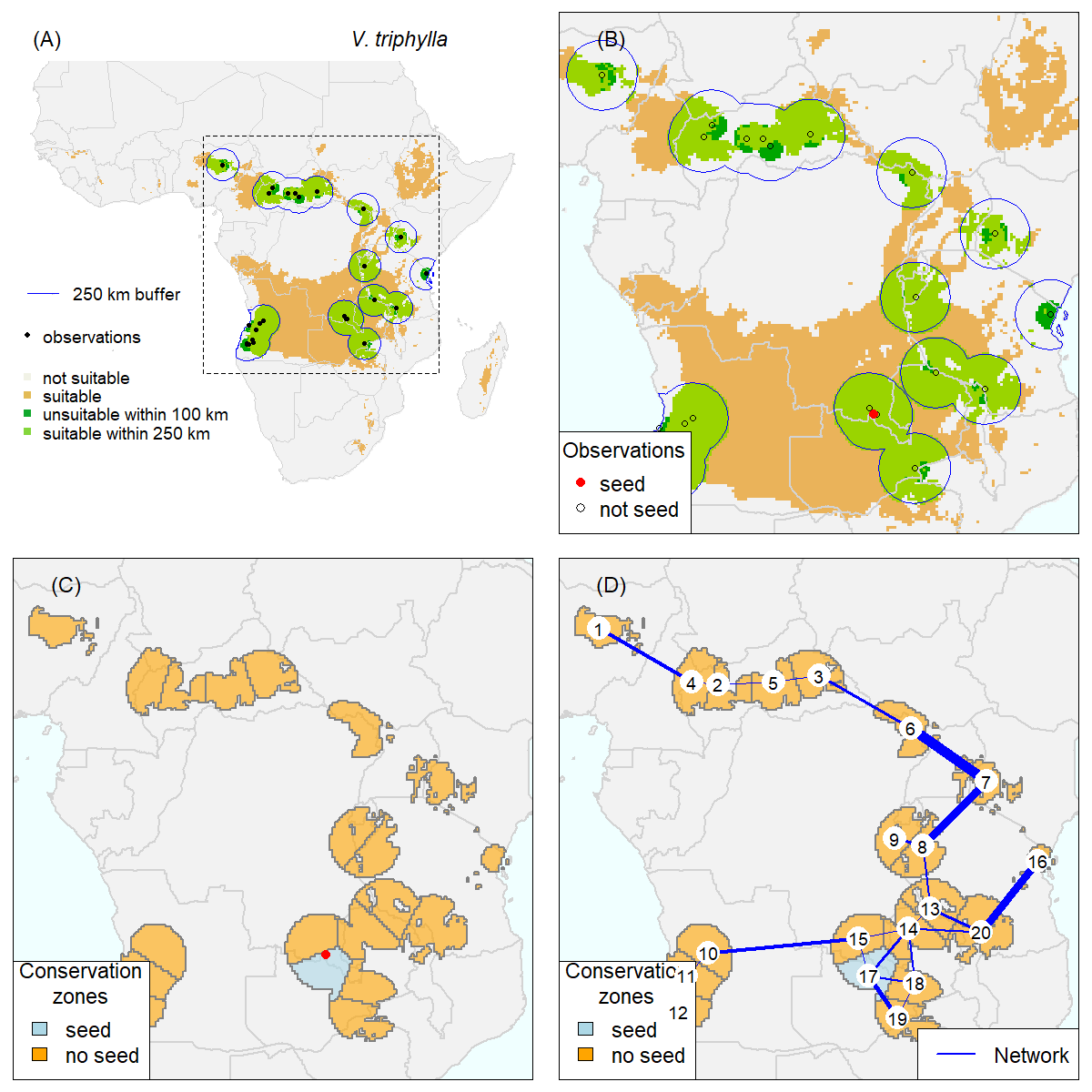

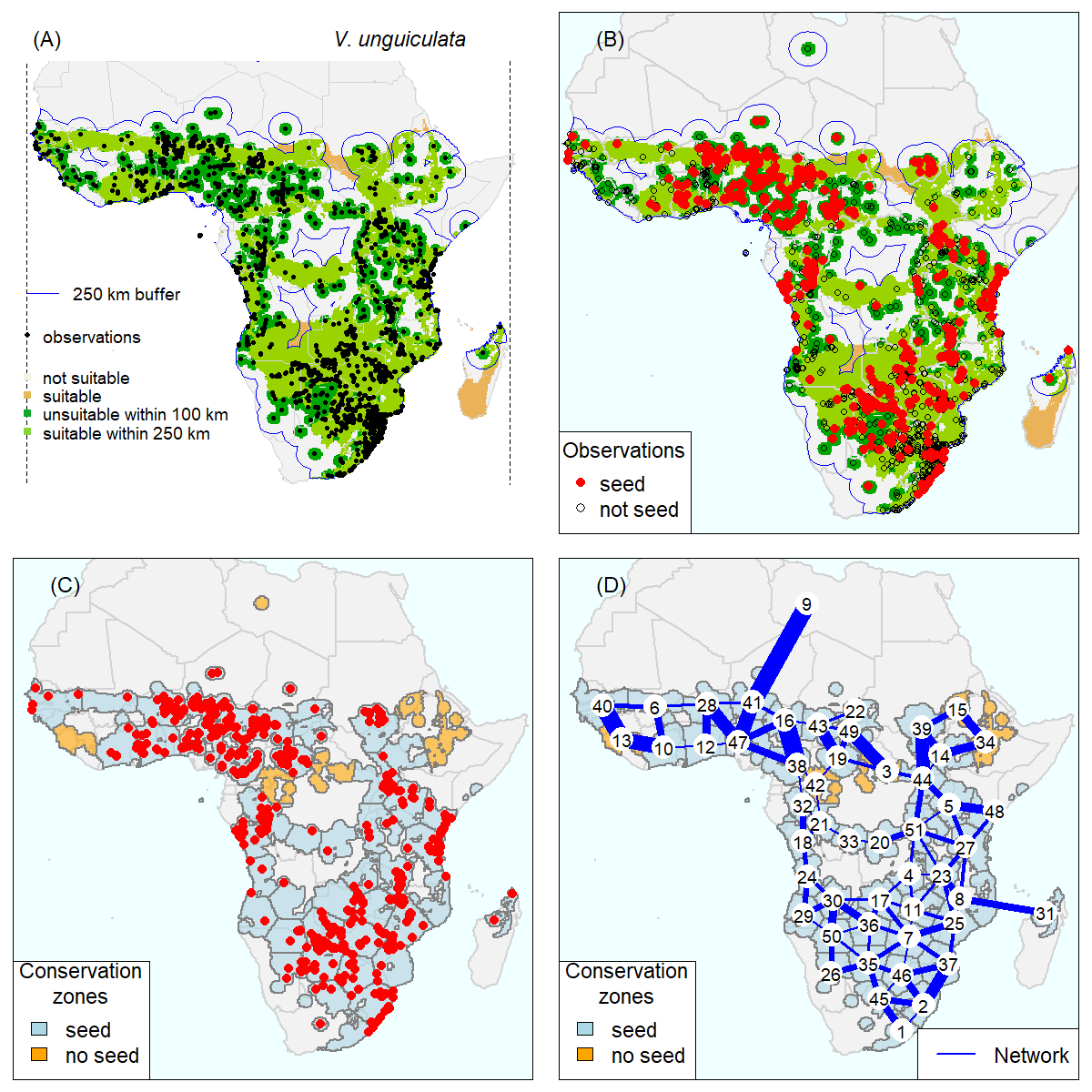

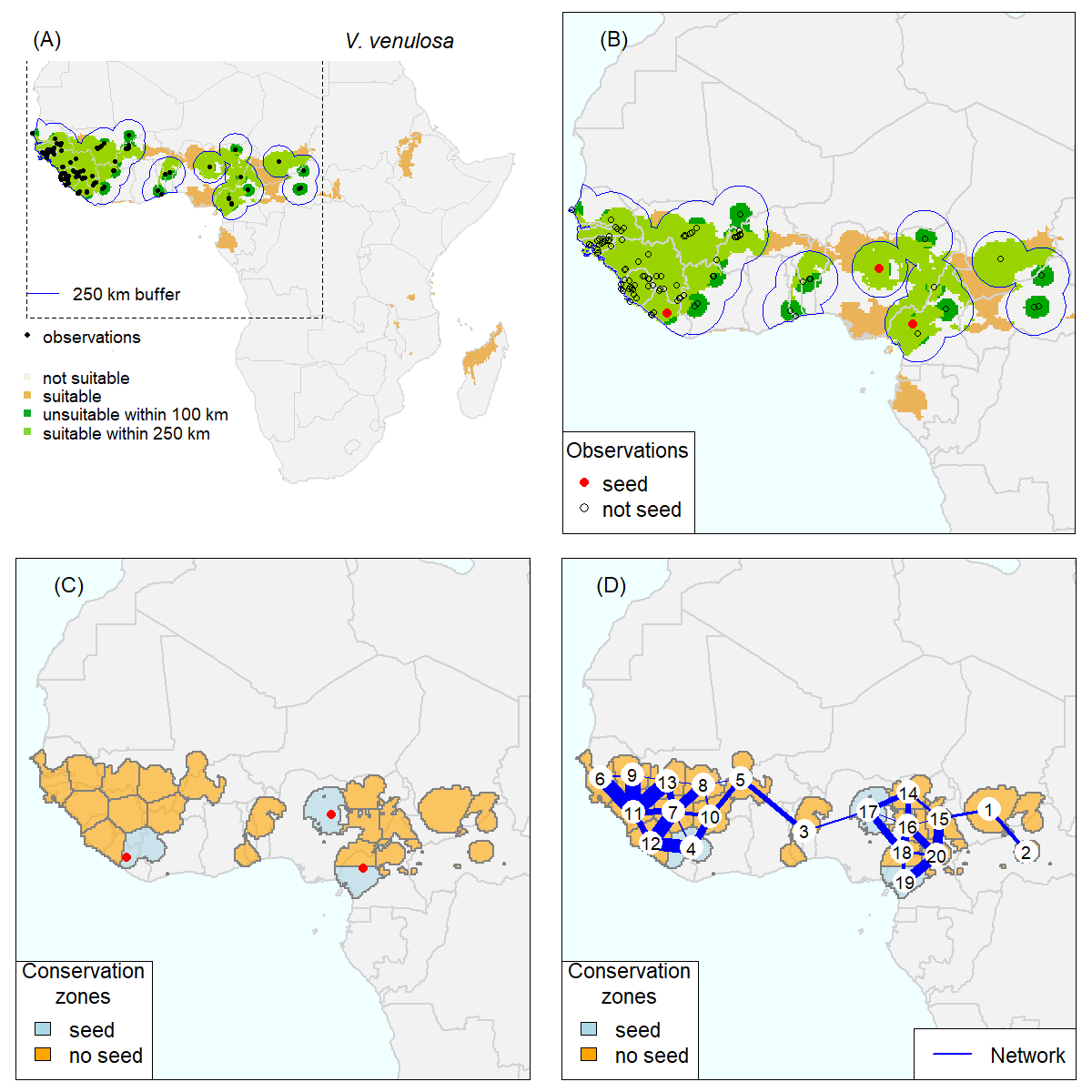

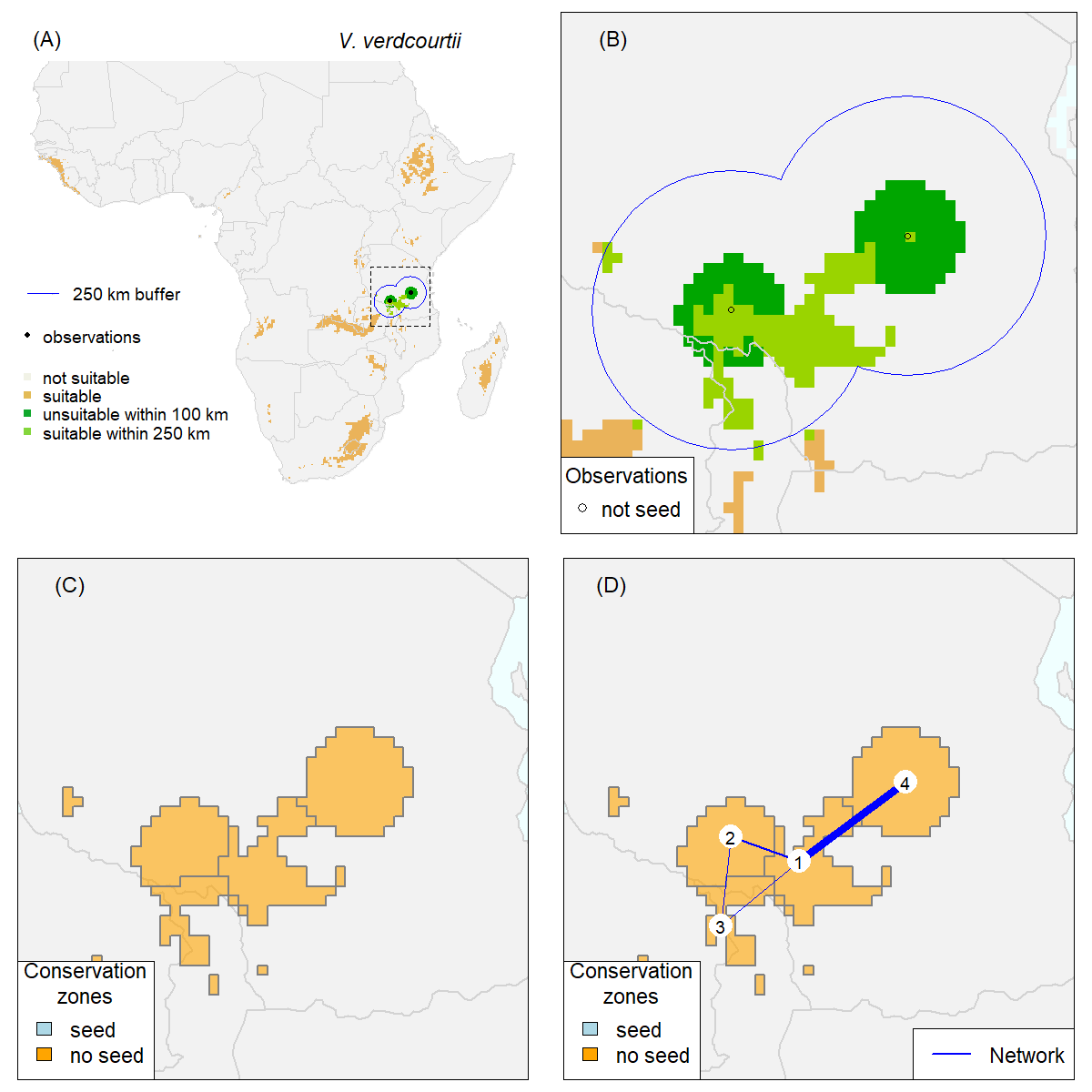

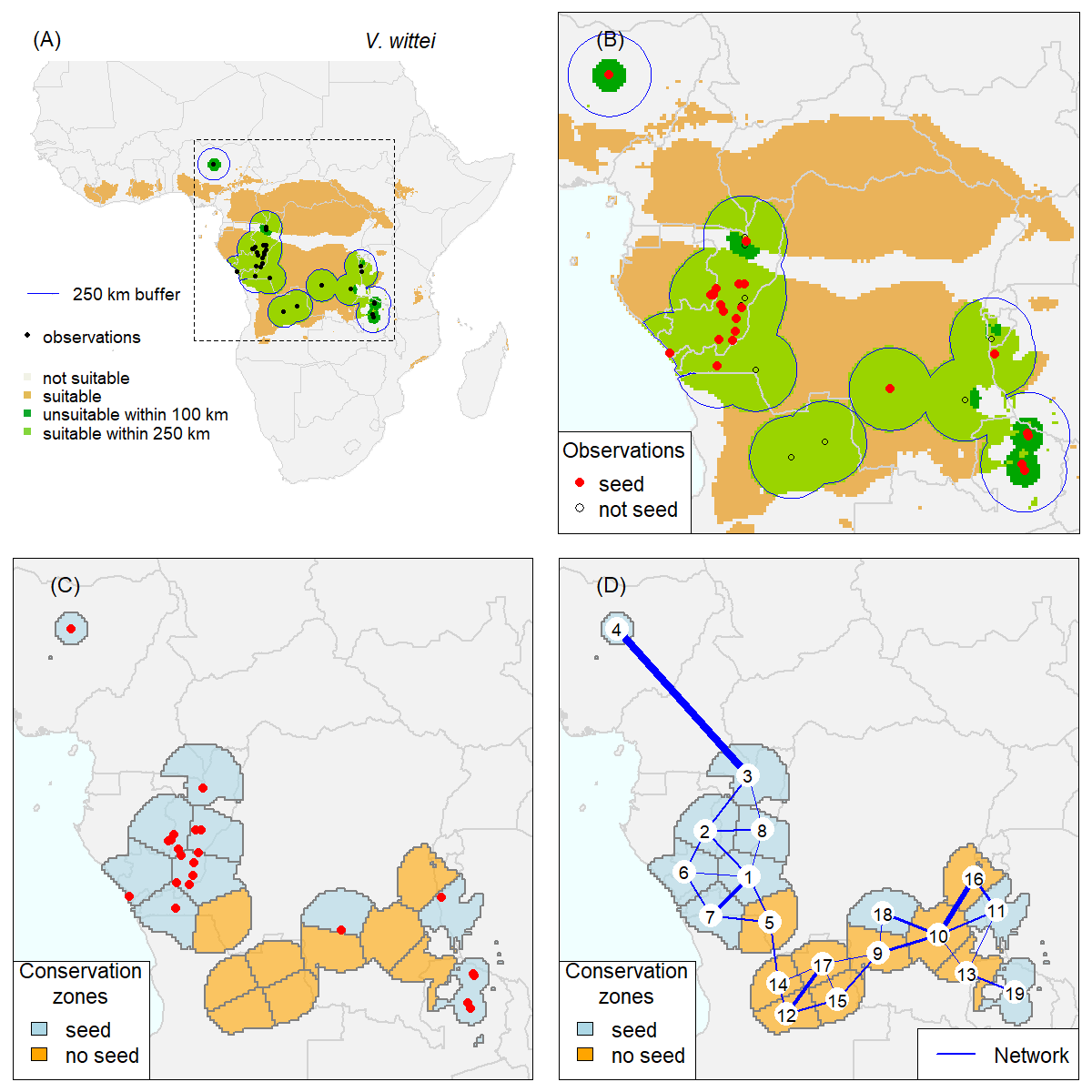

Supplement: S1 Diagrams — (DOCX) [file pone.0324820.s001.docx]
