# Supplementary material for: A distance-based framework for assessing the ex-situ conservation status of plants
Source: PLoS One. 2025 Jun 3;20(6):e0324820. doi: 10.1371/journal.pone.0324820 (PMC12133166; doi:10.1371/journal.pone.0324820)
Supplement: S1 Figures — (DOCX) [file pone.0324820.s002.docx]

# Supplementary information for El Graoui et al., A distance-based framework for assessing the ex-situ conservation status of plants


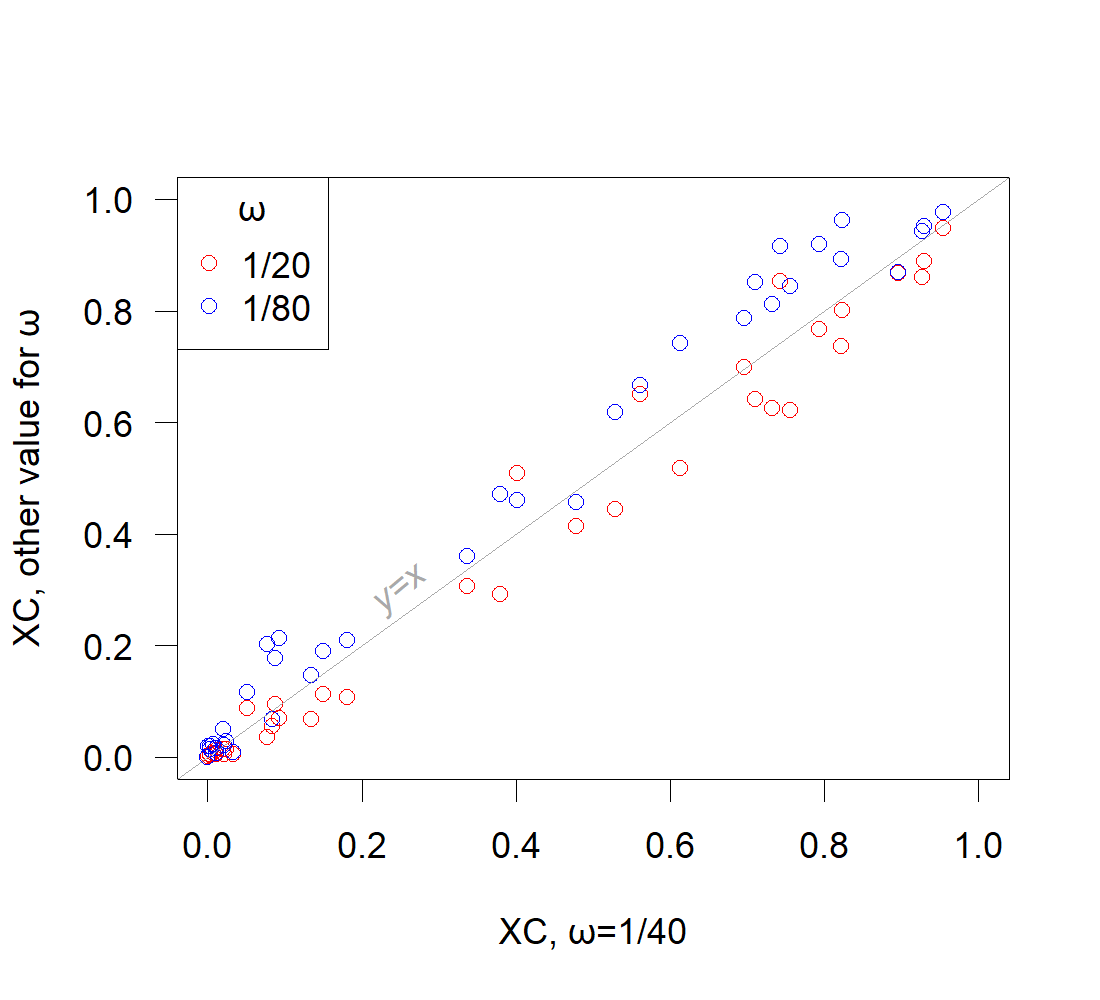


Fig. I: Sensitivity of the *XC* score to the value of ω in Equation 1.


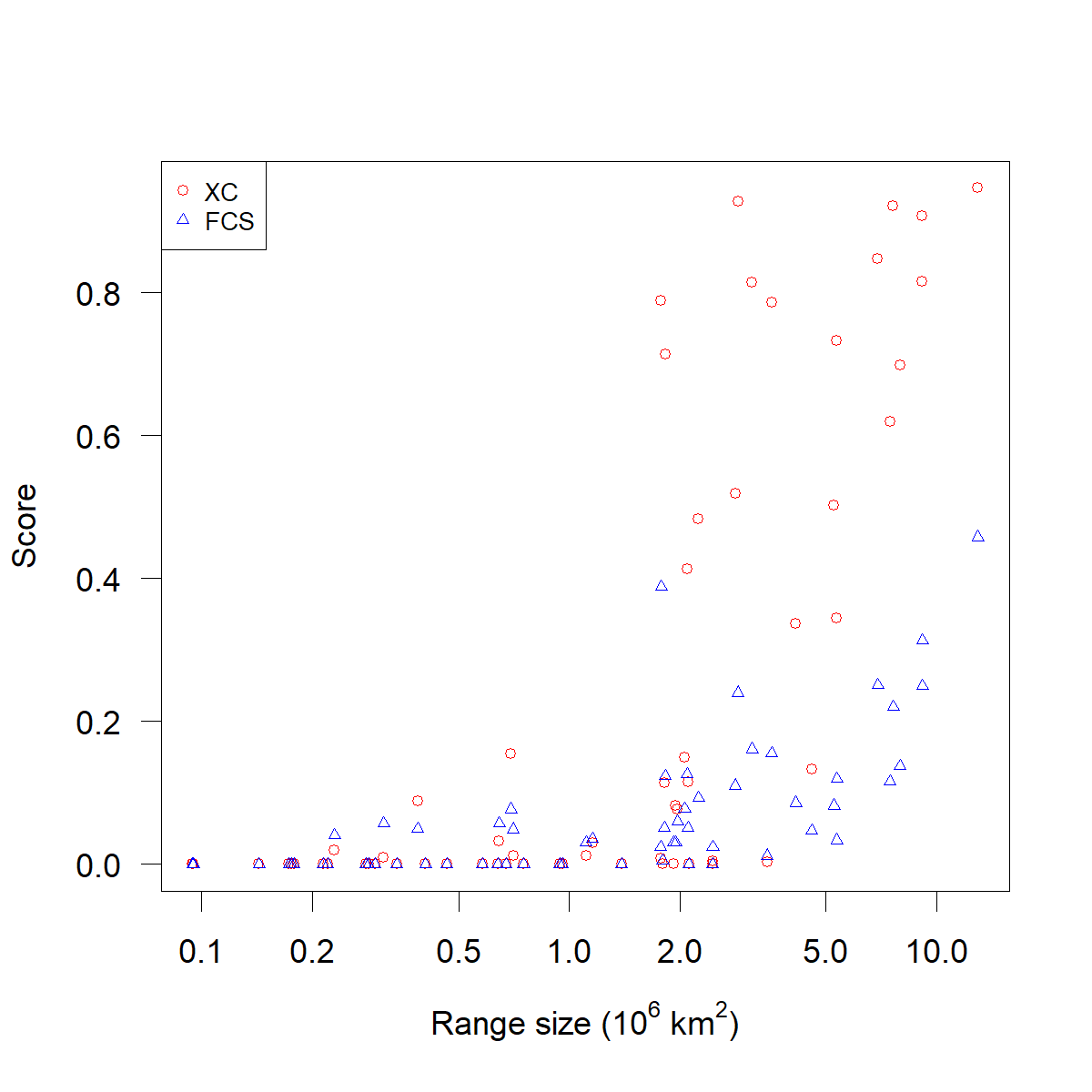


Fig. II: The relation between range size and the ex-situ conservation scores *XC* and *FCS* for wild *Vigna* species in Africa. The range of each species was predicted with a species distribution model and adjusted with a 250 km exclusion and a 100 km inclusion zones around all known occurrences.


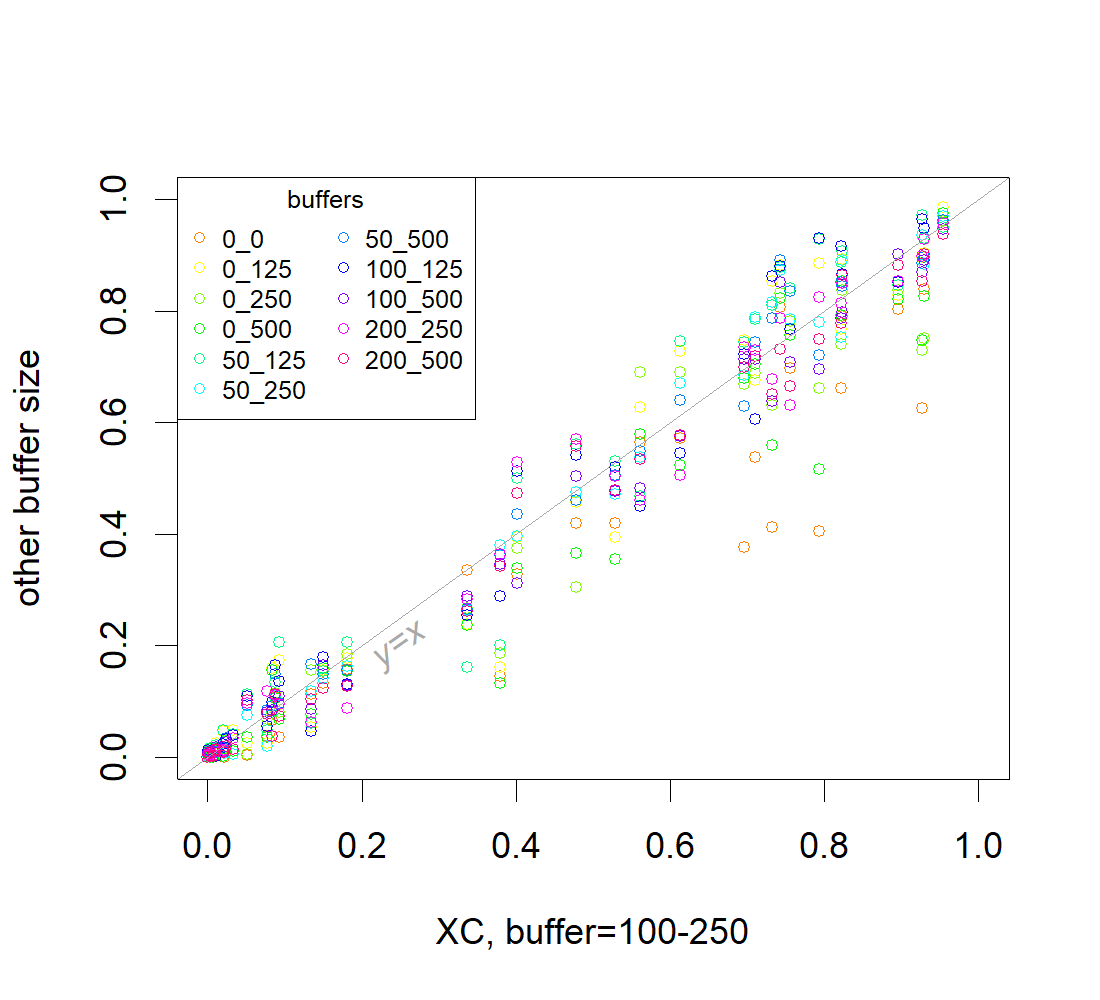


Fig. III: Sensitivity of the *XC* score to different inclusion-exclusion buffer sizes.


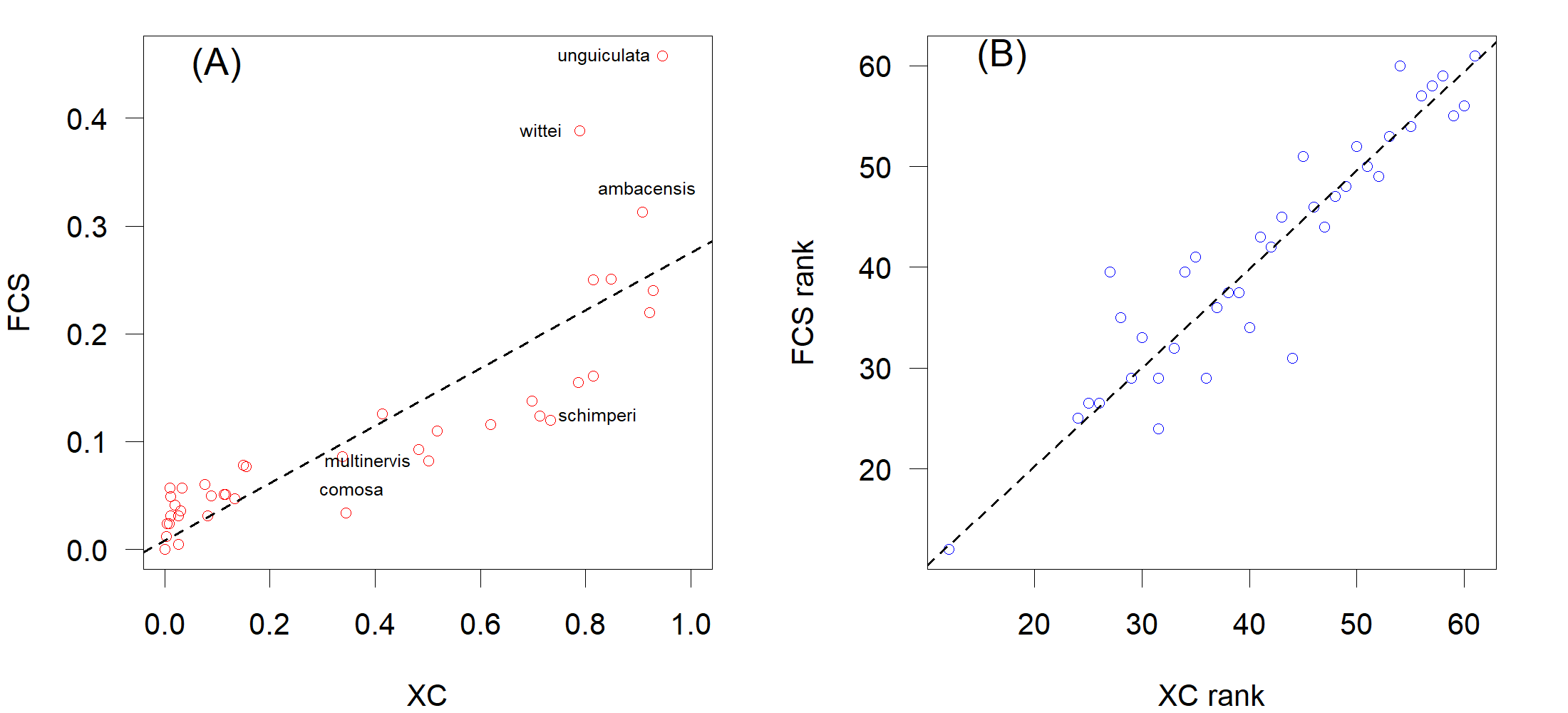


Fig. IV: Comparison of the *XC* and *FCS* scores for wild *Vigna* species in Africa. (A) Conservation score (Dashed line: *y=0.0082 + 0.26x*, *R*²=0.787) and (B) Rank conservation score (Dashed line is *y = x*).


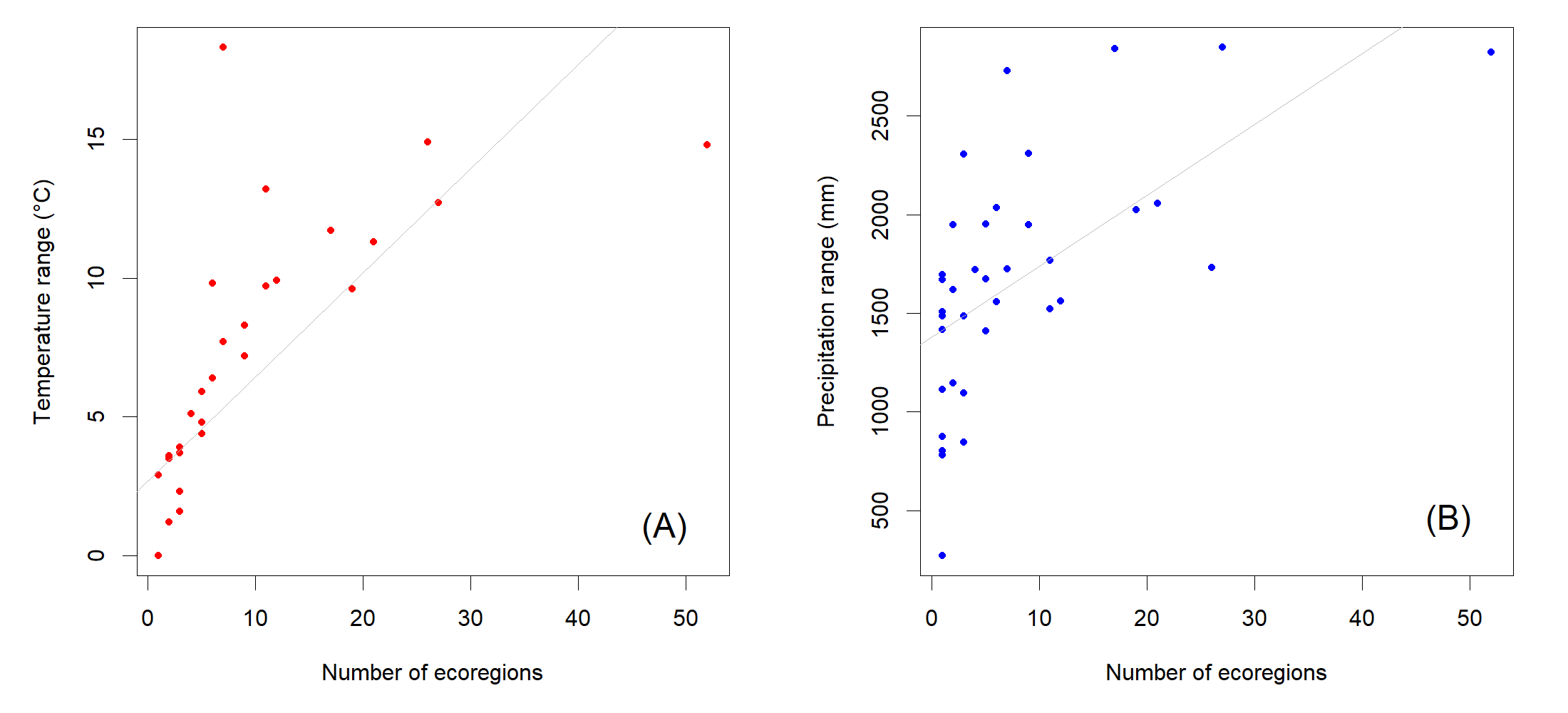


Fig. V: Temperature (A) and precipitation (B) range versus the number of ecoregions covered by wild *Vigna* species in Africa. Ecoregions are from (Olson & Dinerstein, 2002).


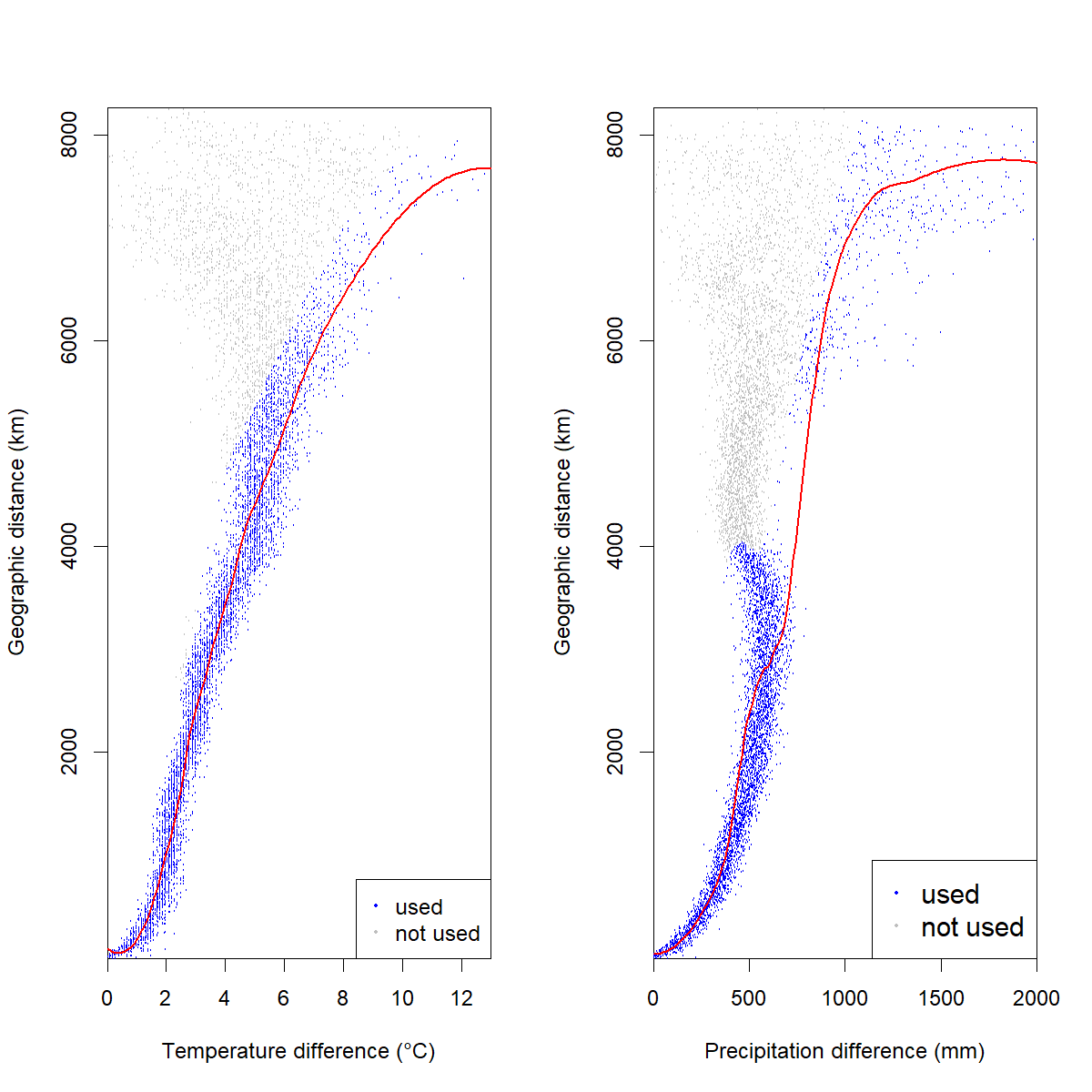


Fig. VI: Transformation of the two environmental distances (temperature and precipitation) to their equivalent expected geographic distance.
